# Adaptive oncogenesis of β-catenin emerges from declining liver tissue integrity

**DOI:** 10.1038/s41467-026-76373-y

**Published:** 2026-08-10

**Authors:** Abhinav Illendula, Charles K. Hewett, Yuki Hayata, Lauren S. Strathearn, Guoshun He, Laia Carballo-Botinas, Sara Navas-Sanz, Maria Niqui-Capdevila, Avril Garcia-Santos, Rocío Bernabé-Pérez, Alejandra Bustillo-Bustos, Ani Mikoyan, Eleanor Helm, Marina Gonzalez-Sorribes, Nicola de Prisco, Aasthika Das, Yuanguangyan Zhang, David Rossell, Michael Slifker, Naiara Akizu, Beicheng Sun, Joan Font-Burgada

**Affiliations:** 1 Cancer Signaling and Microenvironment Program, Fox Chase Cancer Center, Philadelphia, PA USA; 2 Biomedical Sciences Graduate Program, Lewis Katz School of Medicine at Temple University, Philadelphia, PA USA; 3 Universitat de Barcelona, Barcelona, Spain; 4 Universidad Francisco de Vitoria, Madrid, Spain; 5 Department of Biological Sciences, University of Delaware, Newark, DE USA; 6 Department of Economics, Universitat Pompeu Fabra, Barcelona, Spain; 7 Biostatistics and Bioinformatics Facility, Fox Chase Cancer Center, Philadelphia, PA USA; 8 Raymond G. Perelman Center for Cellular and Molecular Therapeutics and Center for Brain Research In Development, Genetics and Engineering, The Children’s Hospital of Philadelphia, Philadelphia, PA USA; 9 Department of Pathology and Laboratory Medicine, Perelman School of Medicine, University of Pennsylvania, Philadelphia, PA USA; 10 Department of Hepatobiliary Surgery, The First Affiliated Hospital of Anhui Medical University, Hefei, China

**Keywords:** Cancer genetics, Hepatocellular carcinoma, Cancer microenvironment

## Abstract

Mutations are fundamental to oncogenesis, yet they cannot fully explain cancer progression, as oncogenic mutations frequently accumulate in healthy tissues without immediate malignancy. In the liver, β-catenin mutations are highly prevalent in hepatocellular carcinoma but are widely considered weak or cooperative drivers, raising the question of how their oncogenic potential is contextually regulated. Aging and chronic injury are major determinants of cancer risk. However, how declining tissue integrity reshapes the fitness of cells harboring oncogenic mutations remains poorly understood. Here we show that mutant β-catenin as a single genetic event confers negative fitness in the healthy liver of male mice, where mutant hepatocytes fail to clonally expand and undergo progressive attrition associated with oxidative and endoplasmic reticulum stress. In contrast, as liver tissue integrity declines during chronic injury or aging, cells carrying the same β-catenin mutation clonally expand and promote tumorigenesis, a process linked with activation of the AKT-NRF2 axis. These findings demonstrate that β-catenin mutations acting as single genetic lesions are maladaptive under normal liver homeostasis but become adaptive as tissue integrity deteriorates. This tissue-level shift reshapes the oncogenic fitness landscape, relaxing the constraints that normally prevent tumor initiation.

## Abstract (web summary)

Despite being a common driver of hepatocellular carcinoma (HCC), oncogenic β-catenin is ineffective at initiating liver cancer on its own. Here the authors show that β-catenin mutations as a single genetic event confer negative fitness in the healthy liver, while driving clonal expansion and tumorigenesis as liver tissue integrity declines with chronic liver disease or aging.

## Introduction

Although mutations play an undeniable role in cancer, their accumulation alone is insufficient to fully explain the natural history of the disease. Mutations accumulate linearly over time^1–3^, while cancer incidence grows exponentially with age^4^. Moreover, healthy tissues, even at a young age, are peppered with cells harboring well-characterized oncogenic mutations^5–7^. Thus, although numerous cells throughout the body eventually acquire oncogenic mutations that persist within tissues, the vast majority never develop cancer. Our current understanding of what factors govern this decades-long latency and eventual failure in aged tissues is highly limited.

As a major risk factor for cancer, aging is accompanied by a progressive decline in tissue integrity, affecting structural organization, normal physiology, and stress responses. Such changes could reshape the fitness landscape experienced by cells harboring oncogenic mutations, altering whether these mutations are selectively disadvantageous, neutral, or adaptive compared to young, healthy tissues^8^. Similarly, chronic inflammatory conditions that compromise tissue integrity, which are closely associated with cancer development, may impose selective pressures favoring alternative genotypes better suited to persist and expand in such damaged microenvironments. Despite growing interest for investigating tissue context in cancer development, how declining tissue integrity alters the fitness of cells bearing oncogenic mutations remains largely unexplored.

Hepatocellular carcinoma (HCC) is the most common primary liver cancer, typically arising after decades of chronic liver disease (CLD), most often in cirrhotic livers. Deep sequencing of chronically injured and cirrhotic human livers has revealed widespread clonal expansions of hepatocytes carrying mutations associated with regenerative fitness, indicating that CLD reshapes the hepatocyte fitness landscape^2,3^. However, how these changes in the fitness landscape influence the selection of the oncogenic mutations that drive HCC remains unclear. A paradigmatic example is *CTNNB1* mutations causing the aberrant stabilization of the β-catenin and constitutive Wnt pathway activation. *CTNNB1* mutations are amongst the most frequent genetic alterations found in human HCC, occurring in 30% of cases^9^. Yet, despite their prevalence, the oncogenic role of β-catenin in liver cancer has long been debated.

Genetic studies in mice have consistently shown that activating β-catenin mutations in hepatocytes are insufficient to induce HCC. A conditional *Ctnnb1* allele that generates an in-frame deletion upon Cre-mediated recombination, mimicking activating mutations detected in cancer patients^10,11^, has been widely used to study oncogenic β-catenin. While widespread recombination of this allele in hepatocytes results in lethal hepatomegaly from hepatic hypertrophy a few weeks after birth^10,12^, mosaic activation using Adenovirus expressing Cre (Ad-Cre) fails to produce tumors^10^. Similarly, transposons overexpressing β-catenin mutants do not induce HCC^13–15^. Together, these studies have led to the prevailing view that oncogenic β-catenin requires cooperating genetic events to drive liver cancer^16–18^. Consistent with this view, recent work has shown that β-catenin oncogenesis is influenced by hepatic zonation and occurs only in combination with additional oncogenic alterations such as Myc overexpression or Arid2 loss^19,20^. Together, these studies have established a consensus that β-catenin acts as a weak and cooperative oncogene in the liver. Yet, paradoxically, β-catenin mutations are amongst the most frequent genetic alterations in human HCC.

In this work, we address this paradox by performing long-term clonal lineage tracing of hepatocytes bearing a highly prevalent *Ctnnb1* activating mutation found in human HCC in both the normal and chronically injured mouse livers. By coupling this tracing system with single-cell RNA sequencing, we find that in the healthy, young liver, β-catenin mutated hepatocytes are incapable of undergoing a single cell division, but instead last for months in the tissue in a long, maladaptive process of cellular attrition and elimination. In striking contrast, we find that as tissue integrity declines in the context of chronic liver disease, β-catenin mutated hepatocytes undergo clonal expansion and give rise to HCC without the need for cooperating oncogenes or tumor suppressors. Notably, a similar increase in fitness is observed in aged, but otherwise uninjured livers. Mechanistically, we identify elevated reactive oxygen species and endoplasmic reticulum stress as triggers of a maladaptive unfolded protein response, leading to cell elimination in intact tissue, whereas activation of the AKT-NRF2 axis accompanies the adaptive expansion of β-catenin mutated clones in chronic disease. Together, these findings show that β-catenin oncogenicity is not fixed but instead emerges with declining tissue integrity, identifying tissue state as a key determinant of oncogenic fitness.

## Results

### β-Catenin mutations acting as single genetic lesions confer negative fitness in the healthy liver

To precisely study the fate of hepatocytes acquiring *Ctnnb1* activating mutations in a normal liver context, we developed a clonal lineage tracing system using the previously described *Ctnnb1*^*ex3fl/+*^ knock-in mice^10^. These mice are wild-type (WT) unless exon 3 is deleted by the action of Cre recombinase, which generates a constitutively active β-catenin in the endogenous locus, preserving the same expression regulation. Thus, when used in heterozygosity, it accurately replicates β-catenin mutagenesis in human hepatocytes. *ROSA26*^*LSL-tdTomato/+*^ (*R26*^*tdT*^) reporter mice^21^ were combined with the *Ctnnb1*^*ex3fl/+*^ allele to allow for clonal lineage analysis by permanently labelling recombined hepatocytes. AAV8-TBG-iCre (AAV-Cre) was selected to induce the recombination of both alleles because of its low cell toxicity and immune response, leaving the liver tissue unperturbed. These viruses express Cre recombinase specifically in hepatocytes by virtue of AAV8 preferential liver tropism^22^ in conjunction with the hepatocyte-specific TBG promoter^23^. We titrated viral doses to randomly infect only ~1–3% of hepatocytes, ensuring that the liver remained normal, with most recombined hepatocytes being surrounded by WT hepatocytes, mimicking as closely as possible the rare and stochastic acquisition of mutations in humans.

**Fig. 1:**
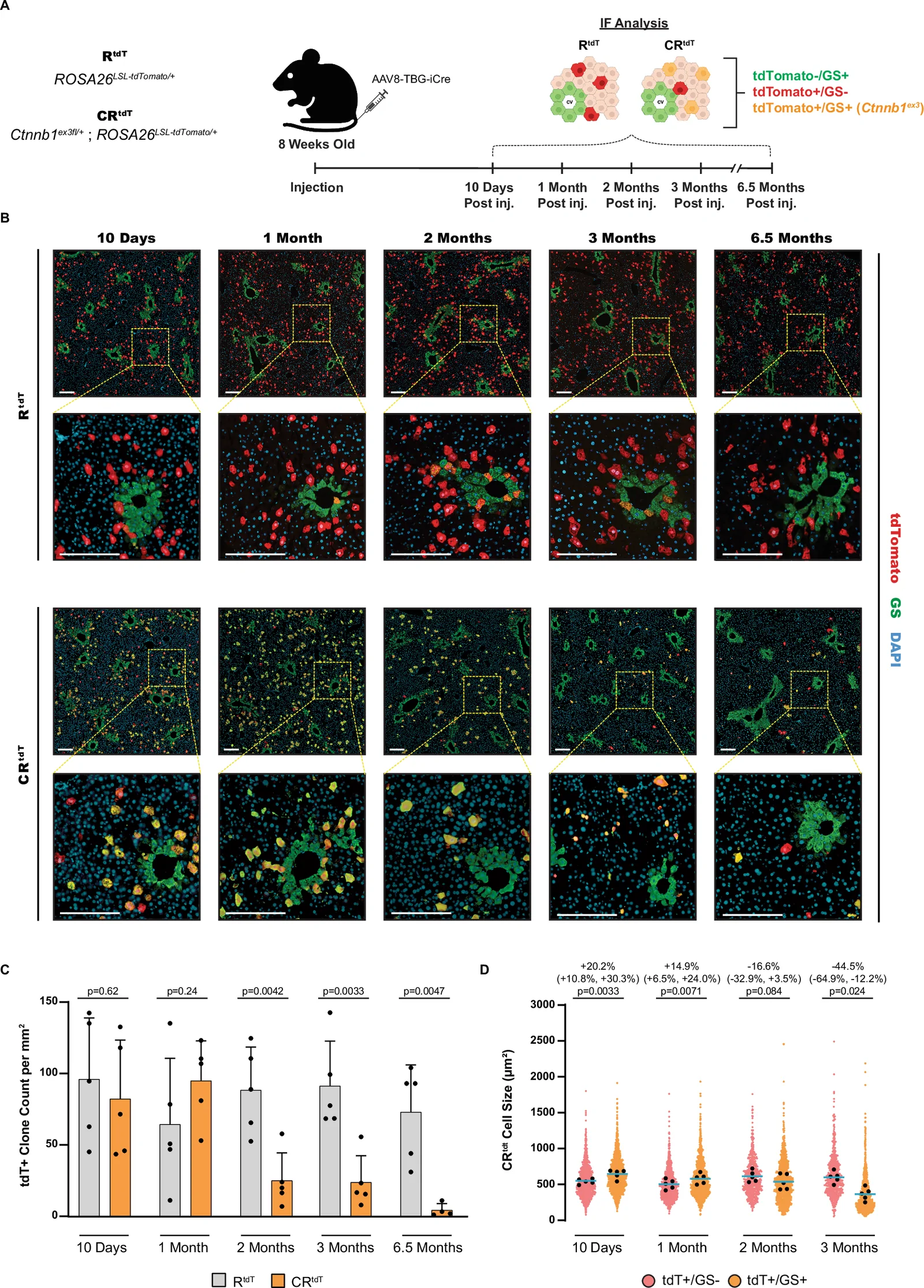
β-catenin mutation acting as a single genetic lesion confers negative fitness in the young healthy liver. **A** Schematic of the clonal lineage tracing system strategy used to express tdTomato only (R^tdT^) or in combination with heterozygous exon 3 deletion from endogenous β-catenin (CR^tdT^). Each mouse was injected with 1 × 10^10^ genomic copies of AAV8-TBG-iCre (AAV-Cre) to achieve ~1–3% of hepatocytes recombined. CV = Central Vein, IF = Immunofluorescence. **B** Representative images of immunofluorescence for tdT (tdTomato) and GS (β-catenin activity marker) on liver sections at the indicated timepoints post-AAV injection. Yellow-dashed boxes: area displayed in higher magnification. Scale bars = 200 µm. DAPI, 4′,6-diamidino-2-phenylindole. **C** Quantification of the number of tdT^+^ hepatocytes per mm^2^ at the indicated timepoints post-AAV injection. Each point represents the mean count from a mouse. Each bar represents the average of the mice means +SD. Statistical significance was determined between R^tdT^ and CR^tdT^ groups at each timepoint by performing an unpaired, two-tailed t-test using the mean of the areas counted from each mouse. *n* = 5 for all groups but 6.5-month CR^tdT^ (*n* = 4). **D** Quantification of cell size at the indicated timepoints post-AAV injection of tdT^+^ hepatocytes in CR^tdT^ mice. Each point represents either a tdT^+^;GS^-^ or tdT^+^;GS^+^ hepatocyte as indicated. Black dots are mouse means; blue lines indicate the mean of mouse means. Statistical significance between GS^-^ and GS^+^ cell size was determined by fitting a linear mixed effects model with a likelihood-ratio test. Significance of pairwise comparisons was determined using post-hoc comparisons from the fitted mixed-effects model, with two-sided tests. Percentage change (top) and 95% CI (bottom) of GS^+^ vs. GS^-^ cell size is listed above each relevant timepoint. *n* = 5 mice. **A** was created in BioRender. Hewett, C. (2026) https://BioRender.com/ukshf1x. Source data are provided as a Source Data file.

We applied this system to determine the fate of β-catenin mutated hepatocytes in the normal liver by injecting AAV-Cre into eight-week-old *R26*^*tdT*^ (R^tdT^) control and *Ctnnb1*^*ex3fl/+*^; *R26*^*tdT*^ (CR^tdT^) mice and sacrificing at various timepoints after virus injection for clonal analysis (Fig. 1A). Immunofluorescence of tdTomato (tdT) showed that the number of labeled hepatocytes in control mice did not change over time, and with no signs of proliferation, as expected based on the known low rate of hepatocyte turnover in the normal liver^24^. Since glutamine synthetase (GS) is a reliable read-out of Wnt pathway activity in hepatocytes^25–27^, we used a GS specific antibody to identify endogenous pericentral zone 3 hepatocytes and ectopic activation of β-catenin in tdT^+^ hepatocytes in AAV-Cre injected CR^tdT^ mice (Fig. 1B). 10 days after viral injection, these mice showed a mixture of tdT^+^ (~40%) and double tdT^+^;GS^+^ (~60%) hepatocytes, indicating different recombination efficiencies of both alleles. However, at 1 month after injection, the vast majority (~95%) of tdT^+^ hepatocytes were also GS^+^, suggesting that within a few weeks, most infected hepatocytes ended up recombining both alleles. When compared with control mice at 1 month, there was no significant difference in the number of labeled hepatocytes, nor was there any apparent major cellular phenotype, including any clonal expansions. Strikingly, at 2 months after injection, the number of tdT^+^;GS^+^ (*Ctnnb1*^*ex3*^) hepatocytes plummeted, reaching a minimum at 3 months and remaining very low onwards (Fig. 1C). Zonal analysis of the same data showed an initial infection bias towards zone 3 hepatocytes, but no significant differences in attrition rate between zones were detected over time (Supplementary Fig. 1). The average cell size of *Ctnnb1*^*ex3 *^hepatocytes increased 1 month after AAV-Cre administration but from that time point onwards, *Ctnnb1*^*ex3 *^hepatocytes displayed a significant reduction in cell size accompanying the decline in cell numbers (Fig. 1D). These observations indicate that in the normal liver, oncogenic activation of β-catenin in hepatocytes triggers cellular alterations that lead to a long process of profound morphological changes and eventual cell elimination.

### β-Catenin mutations acting as single genetic lesions drive clonal expansion and tumorigenesis during aging and chronic liver disease

By mimicking *CTNNB1* mutations similarly to how they are acquired in humans, we not only confirmed previous observations but also found that these mutations confer negative fitness in the normal liver, leading to elimination of mutant hepatocytes and deepening the paradox. However, in humans, β-catenin-driven HCC does not arise in the context of a normal liver but rather in chronically injured tissue. Thus, to test whether oncogenic *Ctnnb1* mutations confer a selective advantage during CLD, we adapted our clonal lineage tracing system to incorporate CLD as an experimental variable. To model clonal selection of *Ctnnb1*^*ex3*^ hepatocytes in the context of CLD, we implemented the *MUP-uPA* with high-fat diet (HFD) model, which has been extensively characterized and used to model metabolic dysfunction-associated steatohepatitis (MASH)^28–30^. This model offers several advantages for inducing *Ctnnb1*^*ex3*^ hepatocytes in a background of chronic liver injury. First, it recapitulates key histological features of human MASH, including hepatocyte ballooning and pericellular and bridging fibrosis in the context of obesity^30^. Second, while most *MUP-uPA* + HFD mice eventually develop spontaneous liver tumors, they largely remain tumor-free until at least 9 months of age. Beyond this point, tumors are mainly adenomas (70%), with only a minority classified as true HCC^30^. Molecular analysis uncovered the very low mutational burden of tumors in this model without any alterations in genes commonly mutated in human HCC^28^. Together, these features define a temporal window, from 5 weeks to approximately 6-8 months of age, during which the liver displays a MASH phenotype in the absence of spontaneous HCC tumors, providing an optimal window for clonal analysis.

**Fig. 2:**
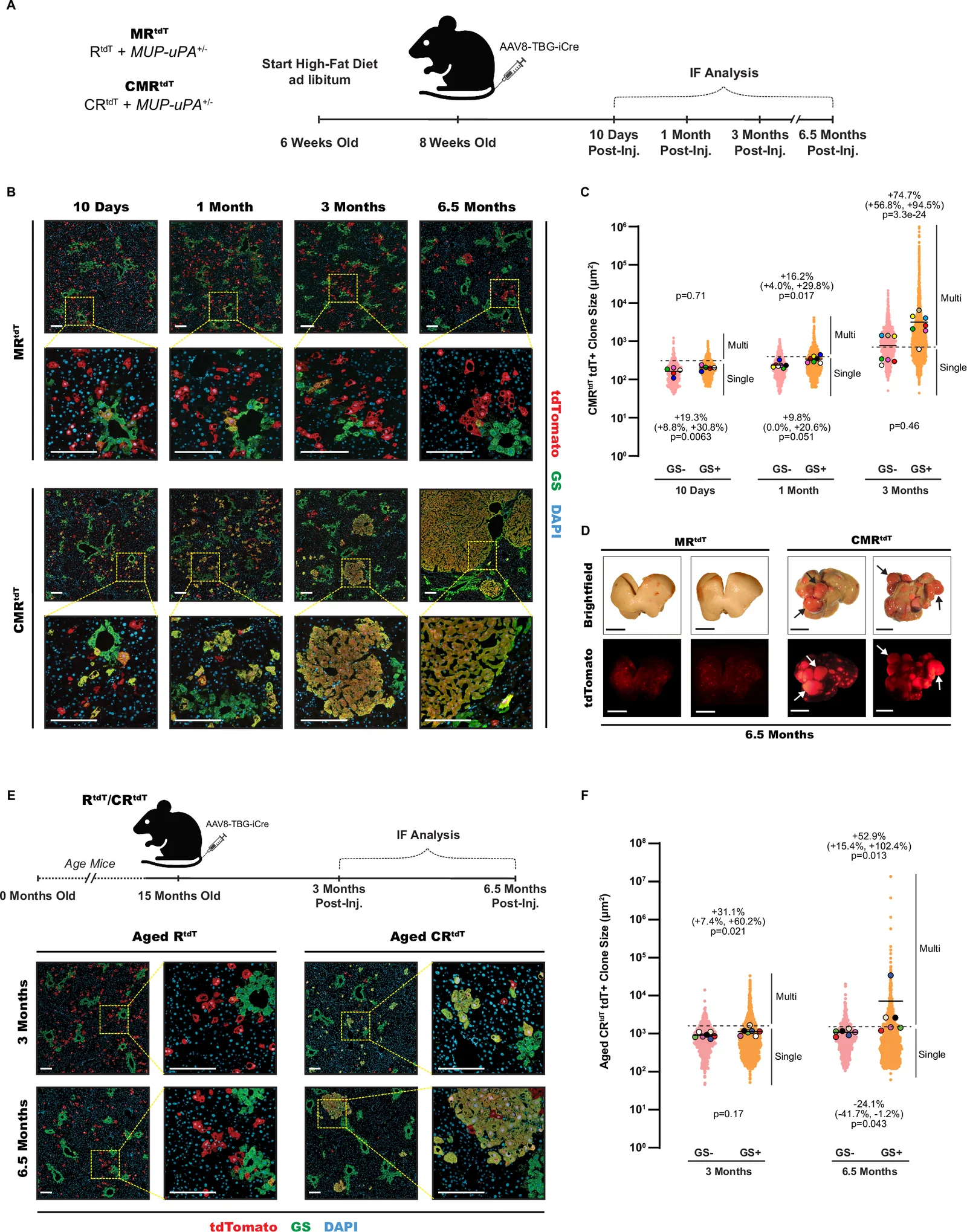
β-Catenin mutation acting as a single genetic lesion drives clonal expansion and tumorigenesis during aging and chronic liver disease. **A** Schematic of the clonal lineage tracing system in the context of chronic liver disease (*MUP-uPA* + HFD). **B** Representative immunofluorescence images of CR^tdT^ and CMR^tdT^ liver samples at the indicated timepoints post-AAV injection stained for tdT and GS. Yellow-dashed boxes indicate the area displayed in higher magnification. Scale bars = 200 µm. **C** Quantification of tdT^+^;GS^-^ (GS^-^) and tdT^+^;GS^+^ (GS^+^) clone sizes in CMR^tdT^ liver samples. Points are individual clones; colored dots are mouse means; black lines indicate the mean of mouse means. Same color indicates same mouse. *n* = 5, 7, and 7 at 10 days, 1 month, and 3 months, respectively. Clones were classified as single or multiple (multi) using per mouse size thresholds determined empirically from scanned stained tissue sections and the average of the thresholds is marked with a dashed line as a visual reference. Statistical significance between GS^-^ and GS^+^ clone size in each class was determined by fitting a linear mixed effects model with a likelihood-ratio test. Significance of pairwise comparisons was determined using post-hoc comparisons from the fitted mixed-effects model, with two-sided tests. Percentage change, 95% CI, and *p*-value of GS^+^ vs. GS^-^ clone size in each class are listed (Multi: above, Single: below). **D** Representative macroscopic brightfield and corresponding tdT fluorescence liver pictures of MR^tdT^ and CMR^tdT^ mice at 6.5 months post-AAV injection. Arrows highlight representative tumors and their corresponding tdTomato fluorescence. Scale bars = 1 cm. *n* = 7 and 6 for CR^tdT^ and CMR^tdT^, respectively. **E** Schematic of the clonal lineage tracing system in the context of aged R^tdT^ or CR^tdT^ mice and representative immunofluorescence images at the indicated timepoints post-AAV injection stained for tdT and GS. Scale bars = 200 µm. **F** Quantification and analysis of tdT^+^;GS^-^ (GS^-^) and tdT^+^;GS^+^ (GS^+^) clone sizes in the aged CR^tdT^ liver model as done in Fig. 2C. *n* = 7 and 6 mice at 3 months and 6.5 months, respectively. Panels A and E were created in BioRender. Hewett, C. (2026) https://BioRender.com/t3qttns. Source data are provided as a Source Data file.

To take advantage of this window, we generated *Ctnnb1*^*ex3/+*^; *MUP-uPA*; *R26*^*tdT*^ (CMR^tdT^) and control *MUP-uPA*; *R26*^*tdT*^ (MR^tdT^) mice. These mice were placed on a high-fat diet at six weeks of age and injected with AAV-Cre at 8 weeks (Fig. 2A). In the control MR^tdT^ mice, tdT^+^ hepatocytes increased in cell size due to lipid droplet accumulation. Over time, these hepatocytes randomly and moderately proliferated (Fig. 2B), consistent with previous observations using a different lineage tracing system^29^. Although *Ctnnb1*^*ex3*^ hepatocytes in CMR^tdT^ mice initially showed similar morphological features at 1 month after AAV-Cre injection, clones gradually increased in cell size and underwent significant clonal expansion by 3 months (Fig. 2B-C), correlating with increased Ki67 positivity (Supplementary Fig. 2). By 6.5 months, visible tumors were present in all mouse livers (Fig. 2B-D). Interestingly, not all *Ctnnb1*^*ex3*^ hepatocytes underwent clonal expansion. Instead, many showed signs of cellular attrition as observed in the normal liver (Fig. 2B-C). Consistently, at 6.5 months after AAV-Cre injection, when visible tumors were detected, most *Ctnnb1*^*ex3*^ hepatocytes had been eliminated except for tumors and a few residual single *Ctnnb1*^*ex3*^ hepatocytes (Fig. 2B). Notably, CR^tdT^ mice fed with only HFD for 3 months did not show significant clonal expansions (Supplementary Fig. 3A-C).

To determine whether additional oncogenes or tumor suppressors were mutated in tumors from 6.5-month CMR^tdT^ mice, we performed whole-exome sequencing. A very low SNV/indel burden was detected across the nine tumors analyzed, with a median of only nine coding-altering variants, with no significant recurrent mutation events in coding genes across tumors (Supplementary Fig. 4). We next assessed similarity of these tumors with *CTNNB1*-mutated human HCC. To this end, we integrated RNAseq data from CMR^tdT^ tumors with a previously published comparative transcriptomic dataset of mouse HCC models and human HCCs^28^ (Supplementary Fig. 5). This analysis revealed that CMR^tdT^ tumors, along with *Ctnnb1*-mutated STAM tumors, exhibited the closest transcriptomic similarity to *CTNNB1*-mutated human HCC. Taken together, these results indicate that *Ctnnb1*^*ex3*^ hepatocytes can give rise to bona fide HCC in the context of CLD without the requirement for additional oncogenic mutations.

To test whether the increased fitness conferred by oncogenic β-catenin during CLD extended to other contexts of tissue decline, we induced recombination in aged R^tdT^ and CR^tdT^ mice by administering AAV-Cre at 15 months of age (Fig. 2E). Livers were analyzed 3 and 6.5 months later, revealing a similar pattern of clonal expansion as seen in CMR^tdT^ mice, albeit with reduced clonal size (Fig. 2F). These results support a model in which CLD is analogous to accelerated aging, reshaping the hepatic fitness landscape and favoring clonal selection of hepatocytes harboring activating β-catenin mutations.

### β-Catenin–mutant hepatocytes in the intact liver adopt a stress-locked transcriptional state

**Fig. 3:**
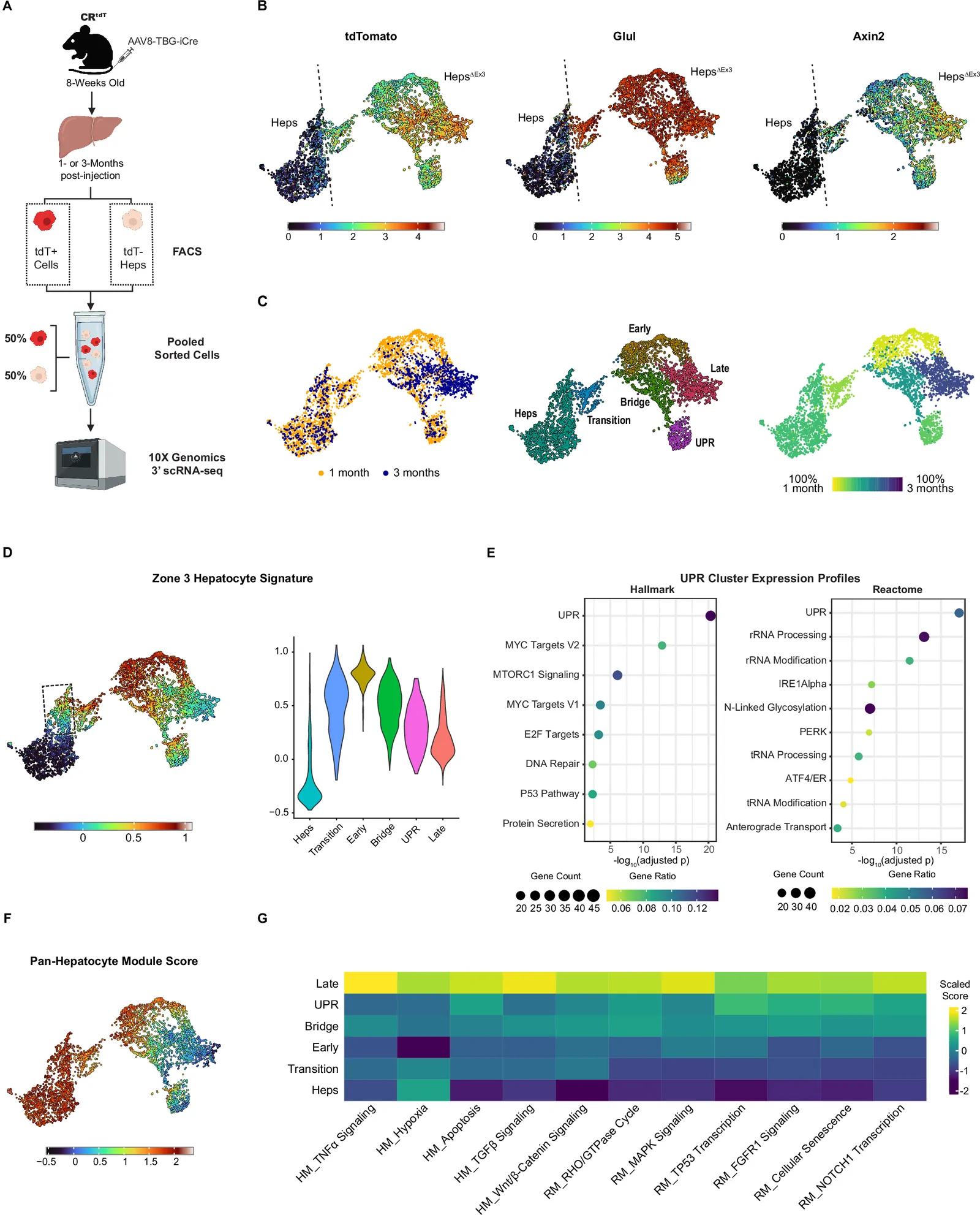
β-Catenin–mutant hepatocytes in the intact liver undergo maladaptive transcriptional reprogramming preceding elimination. **A** Schematic illustrating the experimental design used to generate single-cell transcriptomes of WT hepatocytes and *Ctnnb1*^*ex3*^ hepatocytes from the normal liver. This experiment was performed with three mice at 1 month and two mice at 3 months post-AAV injection, independently processed. **B** UMAP plots showing expression of tdTomato, *Glul* (GS), and *Axin2* from the dataset obtained as depicted in (**A**). The dotted line demarcates the boundary of WT hepatocytes and *Ctnnb1*^*ex3*^ hepatocytes. **C** Distribution of cells based on time point origin (left plot), subsequent clustering of populations based on gene expression profiles (middle plot), and time point enrichment within these clusters (right plot). **D** UMAP plot (left) showing enrichment of a zone 3 gene signature and relative enrichment levels for each cluster identified in Fig. 3C depicted in a violin plot (right). The dotted box on the UMAP plot highlights endogenous zone 3 hepatocytes. **E** Pathway enrichment of the UPR cluster using Hallmark and Reactome gene sets. Enrichment was assessed using a one-sided over-representation test. **F** UMAP visualization of a pan-hepatocyte gene signature. **G** Heatmap of select Hallmark (HM) and Reactome (RM) pathways enriched in the Late cluster across all cell clusters in the dataset. Panel A was created in BioRender. Hewett, C. (2026) https://BioRender.com/88t0qqm. Source data are provided as a Source Data file.

To gain insight into the molecular mechanisms by which oncogenic β-catenin induces hepatocyte elimination in the normal liver, we performed single-cell RNA sequencing (scRNAseq). For this, we FACS sorted *Ctnnb1*^*ex3*^ hepatocytes (tdT^+^) and WT hepatocytes (tdT^-^) isolated from CR^tdT^ mouse livers at 1-month (*n* = 3) and 3-months (*n* = 2) post AAV-Cre injection. For each of the mice analyzed, both populations of hepatocytes were remixed in a 50% proportion and loaded in a single well of a 10x Genomics Next Gem Chip. Using our prior experience, we remixed the hepatocytes post-sorting^31^, ensuring that we would have enough *Ctnnb1*^*ex3*^ hepatocytes and control hepatocytes to perform a robust analysis (Fig. 3A). Expression of tdTomato, *Glul* (GS), and *Axin2* unambiguously distinguished the two main clusters of WT hepatocytes (Heps) and *Ctnnb1*^*ex3*^ hepatocytes, as clearly visualized in the UMAP plot (Fig. 3B). Based on time points and gene expression profiles, we identified five clusters within the *Ctnnb1*^*ex3*^ hepatocyte population (Fig. 3C). The Transition cluster consists mainly of *Ctnnb1*^*ex3*^ hepatocytes (tdT^+^) from the 1-month timepoint, which co-express markers of zone 3 hepatocytes (*Glul* and *Axin2*) along with typical periportal zone 1 markers (*Gls2* and *Hal*) (Supplementary Fig. 6A). This co-expression pattern indicates that these *Ctnnb1*^*ex3*^ hepatocytes likely originated from zone 1 hepatocytes, which requires a longer transition toward the *Ctnnb1*^*ex3*^ hepatocyte state due to their greater initial transcriptomic difference from zone 2/3 hepatocytes. Their enrichment at the 1-month time point is consistent with a delayed adaptation process and the transient nature of this cluster. The Early cluster consists of *Ctnnb1*^*ex3*^ hepatocytes from the 1-month timepoint (Fig. 3C) and shows the strongest enrichment for canonical zone 3 hepatocyte markers. To confirm this, we applied a gene signature derived from normal mouse zone 3 hepatocytes and found that *Ctnnb1*^*ex3*^ hepatocytes in the Early cluster displayed hyperactivation of this signature, which progressively declined in the remaining *Ctnnb1*^*ex3*^ hepatocytes clusters (Fig. 3D). However, this hyperactivation was driven by just a subset of signature genes, suggesting that oncogenic β-catenin selectively amplifies part of the zone 3 hepatocyte transcriptional program, while other subprograms typical of zone 3 hepatocytes remain unengaged (Supplementary Fig. 6B). The Bridge cluster (Fig. 3C) consists of *Ctnnb1*^*ex3*^ hepatocytes that do not show significant enrichment for any specific gene set. These hepatocytes showed a decrease in the gene expression profile found in the Early cluster, with a concomitant slight upregulation of genes characteristic of the two downstream *Ctnnb1*^*ex3*^ hepatocyte clusters, UPR and Late. As its name suggests, the UPR cluster (Fig. 3C) is defined by the specific upregulation of genes involved in the unfolded protein response (UPR) pathway (Fig. 3E). Interestingly, this is accompanied by the upregulation of genes involved in rRNA and tRNA metabolism, suggesting an adaptive response in *Ctnnb1*^*ex3*^ hepatocytes as an attempt to counteract the UPR-mediated suppression of translation (Supplementary Fig. 6C). Finally, the Late cluster (Fig. 3C) is composed primarily of *Ctnnb1*^*ex3*^ hepatocytes from the 3-month timepoint. Analysis of a pan-hepatocyte gene signature shows a progressive loss of hepatocyte identity in the Bridge, UPR, and especially Late clusters (Figs. 3F and S6D). In the Late cluster, this loss is complemented by the activation of a wide range of pathways (Figs. 3G and S6E). Additional pathway signatures, such as Golgi anterograde transport and activation of TNFα, MAPK, and p53, suggest that these morphologically distinct *Ctnnb1*^*ex3*^ hepatocytes are adapted to sustained cellular stress. Interestingly, multiple developmental signaling pathways, including TGF-β, Wnt, Notch, and FGF are also simultaneously upregulated in this cluster.

Taken together, these observations suggest that oncogenic β-catenin induces an initial mis-reprogramming of hepatocytes into a hyperactive zone 3-like state. This state leads to ER stress, UPR activation, and the collapse of mature hepatocyte identity with the emergence of an ineffective, stress‑adapted, “limbo” state, preceding cellular elimination.

### Oxidative and ER stress drive negative fitness in β-catenin–mutant hepatocytes

**Fig. 4:**
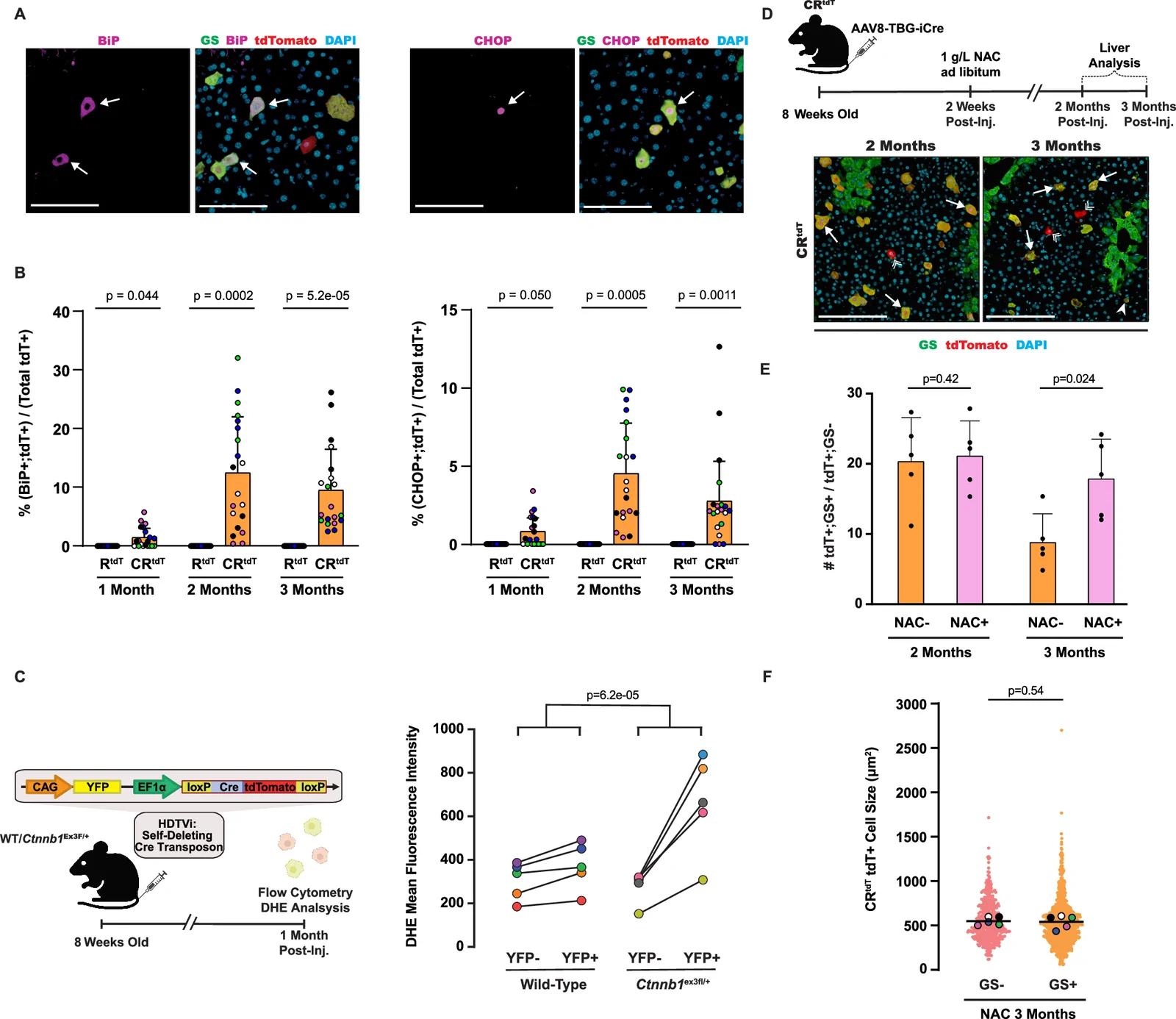
Oxidative and ER stress drive the negative fitness of β-catenin–mutant hepatocytes in the intact liver. **A** Representative immunofluorescence images of a CR^tdT^ mouse 3 months post-AAV injection stained for CHOP or BiP with GS and tdTomato. White arrows: tdT^+^;GS^+^ cells positive for CHOP or BiP. Scale bars = 200 µm. **B** %tdT^+^ cells that are also CHOP^+^ or BiP^+^ from samples in Fig. 1C-D. Each point represents an area counted within a lobe, and each set of colored points correspond to the same mouse. Four areas were counted per mouse. Each bar represents the average of the mice means +SD. Statistical significance was determined by performing an unpaired, two-tailed t-test using the mean of the areas counted from each mouse. *n* = 5. **C** Experimental design used to quantify ROS activity in wild-type and *Ctnnb1*^*ex3*^ hepatocytes. Each colored pair represents one mouse, with the YFP^-^ and YFP^+^ values from the same mouse connected. Statistical significance was determined by performing an unpaired, two-tailed t-test between the YFP^+^/YFP^-^ fold-changes for each genotype. *n* = 5 for each genotype. **D** NAC treatment schematic and representative immunofluorescence images 2 and 3 months post-AAV injection stained for tdT and GS. White arrows: tdT^+^;GS^+^ cells. Arrowheads: small tdT^+^;GS^+^ cells. Double-arrowheads: tdT^+^ only cells. Scale bars = 200 µm. **E** Clone counts of tdT^+^;GS^+^ cells normalized to tdT^+^;GS^-^ (tdT^+^ Only) cells in control CR^tdT^ mice (NAC-, same data as Fig. 1C-D) or treated with NAC (NAC + ) at the indicated timepoints. Each point represents the mean count from a mouse. Each bar represents the average of the mice means +SD. Statistical significance was determined by performing an unpaired, two-tailed t-test using the mice means. *n* = 5 for each group. **F** tdT^+^ cell size quantification in 3 month NAC treated CR^tdT^ mice. Each point represents either a tdT^+^;GS^-^ (GS^-^) or tdT^+^;GS^+^ (GS^+^) hepatocyte. Colored dots are mouse means; black lines indicate the mean of mouse means. Statistical significance was determined by fitting a linear mixed effects model with a likelihood-ratio test. Significance of pairwise comparisons was determined using post-hoc comparisons from the fitted mixed-effects model, with two-sided tests. *n* = 5. **C** was created in BioRender. Hewett, C. (2026) https://BioRender.com/18dm0ci. **D** was created in BioRender. Hewett, C. (2026) https://BioRender.com/t3qttns. Source data are provided as a Source Data file.

To validate UPR activation as a potential factor contributing to the progressive elimination of *Ctnnb1*^*ex3*^ hepatocytes, we performed immunostaining for canonical UPR markers specifically upregulated in the UPR cluster, including CHOP (*Ddit3*) and BiP (*Hspa5*). This analysis confirmed that a substantial fraction of *Ctnnb1*^*ex3*^ hepatocytes had strong UPR activation (Fig. 4A-B and Supplementary Fig. 7). Interestingly, both CHOP^+^ and BiP^+^ *Ctnnb1*^*ex3*^ hepatocytes peaked in samples at the 2-month time point, indicating that UPR activation precedes the morphological changes observed in *Ctnnb1*^*ex3*^ hepatocytes at 3 months. Given the established interplay between reactive oxygen species (ROS) and ER stress, where ROS can either trigger the UPR or synergize in a feedback loop^32^, we next assessed ROS levels in *Ctnnb1*^*ex3*^ hepatocytes and normal hepatocytes using the ROS-sensitive probe DHE (dihydroethidium). To do this, we employed a transposon co-expressing YFP and Cre/tdT, with Cre/tdT flanked by loxP sites. This configuration allows us to track integration events by YFP positivity, while Cre activity can be assessed by the loss of tdT (Fig. 4C). We delivered the transposon to both WT and *Ctnnb1*^*ex3fl/+*^ mice by hydrodynamic tail-vein injection (HDTVi) and 1 month later, isolated hepatocytes for DHE-based ROS measurements in YFP^+^ and YFP^-^ populations using flow cytometry. This analysis revealed markedly elevated ROS levels in *Ctnnb1*^*ex3*^ hepatocytes compared to normal hepatocytes (Fig. 4C).

To functionally link early ROS elevation with the subsequent UPR response at 2 months and cellular attrition in *Ctnnb1*^*ex3*^ hepatocytes at 3 months, we treated R^tdT^ control and CR^tdT^ mice with the antioxidant compound N-Acetyl-L-cysteine (NAC) in the drinking water and analyzed them at 2 and 3 months post-AAV injection (Fig. 4D). We measured clone counts in mice treated with NAC by normalizing the number of *Ctnnb1*^*ex3*^ hepatocytes with tdT^+^;GS^-^ (tdT^+^ Only) cells to account for any experimental variability between NAC-treated mice and untreated mice (Fig. 1C). At 2 months, the ratio of *Ctnnb1*^*ex3*^ hepatocytes matched the untreated samples (Fig. 4E). However, at 3 months post-injection, mice treated with NAC had a significantly higher ratio of *Ctnnb1*^*ex3*^ hepatocytes compared to untreated mice (Fig. 4E), indicating attenuated cell loss. Cell size measurements further showed that NAC treatment led to near-complete restoration of *Ctnnb1*^*ex3*^ hepatocyte cell size to levels comparable to normal hepatocytes within the same liver (Fig. 4F). We next measured the expression of the UPR markers CHOP and BiP in these samples but observed no significant differences (Supplementary Fig. 8). Overall, these results suggest that although NAC treatment delays oncogenic β-catenin-induced cell death and rescues cellular attrition in the normal liver, it is insufficient to resolve the underlying stress response and prevent cellular elimination.

As described above, *Ctnnb1*^*ex3*^ hepatocytes undergo dramatic morphological changes by three months after AAV-Cre administration, including a pronounced reduction in cell size (Fig. 1D). However, our scRNAseq analysis did not reveal any clear mechanism of cellular elimination (Fig. 3A and S9A). To gain a deeper insight into the basis of the cell size reduction, we isolated three populations of hepatocytes from CR^tdT^ mice at 3 months: tdT^+^ hepatocytes (*Ctnnb1*^*ex3*^), further subdivided into normal- and small-sized cells, and tdT^-^ normal hepatocytes. Electron microscopy analysis of these three populations showed a striking accumulation of autolysosomes in small tdT^+^ cells (Supplementary Fig. 9B-C) that were not seen in tdT^-^ or normal sized tdT^+^ hepatocytes. This suggests continuous autophagy as an additional mechanism of initial stress-adaptation and survival, that when prolonged, contributes to cellular attrition and elimination.

Collectively, these findings suggest that, in the normal liver, oncogenic activation of β-catenin in hepatocytes leads to elevated intracellular ROS, ER stress, and UPR activation, which together induce constitutive autophagy, cell size reduction and ultimately, hepatocyte elimination.

### Chronic liver disease shifts β-catenin–mutant hepatocytes into a pro-survival metabolic state

**Fig. 5:**
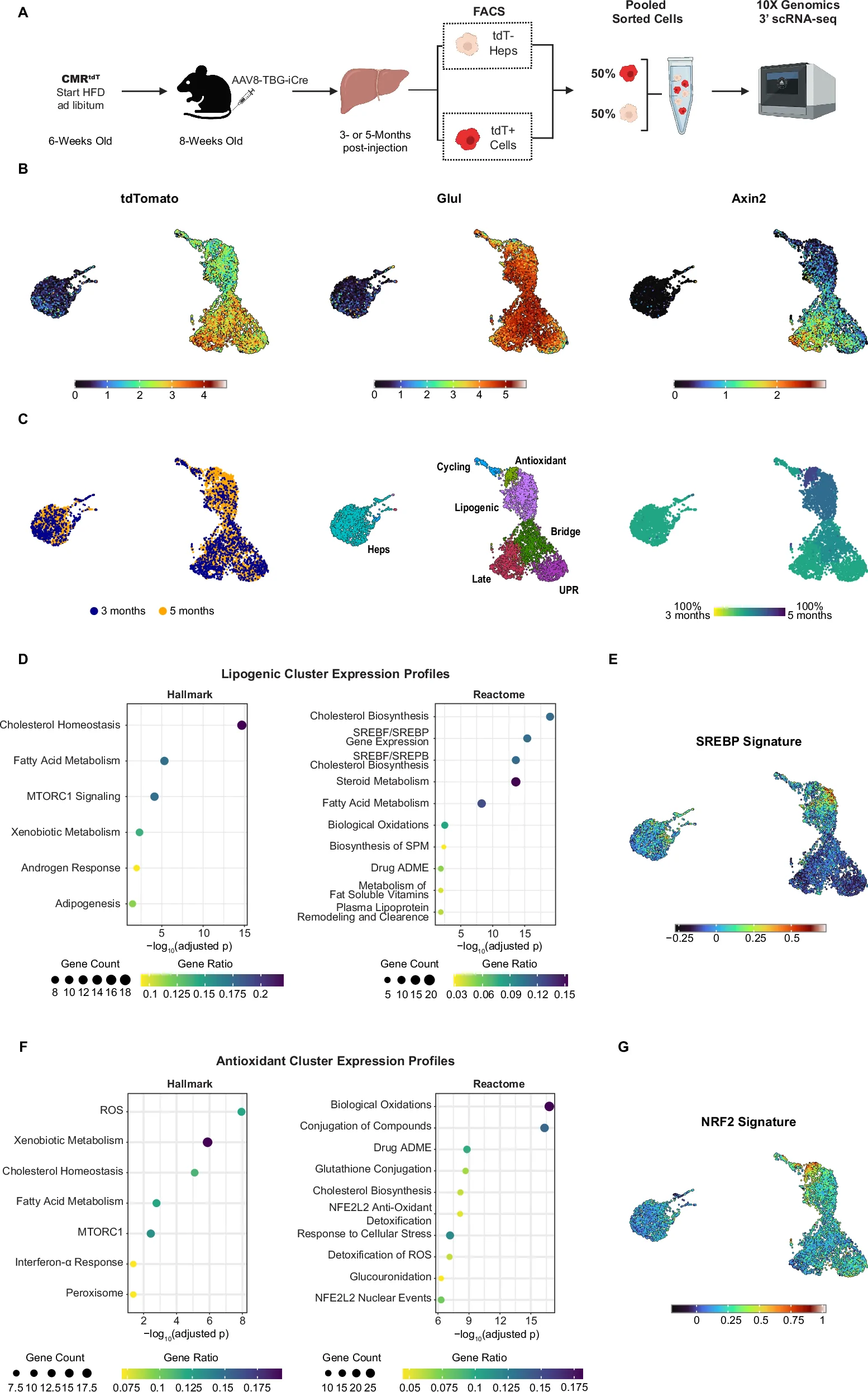
β-Catenin–mutant hepatocytes in the diseased liver adopt an antioxidant, pro-survival transcriptional state. **A** Schematic illustrating the experimental design used to generate single-cell transcriptomes of WT hepatocytes and *Ctnnb1*^*ex3*^ hepatocytes from damaged livers at 3 and 5 months post-AAV injection. This experiment was performed with two mice at both 3 and 5 months post-AAV injection, independently processed. **B** UMAP plots showing expression of tdTomato, *Glul* (GS), and *Axin2* from the dataset obtained as depicted in (**A**). **C** Distribution of cells based on time point origin (left plot), subsequent clustering of populations based on gene expression profiles (middle plot), and time point distribution within these clusters (right plot). **D** Pathway enrichment of the Lipogenic cluster for Hallmark and Reactome gene sets. Enrichment was assessed using a one-sided over-representation test. **E** UMAP visualization of the SREBP gene set signature. **F** Pathway enrichment of the Antioxidant cluster using Hallmark and Reactome gene sets. **G** UMAP visualization of an NRF2 gene signature. **A** was created in BioRender. Hewett, C. (2026) https://BioRender.com/88t0qqm. Source data are provided as a Source Data file.

To better understand the molecular mechanisms underlying clonal expansion induced by oncogenic β-catenin in hepatocytes within the context of CLD, we once again performed scRNAseq. For this, we FACS sorted *Ctnnb1*^*ex3*^ hepatocytes (tdT^+^) and WT hepatocytes (tdT^-^) isolated from CMR^tdT^ mouse livers at 3- and 5-months post AAV-Cre injection, with duplicates for each timepoint. For each mouse analyzed, both populations of hepatocytes were remixed in a 50% proportion and loaded in a single well of a 10x Genomics Next Gem Chip (Fig. 5A). Data was processed and analyzed in the exact same way as the data sets for the normal liver (Fig. 3). Expression levels of tdT, *Glul*, and *Axin2*, clearly differentiated normal hepatocytes and *Ctnnb1*^*ex3*^ hepatocytes cell clusters (Fig. 5B). Within *Ctnnb1*^*ex3*^ hepatocytes, several clusters were defined by their gene expression profiles (Fig. 5C). Interestingly, clusters Late, UPR, and Bridge previously defined in the normal liver were also present in CMR^tdT^ mice (Fig. 5C and Supplementary Fig. 10A-B). This finding is consistent with the histological observation that non-expanding *Ctnnb1*^*ex3*^ hepatocytes with reduced cell size were present in CMR^tdT^ mice (Fig. 2B).

Three unique *Ctnnb1*^*ex3*^ hepatocyte clusters were identified in CMR^tdT^ mice. Pathway analysis defined a Lipogenic cluster, where genes related to fatty acid and cholesterol biosynthesis were upregulated. Interestingly, the mTOR pathway and its downstream effector SREBF were identified as the likely drivers of this upregulation of lipid synthesis (Fig. 5D-E). Furthermore, when the Hallmark mTOR pathway was plotted as a signature on the UMAP, two distinct areas were enriched, corresponding to the Lipogenic and UPR clusters (Supplementary Fig. 10C). To confirm this bivalent mTOR response, we identified the mTOR pathway genes that were upregulated in the Lipogenic vs. UPR clusters and determined their overlap with the set of genes regulated by the SREBF or UPR pathways. Chronic mTOR activation has been shown to induce ER stress and UPR, implicating mTOR with paradoxical cellular stress^33,34^. These overlaps (Supplementary Fig. 10D) confirmed that different subsets of the mTOR pathway are engaged in these two types of *Ctnnb1*^*ex3*^ hepatocytes with dramatically different outcomes.

The Antioxidant cluster was identified through pathway analysis, which uncovered a high enrichment of upregulated genes involved in the ROS metabolic pathway (Figs. 5F and S10E) and cellular detoxification, with the transcription factor NRF2 (*Nfe2l2*) as the main likely driver of this gene expression pattern (Fig. 5F-G). Immunostaining with the canonical NRF2 target gene, *Nqo1*, revealed high levels of this enzyme in expanding *Ctnnb1*^*ex3*^ hepatocytes at 3 months (Supplementary Fig. 10F) and in visible tumors at 6.5 months (Supplementary Fig. 10G). Finally, a cluster of proliferating *Ctnnb1*^*ex3*^ hepatocytes was identified closely associated with the Antioxidant cluster (Fig. 5F and S10H). It is important to note that proliferating *Ctnnb1*^*ex3*^ hepatocytes were not present in the normal liver (Fig. 3). Temporally, although both 3- and 5-month timepoints were analyzed long after AAV administration, *Ctnnb1*^*ex3*^ hepatocytes within the Lipogenic and Antioxidant clusters were enriched with cells from the 5 months samples (Fig. 5C), suggesting that *Ctnnb1*^*ex3*^ hepatocytes with these phenotypes are associated with clonal expansion at later stages. To determine whether *Ctnnb1*^*ex3*^-derived tumors accumulated lipids due to high lipogenesis pathway activity, we performed Oil Red staining on 6.5-month CMR^tdT^ samples (Supplementary Fig. 11A). tdT^+^ tumors had reduced Oil Red staining intensity compared to the surrounding liver tissue, indicating that tumor cells lacked substantial lipid accumulation. However, subsequent IF analysis on the same samples revealed increased expression of markers of AKT activity (p-AKT S473, p-AKT T308, p-PRAS40 T246), fatty acid synthesis (FASN, SCD1), and cholesterol synthesis (HMGCS1), suggesting that lipid metabolism in these tumors is redirected toward supporting proliferation rather than lipid storage (Supplementary Fig. 11B).

NQO1 expression was used as readout for NRF2 activity given that *Nfe2l2* deletion has been reported to completely abolish NQO1 induction^35,36^. Analysis of 6.5-month CMR^tdT^ livers (Supplementary Fig. 10G) showed that nearly all visible tumors were NQO1-positive, whereas medium- and small-sized clones comprised a mixture of NQO1 positive and negative clones (Supplementary Fig. 12A) confirming association of NRF2 activity with expansion and tumorigenesis. Aged CR^tdT^ mice also showed clones at 6.5 months which were NQO1-positive (Supplementary Fig. 12B), suggesting that NRF2 is generally associated with proliferation in *Ctnnb1*^*ex3*^ hepatocytes when liver tissue integrity is compromised.

Taken together, these results indicate that *Ctnnb1*^*ex3*^ hepatocytes in the context of CLD still undergo the process of cellular attrition associated with UPR and reduction of cell size observed in the normal liver. However, a fraction of *Ctnnb1*^*ex3*^ hepatocytes distinctly upregulates lipogenic and antioxidant programs associated with cellular proliferation and clonal expansion that lead to tumorigenesis.

### NRF2 activity is required for the adaptive expansion of β-catenin–mutant hepatocytes in chronic liver disease

**Fig. 6:**
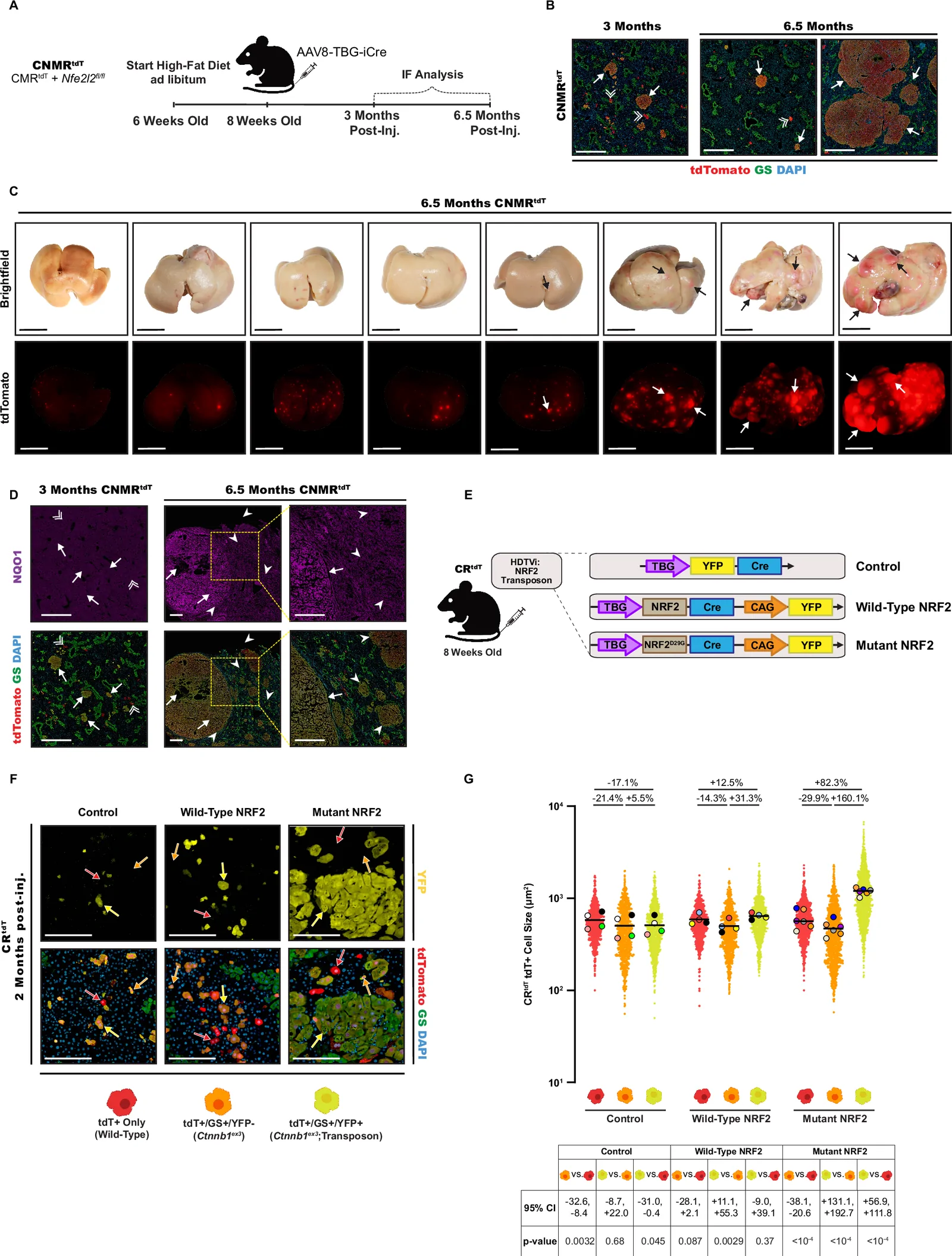
NRF2 enables the adaptive expansion of β-catenin–mutant hepatocytes. **A** Schematic illustrating the experimental setup to incorporate the deletion of *Nfe2l2* (NRF2) in CMR^tdT^ mice with chronic liver disease (CNMR^tdT^). **B** Representative immunofluorescence images of CNMR^tdT^ liver sections at the indicated timepoints post-AAV injection stained for tdT and GS. White arrows: tdT^+^;GS^+^ clones. Double-arrowheads: tdT^+^ only clones. Scale bars = 200 µm. *n* = 4 and 8 for 3 and 6.5 months, respectively. **C** Brightfield and fluorescent macroscopic liver images from CNMR^tdT^ mice collected at 6.5-month post-AAV injection. *n* = 8. Arrows: representative tdT^+^ macroscopic tumor. Scale bars = 1 cm. **D** Representative immunofluorescence pictures of NQO1 staining in CNMR^tdT^ mice described in Fig. 6B. Yellow-dashed boxes: area displayed in higher magnification. White arrows: large tdT^+^;GS^+^ clones positive for NQO1. Arrowheads: small tdT^+^;GS^+^ clones negative for NQO1. Double-arrowheads: tdT^+^;GS^-^ clones. Scale bars = 1 mm. *n* = 4 and 8 for 3 and 6.5 months, respectively. **E** Schematic of PiggyBac transposons to express wild-type, a constitutively active D29G-mutant form of NRF2, or control vector in *Ctnnb1*^*ex3*^ hepatocytes. **F** Representative immunofluorescence images of CR^tdT^ mice 2 months after injection with each of the three plasmids (**E**) stained for tdT, GS, and YFP. Red arrows: tdT^+^ only; orange arrows: tdT^+^;GS^+^;YFP^-^ hepatocytes (*Ctnnb1*^*ex3*^ hepatocytes); yellow arrows: tdT^+^;GS^+^;YFP^+^ hepatocytes (*Ctnnb1*^*ex3*^ hepatocytes + integrated transposon). Scale bars = 200 µm. **G** Cell size quantification of the three hepatocyte populations in CR^tdT^ mice injected with the indicated transposons and analyzed at 2 months post-HDTVi. Points are individual cells; colored dots are mouse means; black lines indicate the mean of mouse means. Same color indicates same mouse. Statistical significance between the three cell populations’ cell sizes was determined by fitting a linear mixed effects model with a likelihood-ratio test. Significance of pairwise comparisons was determined using post-hoc comparisons from the fitted mixed-effects model, with two-sided tests. Percentage change of cell size is listed above each comparison; 95% CI and *p*-values are listed in the table. *n* = 4, 4, and 6 for the control, wild-type, and mutant NRF2 transposons, respectively. **A** and **E** were created in BioRender. Hewett, C. (2026) https://BioRender.com/t3qttns. **F** and **G** were created in BioRender. Hewett, C. (2026) https://BioRender.com/rdcdc4s. Source data are provided as a Source Data file.

The observation that elevated ROS in *Ctnnb1*^*ex3*^ hepatocytes is associated with cell attrition in the normal liver, while upregulation of NRF2 signaling in *Ctnnb1*^*ex3*^ hepatocytes correlates with clonal expansion and tumorigenesis in the diseased liver, suggests that the antioxidant response may be a critical determinant of oncogenic *CTNNB1* outcomes in hepatocytes, an idea supported by previous clinical and experimental studies^37,38^. To determine whether NRF2 is required for the clonal expansion and tumorigenesis found in the context of chronic liver disease (Fig. 2A-D), we generated *Ctnnb1*^*ex3fl/+*^; *Nfe2l2*^*fl/fl*^; *MUP-uPA*; *R26*^*tdT*^ (CNMR^tdT^) mice. These mice were fed with HFD starting at 6 weeks of age, injected with AAV-Cre two weeks later, and sacrificed at 3 and 6.5 months after injection (Fig. 6A). Of the eight mice analyzed, five livers displayed rare small visible tumors 6.5 months after AAV administration. Surprisingly, numerous large tumors were found in the remaining three livers (Fig. 6B-C). However, nearly all large tumors were NQO1 positive, whereas medium and small sized clones in these livers were predominantly NQO1 negative (Figs. 6D and S12C). Western blot and genotyping analyses of large tumors isolated from 6.5-month CNMR^tdT^ livers confirmed that these tumors had originally escaped *Nfe2l2* deletion and subsequently underwent strong positive selection (Supplementary Fig. 12D-E). Together, these findings indicate that NRF2 signaling is critical for efficient *CTNNB1*-driven oncogenesis in the context of chronic liver disease.

To confirm that elevated NRF2 activity, as observed in expanding clones during chronic liver disease, can overcome *Ctnnb1*^*ex3*^ hepatocyte attrition and elimination in the normal liver and promote clonal expansion and tumorigenesis, we generated three transposon vectors to either overexpress WT NRF2 or constitutively active NRF2 (NRF2^D29G^), and a control vector. YFP served as a readout of clonal NRF2 expression, along with Cre recombinase expression for deletion of *Ctnnb1* exon 3 (Fig. 6E). These transposons were delivered by HDTVi in CR^tdT^ and R^tdT^ mice at 8 weeks of age. While the control vector produced the expected *Ctnnb1*^*ex3*^ hepatocyte attrition regardless of YFP expression, WT NRF2 was able to fully normalize *Ctnnb1*^*ex3*^ hepatocyte cell size (Fig. 6F-G). Consistent with previous reports of synergistic induction of HCC when both mutated β-catenin and mutated NRF2 were overexpressed^38^, we observed enhanced survival of *Ctnnb1*^*ex3*^ + NRF2^D29G^ hepatocytes, with rapid clonal expansions strongly positive for NQO1 not seen in R^tdT^ mice with NRF2^D29G^ (Fig. 6F-G and Supplementary Fig. 13A-C). These results indicate that enhancing NRF2 signaling can switch the fate of *Ctnnb1*^*ex3*^ hepatocytes from elimination to oncogenicity in the normal liver, although this requires supraphysiological activity.

### AKT activation unlocks NRF2-dependent fitness of β-catenin–mutant hepatocytes

**Fig. 7:**
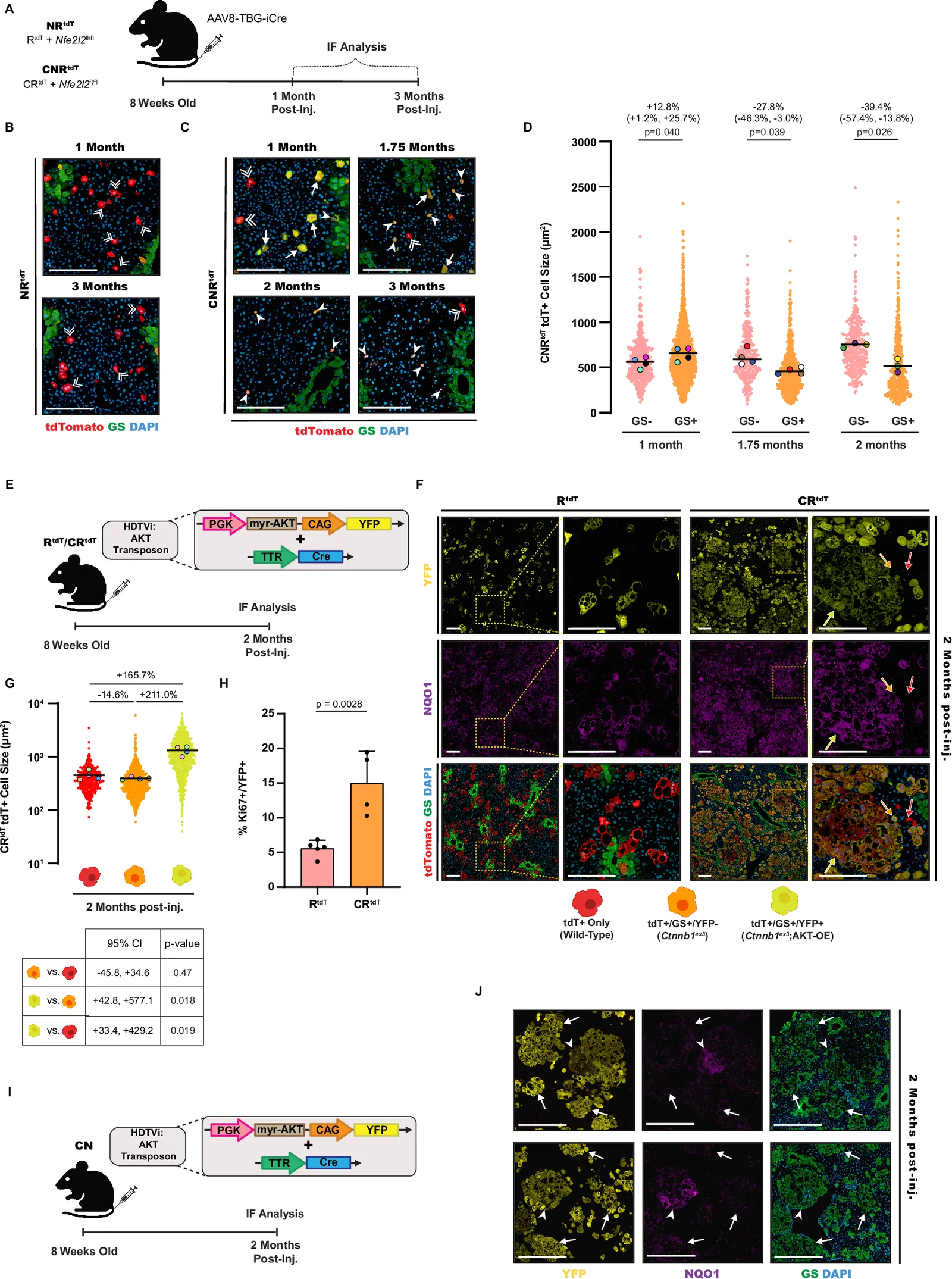
AKT–NRF2 signaling governs the adaptive fitness of β-catenin–mutant hepatocytes. **A** Schematic incorporating *Nfe2l2* (NRF2) deletion in R^tdT^/CR^tdT^ mice. Representative immunofluorescence images of NR^tdT^ (**B**) or CNR^tdT^ (**C**) mice stained for tdT and GS. White arrows: normal-sized tdT^+^;GS^+^ cells; arrowheads: small tdT^+^;GS^+^ cells; double-arrowheads: tdT^+^ only cells. Scale bar=200 µm. D) tdT^+^;GS^-^ and tdT^+^;GS^+^ cell size quantifications in CNR^tdT^ mice. Points are individual cells; colored dots are mouse means; black lines indicate the mean of mouse means. Same color indicates same mouse. Statistical significance was determined by fitting a linear mixed effects model with a likelihood-ratio test. Significance of pairwise comparisons was determined using post-hoc comparisons from the fitted mixed-effects model, with two-sided tests. Percentage change (top), 95% CI (middle), and *p*-value (bottom) of GS^+^ vs. GS^-^ cell size is listed above. *n* = 4, 4, and 3 for 1, 1.75, and 2 months, respectively. **E** Diagram depicting myr-AKT expression in *Ctnnb1*^*ex3*^ hepatocytes. **F** Representative immunofluorescence images of R^tdT^ and CR^tdT^ mice stained for tdT, GS, YFP, and NQO1. Yellow-dashed boxes: higher magnification area. Red arrows: tdT^+^ only; orange arrows: tdT^+^;GS^+^;YFP^-^ hepatocytes (*Ctnnb1*^*ex3*^ hepatocytes); yellow arrows: tdT^+^;GS^+^;YFP^+^ hepatocytes (*Ctnnb1*^*ex3*^ hepatocytes + integrated transposon). Scale bars = 200 µm. **G** Cell size quantification of the three hepatocyte populations described in (**F**), visualized similarly to (**D**). Statistical significance was determined by fitting linear mixed effects model with a likelihood-ratio test. Significance of pairwise comparisons was determined using post-hoc comparisons from the fitted mixed-effects model, with two-sided tests. Percentage change is above each comparison; 95% CI and *p*-value are in the table. *n* = 4. **H** %Ki67^+^ of YFP^+^ cells from samples in (**F**). Each point represents average %Ki67^+^ per mouse. Each bar represents mice average +SD. Statistical significance was determined using an unpaired, two-tailed t-test. *n* = 5, 4 for R^tdT^ and CR^tdT^, respectively. **I** Experimental schematic expressing myr-AKT in *Ctnnb1*^*ex3*^; *Nfe2l2*^*fl/fl*^ (CN) mice. **J** Representative immunofluorescence images of CN mice stained for GS, YFP, and NQO1. White arrows: GS^+^;YFP^+^;NQO1^-^ clones. Arrowheads: GS^+^;YFP^+^;NQO1^+^ clones. Scale bars=200 µm. *n* = 4. **A**, **E**, and **I** were created in BioRender. Hewett, C. (2026) https://BioRender.com/t3qttns. **F** and **G** were created in BioRender. Hewett, C. (2026) https://BioRender.com/rdcdc4s. Source data are provided as a Source Data file.

Paradoxically, although *Ctnnb1*^*ex3*^ hepatocytes in the normal liver display elevated ROS levels (Fig. 4C) and can be rescued by enforced NRF2 activity (Fig. 6), it remained unclear whether endogenous NRF2 is activated under these conditions or instead functionally constrained despite oxidative stress. To directly test the contribution of endogenous NRF2 to *Ctnnb1*^*ex3*^ hepatocyte survival in the normal liver, we generated *Ctnnb1*^*ex3fl/+*^; *Nfe2l2*^*fl/fl*^; *R26*^*tdT*^ (CNR^tdT^) and control *Nfe2l2*^*fl/fl*^; *R26*^*tdT*^ (NR^tdT^) mice, which were injected with AAV-Cre at eight weeks of age to clonally and simultaneously delete the *Nfe2l2* gene and exon 3 of *Ctnnb1* (Fig. 7A). tdT^+^ hepatocytes in control NR^tdT^ mice showed no apparent phenotype at 1 and 3 months (Fig. 7B). Liver tissues from CNR^tdT^ mice were analyzed at 1, 1.75, and 2 months after injection to assess NRF2 activity in *Ctnnb1*^*ex3*^ hepatocytes. These analyses confirmed the absence of detectable NQO1 expression, in contrast to the increased NQO1 expression observed in *Ctnnb1*^*ex3*^ hepatocytes from CR^tdT^ mice, indicating successful deletion of *Nfe2l2* (Supplementary Fig. 13D-G). *Ctnnb1*^*ex3*^;*Nfe2l2*^*-/-*^ hepatocytes at 1 month showed increased cell size (+12.8% GS+ vs. GS- cell size) similar to *Ctnnb1*^*ex3*^ hepatocytes at 1 month (Fig. 1D). However, at 1.75 and 2 months, *Ctnnb1*^*ex3*^;*Nfe2l2*^*-/-*^ hepatocytes were significantly reduced in cell size (−27.8% and −39.4%, respectively, Fig. 7C-D), which is comparable to *Ctnnb1*^*ex3*^ hepatocytes at the 3-month timepoint (Fig. 1D), indicating that NRF2 is engaged in *Ctnnb1*^*ex3*^ hepatocytes in the normal liver but at levels insufficient to quell the elevated oxidative stress. Thus, in the absence of NRF2, *Ctnnb1*^*ex3*^ hepatocyte elimination is accelerated.

Taken together, these findings suggest the existence of an NRF2 regulatory switch between normal and chronically injured liver, where NRF2 activity is constrained in the normal state but heightened in chronic liver disease, thereby altering the fate of hepatocytes with oncogenic β-catenin. A clue to the identity of the potential switch came from the observation that *Ctnnb1*^*ex3*^ hepatocytes in chronic liver disease showed marked upregulation of the SREBF-induced lipogenic program (Fig. 5), a signature of AKT pathway engagement. In addition, prior studies have shown a positive genetic interaction between *Ctnnb1* and *AKT*^39^, as well as AKT activation downstream of NRF2 activation^40^. Notably, AKT signaling in hepatocytes promotes lipogenesis, increased cell size, and lipid accumulation, primarily through activation of mTOR and SREBF^41^. While chronic mTOR activation can inhibit AKT through multiple mechanisms, including induction of the AKT inhibitor, TRB3, via CHOP signaling^42^, this effect can be offset by sustained AKT activation, as occurs during chronic inflammation and tissue damage^43^.To directly test whether AKT activation can lead to tumorigenesis in the clonal tracing system, we co-injected a transposon expressing low levels of a constitutively active form of AKT (myr-AKT) with a non-integrable transposon expressing Cre into R^tdT^ and CR^tdT^ mice (Fig. 7F). Clonal constitutive AKT activation in R^tdT^ mice led to the typical cellular phenotype of increased cell size, lipid accumulation, and limited clonal proliferation^44,45^ (Fig. 7F). In contrast, the same AKT activation in CR^tdT^ mice induced rapid clonal expansions, demonstrating that AKT activation can overcome the fitness disadvantage imposed by oncogenic *Ctnnb1* in the normal liver (Fig. 7G). Immunofluorescence analysis of these samples showed that AKT activated clones in CR^tdT^ mice had a significantly higher percent of Ki67^+^ cells compared to those in R^tdT^ mice (Fig. 7H), further demonstrating the synergistic interaction between AKT hyperactivation and oncogenic *Ctnnb1* in promoting hepatocyte proliferation. Importantly, AKT activation led to strong upregulation of NQO1 in both genotypes (Fig. 7F), indicating that endogenous AKT activation is sufficient to induce high levels of NRF2 activity. Consistent with this model, *Trib3* expression (TRB3), a known inhibitor of AKT, was upregulated in *Ctnnb1*^*ex3*^ hepatocytes in the UPR clusters found in both the normal and chronic liver damage scRNAseq datasets (Supplementary Fig. 14), potentially explaining the suppression of AKT–NRF2 signaling in this context.

To determine whether NRF2 activity is required for the proliferation of *Ctnnb1*^*ex3*^ hepatocytes paired with constitutive AKT activation in the normal liver, we co-injected the myr-AKT transposon with a non-integrating Cre-expressing transposon in *Ctnnb1*^*ex3/+*^; *Nfe2l2*^*fl/fl*^ (CN) mice and observed clonal expansions comparable to those seen in an NRF2 WT background (Fig. 7I-J). These results indicate that AKT driven *Ctnnb1*^*ex3*^ hepatocyte clonal expansion in the normal liver can bypass NRF2 activity requirement.

## Discussion

Cancer initiation and progression are far more complex than once thought. Rather than a purely stochastic accumulation of mutations, tumor development reflects an evolutionary process in which mutant cells compete under the pressure of the specific tissue fitness landscape. The widespread presence of cells harboring oncogenic mutations in normal healthy tissues indicates that the default outcome is neutral or negative selection, tilting the balance towards tumor suppression. Aging or chronic tissue inflammation induce profound changes to the tissue fitness landscape reshaping the selective pressures acting on the different cellular genotypes available. Yet, how declining tissue integrity during aging or chronic injury or inflammation alter the fitness of specific oncogenic mutations remains largely unknown.

Oncogenic mutations are commonly thought of as binary switches that confer fixed cellular properties. Accordingly, combinations of cancer associated mutations are considered as additive or multiplicative interactions whose phenotypic consequences depend on the cell type or subpopulation in which they occur. This conceptual framework has strongly influenced how the oncogenic role of *CTNNB1* activating mutations, one of the most frequent genetic alterations in human HCC, has been interpreted. Experimental studies in mice have established the prevailing model in which β-catenin is a weak oncogene in the liver, requiring cooperation of other oncogenic events to drive HCC development. Recent studies have refined this model by showing that mutant β-catenin combined with Arid2 loss or Myc overexpression produces different outcomes depending on hepatic zonation, establishing that genetic alterations interact with intrinsic hepatocyte subpopulation features in the normal liver to drive oncogenicity^19,20^. However, this framework assumes oncogenic potential as the sole consequence of genotype and cell identity interaction, without accounting for how aging or chronic liver disease dramatically change tissue-level selective constraints.

Here, we show that the identical β-catenin mutation provides opposing fitness to hepatocytes depending on the tissue state. In the normal liver, activating β-catenin mutations are not merely non-oncogenic; they are maladaptive, imposing negative selection that drives the progressive elimination of mutant hepatocytes regardless of zonation. However, in chronic liver disease or aging, the same β-catenin mutation acting as a single genetic event, supports clonal expansion and ultimately HCC development. This demonstrates that oncogenic potential is not determined solely by the interaction between mutation and cell identity, but by how tissue-level changes modify the repertoire and frequency of cellular signaling states in which mutations interact, opening new phenotypic opportunities. Thus, fundamental changes in the microenvironment alter intrinsic cellular signaling cascades that enable this adaptive response. The apparent need for oncogenic β-catenin to cooperate with additional alterations in the normal liver does not reflect the need for more oncogenic substrate, but rather the scarcity of permissive cellular states within the intact liver.

This tissue state dependent fitness framework reconciles previous reports demonstrating seemingly conflicting outcomes of oncogenic *Ctnnb1* activation in the normal liver depending on the number of hepatocytes recombined. Studies inducing oncogenic *Ctnnb1* in all hepatocytes reported hepatomegaly and liver failure. These phenotypes were attributed to widespread hepatocyte hypertrophy rather than proliferation, as well as extensive cell death^10,12^, consistent with the initial hepatocyte hypertrophy and subsequent attrition and elimination observed in the long-term lineage tracing presented here. Similarly, the lack of tumorigenesis observed in studies using sparse recombination^10^ is consistent with the finding that *Ctnnb1*-mutant hepatocytes are eliminated in the normal liver. In more recent work^46,47^, the observation that tumors developed when the proportion of hepatocytes induced to carry the mutation was increased to just below the threshold for acute lethality supported the notion that β-catenin activating mutations have limited oncogenic potential in the liver. However, based on our model and data, the widespread loss of *Ctnnb1*-mutant hepatocytes predicted to occur in livers containing high numbers of mutant hepatocytes would trigger local tissue damage and regenerative responses, effectively mimicking features of chronic liver disease. Thus, the apparent weak oncogenic potential of β-catenin in this sub-lethal context of the normal liver can be reinterpreted as a consequence of altered tissue integrity and changes in the fitness landscape experienced by *Ctnnb1*-mutant hepatocytes. This negative selection model provides a potential explanation for the scarcity of CTNNB1 mutations in the normal healthy human liver^2,48^.

A key determinant of the context-dependent fitness conferred by *Ctnnb1* activating mutations is an intrinsic NRF2 switch that operates differently depending on the normal versus diseased liver. In the normal liver, although *Ctnnb1*^*ex3*^ hepatocytes display high ROS and ER stress, endogenous NRF2 activity is insufficient to maintain viability, leading to progressive attrition and elimination. The ability of NRF2 overexpression or AKT activation to rescue survival and promote clonal expansion indicates that NRF2 activity is actively constrained in this context. One potential mechanism for this constrain is chronic UPR, which induces TRB3, a potent inhibitor of AKT, as well as β-catenin induced negative feedback loops, including heightened GSK3β activity, a known NRF2 repressor. Together, this circuitry wires β-catenin mutant hepatocytes into a state of chronic mTOR activation, UPR signaling, and suppressed AKT-NRF2 activity, yielding negative fitness in the normal liver.

In chronic liver disease, this regulatory switch flips and activates AKT in a subset of *Ctnnb1*^*ex3*^ hepatocytes. These hepatocytes acquire elevated lipogenic and antioxidant programs, directly inhibiting GSK3β while promoting SREBF-mediated lipogenesis. Overall, this AKT activation mitigates cellular stress and increases survival. This switch provides a mechanistic explanation for why co-mutations of *NFE2L2* or *KEAP1* with *CTNNB1* in human HCC can be advantageous^38,49^: constitutive NRF2 activation establishes a positive feedback loop that activates AKT signaling^40^, stabilizing the adaptive state required for β-catenin oncogenicity. Notably, only supraphysiologic NRF2 activity is sufficient to initiate this loop, as wild-type NRF2 enhances survival but does not completely overcome the inhibitory constraints in the normal liver. While additional pathways induced from chronic injury may also cooperate with oncogenic β-catenin, these findings identify the AKT-NRF2 axis as a dominant molecular gate that determines whether β-catenin mutated hepatocytes are positively or negatively selected.

Notably, not all *Ctnnb1*^*ex3*^ hepatocytes expanded even in CLD, and many were eliminated as in the normal liver, indicating that only a subset of hepatocytes adopts an AKT–NRF2 permissive signaling state capable of unleashing β-catenin oncogenicity. We propose that CLD and aging generate a diversity of cellular states, with their frequency and identity shaped by disease etiology, local tissue degeneration, and the specific signaling pathways engaged. As a result, only hepatocytes with the right AKT-NRF2 wiring are positively selected when *CTNNB1* is mutated.

Our analysis of β-catenin mutations in the liver provides a direct demonstration that oncogenic potential is strongly influenced by declining tissue integrity. Instead of cancer depending on the accumulation of oncogenic events until a fixed threshold is crossed, our findings support a model in which the threshold itself is dynamically set by tissue state. In healthy, young tissues the threshold is high, and even potent oncogenic mutations are insufficient to drive tumorigenesis on their own. As tissues age or sustain chronic injury and inflammation, the threshold declines, allowing single oncogenic events to initiate clonal evolution. These threshold shifts reflect changes in the available space of genotype-cell state interactions. While under normal tissue homeostasis, the repertoire of cellular states is narrow by selection for optimal tissue function, as tissue integrity erodes, cellular states diversify, expanding the likelihood that oncogenic mutations become selectively advantageous. This model finally explains why *CTNNB1* mutations are not oncogenic in the normal liver but are highly prevalent in HCC patients who are largely suffering from chronic liver disease. Further work will be needed to determine how other common cancer-associated mutations reshape the cellular fitness landscape in the liver under different tissue contexts, and how preventive and therapeutic strategies might be optimized considering these complex interactions.

## Methods

### Mice and in vivo treatments

All animal experiments were performed according to the protocol 26-06 approved by the Fox Chase Cancer Center Institutional Animal Care and Use Committee (IACUC) and complies with all relevant ethical regulations. Unless otherwise specified, animals were kept on standard laboratory chow and drinking water ad libitum in a temperature and humidity-controlled, 12 h light cycle facility. Food was removed for 3–4 h before sacrifice. Male mice were used for all experiments, and each mouse strain was maintained on a C57BL/6 background. All mice were obtained from their cited sources: *ROSA26*^*LSL-tdTomato/+*^ (Jackson Laboratory, #007909), *Ctnnb1*^*ex3fl/+*^^50^ *MUP-uPA*^51^, and *Nfe2l2*^*fl/fl*^ (Jackson Laboratory, #025433). *MUP-uPA* mice (MR^tdT^, CMR^tdT^, NMR^tdT^, and CNMR^tdT^), as well as CR^tdT^ and R^tdT^ mice, were maintained on High-Fat Diet (HFD) (Bio-Serv; S3282) ad libitum starting at 6 weeks old. For NAC experiments, mice were treated with 1 g/L N-Acetyl-L-cysteine (NAC) (Sigma; A7250) in drinking water ad libitum for 10 weeks. Mice that harbored tumors were monitored daily and sacrificed if tumor burden comprised >10% of the mouse’s body weight, the mouse lost 20% of its body weight after injection, or if the mouse exhibits signs of distress or pain. No mice in this study exceeded these limits.

### AAV and plasmid vectors

AAV/DJ8:TBG-iCre (Vector Biolabs; VB1724) was administered by tail-vein injection at 1 × 10^10^ genomic copies per mouse (3 × 10^10^ genomic copies per mouse for electron microscopy experiments and CR^tdT^ mice for single cell RNA sequencing) in 200 µL PBS. All PiggyBac transposon and hyperactive PiggyBac transposase vectors were constructed by VectorBuilder. Transposons (30 µg/mouse) and transposase (15 µg/mouse) were diluted in saline and injected intravenously by hydrodynamic tail-vein injection (HDTVi) such that the total injection volume was equivalent to 10% of the mouse body weight, i.e., a 25 g mouse received 2.5 ml of DNA solution.

### Liver tissue collection, preservation, processing, and imaging

Mouse livers were harvested following intracardiac perfusion with Heparin (Sigma-Aldrich; H3149) in PBS, followed by Zinc-Formalin (StatLab; BFZ-1), then post-fixed overnight in Zinc-Formalin. After PBS washes, livers were incubated in 100 mM Tris (pH 9.5) (Teknova; T1095) with 10 mM DTT (Sigma; 43816) for 2 h at room temperature. Tissues were cryoprotected by overnight incubations in 15% and then 30% sucrose, and embedded in Tissue-Tek® OCT (Sakura, 4583) and stored at −80 °C. For immunofluorescence tissue staining, 8μm frozen sections were placed on Fisherbrand™ Superfrost™ Plus slides. Antigen retrieval was performed by incubating the slides in citrate buffer (ScyTek; CBB999) at 96 °C for 1 h, followed by PBS washes and 20 min incubation in 0.1% Triton X-100 (Sigma; T8787) in PBS. After additional PBS washes, sections were blocked in 2% donkey serum (Jackson ImmunoResearch; 017-000-121) in 0.1% Tween-20 (Sigma; P1379) in PBS for 30 min. Primary antibodies (see Supplementary Table 1) were diluted in blocking buffer and incubated overnight at 4 °C. Slides were washed with 0.1% Tween-PBS and incubated with species-appropriate secondary antibodies for 2 h at room temperature. After Tween-PBS and PBS washes, slides were briefly rinsed in water and 70% ethanol, then incubated in 0.1% Sudan Black (Alfa Aesar; J62268) for 20 min. Excess stain was removed with extensive washing in 0.02% Tween-PBS. Sections were counterstained with DAPI (1 μg/mL) (Sigma; D9542) for 10 min at room temperature and mounted in Mowiol. Imaging was performed using a Zeiss AxioObserver and processed with Zeiss Zen software. Whole-slide scans were acquired using a Nanozoomer S60 (Hamamatsu). Tyramide signal amplification (TSA) staining (Thermo Fisher Scientific; B40933) was performed with samples stained for CHOP to enable the use of primary antibodies of the same species. TSA was performed according to the manufacturer’s instructions, followed by a second antigen retrievbal. This was followed by the normal immunofluorescence protocol described above. For Oil Red O (ORO) staining, ORO working solution was prepared by diluting 60 mL of ORO stock solution, 0.3 g Oil Red O (Sigma; O0625-25G) in 100 mL Isopropyl Alcohol, with 40 mL of distilled water and filtered after sitting for 30 min. 10 µm frozen sections were placed on Fisherbrand™ Superfrost™ Plus slides, then washed for 5 min in tap water, rinsed briefly in 50% isopropyl alcohol, and incubated for 15 min in ORO working solution. After ORO incubation, slides are briefly washed in 50% isopropyl alcohol followed by tap water before incubation with hematoxylin for 100 s, after which slides are washed with tap water. Slides were mounted using aqueous mounting media Clearmount Solution.

### Primary mouse liver cell isolation, purification, ×and FACS

To isolate hepatocytes and tdT⁺ cells for single-cell RNA-sequencing and electron microscopy, mouse livers were perfused via the portal vein at 37 °C. Perfusion began with 50 mL of Ca²⁺/Mg²⁺-free HBSS supplemented with 10 mM HEPES and 0.5 mM EGTA (Alfa Aesar; J60767-AE) at 4 mL/min, followed by Ca²⁺/Mg²⁺-containing HBSS with 10 mM HEPES and 4 mg/mL Liberase TM (Sigma; 5401127001) at the same rate. Perfusion continued until clear signs of digestion occurred in the liver parenchyma under the liver capsule. Livers were excised, the gallbladder removed, and digested tissues transferred to 15 mL Ca²⁺/Mg²⁺-free HBSS. Liver capsules were gently disrupted to release the digested cells, which were then filtered through a 100 μm and 70 μm strainer and centrifuged at 50 × *g* for 1 min without brake. The pellet contained hepatocytes, while the supernatant contained non-parenchymal cells. For 3-month CR^tdT^ mice used for electron microscopy analysis, the supernatant also contained small tdT^+^ hepatocytes. Hepatocytes from the hepatocyte fraction were washed in Ca²⁺/Mg²⁺-free HBSS twice more, then resuspended in 30% Percoll-William’s medium and centrifuged at 150 × *g* for 5 min. The resulting hepatocyte pellet was resuspended in William’s medium, stained with 1 μg/mL DAPI, and sorted by FACS using a BD FACSAria II with a 100 μm nozzle. To isolate small tdT^+^ hepatocytes from the supernatant fraction, the supernatant from the first centrifugation step was separated and centrifuged at 1000 x g for 5 min to pellet the cells. The pellet was resuspended in Ca^2+^/Mg^2+^-free HBSS to wash the cells and centrifuged again at the same speed. The resulting pellet was resuspended at a concentration of 10 million cells per mL in 2% FBS-DMEM and sorted using a BD FACSAria II using the gating strategy shown in Supplementary Fig. 15. For DHE experiments to analyze ROS activity, hepatocytes were isolated in the same way as described above. Hepatocytes were prepared in William’s medium at 1 × 10^6^ cells/mL and treated with DHE (Abcam; ab236206) for 30 min at 37 °C. Cells were centrifuged at 100 × *g* for 5 min to remove remaining DHE, resuspended in William’s medium, and analyzed using a BD FACSymphony S6 using the gating strategy shown in Supplementary Fig. 16.

### Electron microscopy

Following FACS, cells were washed with PBS to remove the resuspension buffer and fixed with 2% paraformaldehyde (Electron Microscopy Sciences; 15710) and 2% glutaraldehyde (Electron Microscopy Sciences; 16020) in 0.1 M sodium cacodylate buffer (Electron Microscopy Sciences; 11650) for one hour, and stored at 4 °C. Samples were washed with 0.1 M sodium cacodylate buffer pH 7.4 with 2 mM calcium chloride and then fixed with freshly prepared 1.5% potassium ferrocyanide and 1% osmium tetroxide in 0.1 M cacodylate buffer pH 7.4 with 1 mM calcium chloride for 2 h at RT. Samples were then en bloc stained with 1% uranyl acetate overnight at RT, protected from light. The following day, the samples were dehydrated with a graded series of acetone and then infiltrated with EMBed-812 resin. Samples were polymerized in BEEM capsules or aluminum dishes with Embed-812 resin at 60˚C for 24–48 h. Using a Leica UC7 ultramicrotome, 60-70 nm thick sections were collected onto 200-mesh copper grids coated with a formvar/carbon film. The grids were post-stained with 1% uranyl acetate in 50% methanol and Reynolds lead citrate. Samples were imaged on a Zeiss Libra 120 TEM, and images were acquired with a Gatan Ultrascan 1000 CCD.

### Western blot

Liver tumors were excised following PBS-Heparin cardiac infusion and snap-frozen in liquid nitrogen. Frozen liver tumor tissues were homogenized on ice in RIPA buffer (Sigma; 20-188) supplemented with Halt™ protease and phosphatase inhibitor cocktail (Thermo Fisher Scientific; 78440). Lysates were incubated on ice for 15 min with intermittent vortexing, followed by centrifugation at 15,000 × *g* for 15 min at 4 °C. The supernatant was collected, and protein concentrations were determined using the Bradford assay (Thermo Fisher Scientific; 23246). 10 µg of protein were mixed with 4× Laemmli sample buffer (Bio-Rad; 1610747) with β-mercaptoethanol and heated at 95 °C for 10 min. Samples were resolved by SDS–PAGE using 4–15% gradient gels (Bio-Rad; 4564084) run at 100 V for 75 min in Tris–glycine running buffer. Proteins were then transferred onto nitrocellulose membranes at 100 V for 90 min in cold transfer buffer. Membranes were blocked for 1 h at room temperature in 5% nonfat dry milk prepared in TBS with 0.1% Tween-20 (TBST), then incubated overnight at 4 °C with primary antibodies (see Supplementary Table 1) diluted in 5% milk/TBST. After washing three times with TBST, membranes were incubated with the appropriate HRP-conjugated secondary antibody for 1 h at room temperature. Following additional washes, bands were visualized using SuperSignal™ West Dura Extended Duration Substrate (Thermo Fisher Scientific; 34075), and chemiluminescence was detected by exposure to X-ray film (VWR; 103254-906). Densitometry analysis was performed with ImageJ using the Gel Analyzer tool.

### DNA Isolation and PCR Genotyping

DNA was isolated from frozen liver tissues according to the DNeasy Blood & Tissue Kit (Qiagen; 69504) manufacturer’s protocol. PCR reactions for detection of *Nfe2l2* recombination was done using the following primers synthesized by IDT: N1 Forward (TCTTAGGCACCATTTGGGAGAG), N2 Reverse (TACAGCAGGCATACCATTGTGG), and N3 Forward (TGAGAGCTTCCCAGACTCACTT)^52^. The reaction was performed using 12.5 µl of GoTaq G2 Master Mix (Promega; M7123), 0.5 µl of each primer, 150 ng of DNA, brought to 25 µl with nuclease free water, and amplified using a T100 Thermal Cycler (Bio-Rad). The reactions were performed at 95 °C for 3 min, followed by 33 cycles of 95 °C for 30 s, 58 °C for 30 s, and 72 °C for 45 s, and finally at 72 °C for 5 min. PCR products were run on a 1.5% (w/v) agarose gel in 1x TAE buffer with a 1 Kb Plus DNA Ladder (ThermoFisher Scientific; 10787018) and imaged using a Gel Doc EZ Imager (Bio-Rad).

### Single-cell RNA sequencing and analysis

Single-cell RNA-seq was performed using the Chromium Next GEM Single Cell 3′ GEM, Library & Gel Bead Kit v3.1 (10x Genomics, PN-1000121). Following FACS, cells were counted on a Zeiss Axio Observer and assessed for viability (>85% live) and singlet fraction. Sorted populations were pooled at a 1:1 ratio of tdT⁻ and tdT⁺ cells and loaded into a single well of a Next GEM Chip G. Cell partitioning into Gel Beads-in-emulsions (GEMs) and barcoding were carried out using the Chromium Controller, following the manufacturer’s protocol. Barcoded cDNA libraries were prepared from GEMs for each mouse, and sequencing was performed on an Illumina HiSeq X platform (PE150).

Raw sequencing data files were processed using Cell Ranger v.8.0.1 from 10x Genomics, and reads were mapped to the GRCm39 reference genome modified to include the tdTomato sequence found in R^tdT^ mice. All other parameters were kept as default. The resultant data was then processed using the Seurat 5.0.2 package in R. Cells were initially filtered out if they did not meet the QC criteria (n_feature count between 400 and 7000 and mitochondrial genes <20% for the normal liver samples and 950–7000, <20% for the CLD samples). Cells that passed QC were then visualized by mapping to uniform manifold approximation and projection (UMAP) dimensionality reduction plots using standard Seurat parameters. Temporal and hepatocytes zonation markers were used to identify clusters, and resultant non-hepatocytes (endothelial cells, immune cells, etc.) were removed. Integration of the replicates was performed with the RPCA method. Furthermore, clusters defined by cells that displayed low total gene and transcript counts were removed. Cluster-specific profiles were analyzed using standard differential signaling pathway analysis with Hallmark gene set collection “H” and Reactome C2:CP gene collection. Benjamini–Hochberg was used to adjust the *p*-values, and 0.05 was set as the threshold for enrichment significance. Zone 3 hepatocyte signature was built with the top 50 genes of CV vs MZ from a previously published study^53^. All gene signature expression analyses were based on complete gene entries in the given Hallmark or Reactome signature, except for the pan-hepatocyte signature: Alb, Ttr, Apob, Apoa1, Rbp4, Hnf4a, Cebpa, Asgr1, Asgr2, Fabp, and the proliferation signature: Mki67, Pcna, Cdk1, Mcm2, Mcm3, Mcm4, Mcm5, Mcm6, Mcm7, Ccnb1, Aurkb, Cenpf, Top2a. Glutathione Synthesis: Gclc, Gclm, Gss. Glutathione use and recycle: Gsr, Gpx1, Gpx2, Gpx3, Gpx4. Glutathione conjugation: Gsta1, Gsta2, Gstm1, Gstm2, Gstm3, Gstm4, Gstp1, Gstp2, Mgst1, Mgst3.

### RNA sequencing and analysis

RNA was extracted from excised 6.5-month CMR^tdT^ liver tumors using the AllPrep DNA/RNA Mini Kit (Qiagen; 80204) according to the manufacturer’s protocol. Samples mRNA libraries were prepared by Novogene with poly A enrichment and sequenced using a NovaSeq 6000 (PE 150).

Analysis of expression correlations was performed by integrating the new tumors’ expression results with a previously established analysis of pairwise correlations between human HCCs and mouse liver tumors^28^. Briefly, RNA-seq transcript levels were quantified as transcripts per million (TPM), and only 1:1 mouse–human orthologs were retained for analysis. Differential expression analysis was performed using DESeq2 on TCGA hepatocellular carcinoma (HCC) tumors and tissue-matched normal samples to identify genes associated with HCC and with distinct molecular subtypes defined by Broad Firehose clustering. Genes with >2-fold change and FDR < 0.05 were considered significantly differentially expressed, resulting in a set of 800 HCC-associated genes.

For cross-species comparison, TPM values for these genes were log2 transformed, z-score normalized, and used to calculate Pearson correlation–based similarities between human and mouse tumors. Unsupervised hierarchical clustering was then performed using Euclidean distance and average linkage to define major and minor human HCC subgroups.

### Whole exome sequencing and analysis

DNA was isolated from frozen liver tissues, two tumors and surrounding non-tumor bearing from five independent mice, according to the AllPrep DNA/RNA Mini Kit (Qiagen; 80204) manufacturer’s protocol. The genomic DNA was randomly sheared into short fragments with the size of 180-280 bp. The obtained fragments were end repaired, A-tailed, and further ligated with Illumina adapters. Hybridization capture of libraries was proceeded according to the following procedures. Briefly, the prepped libraries were hybridized in the buffer with biotin-labeled probes, and magnetic beads with streptavidin were used to capture the exons of genes. Subsequently, non-hybridized fragments were washed out and probes were digested. The captured libraries were enriched by PCR amplification, and sequencing was performed using a NovaSeq X Plus (PE 150).

For data analysis, reads were filtered out that contained adaptors or with low quality using fastp (v0.23.1). The reads were then aligned to the reference mm10 mouse genome using Burrows-Wheeler Aligner (v0.7.17), Sambamba (v1.0.0), and Picard (v2.18.9). SNP/InDel calling, annotation, and statistics were performed using GATK (v.4.3.0) and Somatic SNP/InDel calling was performed using MuTect2 (v4.3.0). Somatic CNV detection was performed using Control-FREEC (v.11.4).

Excluding the outlier tumor Mouse 2_T1, the remaining *Ctnnb1*-driven tumors displayed a low overall somatic SNV/indel burden and a sparse coding mutational landscape. Across the 9 tumors, the median number of total somatic calls was 106, with a median of 11 coding/splice variants and 9 protein-altering variants per tumor. MuTect2 somatic variant calls identified no recurrent protein-altering mutation pattern across independent mice and no obvious classical oncogenic or tumor suppressor SNV/indel driver landscape beyond engineered Ctnnb1 activation (Supplementary Fig. 4). The only recurrent coding event was an H3c2 missense variant shared between the two tumors derived from Mouse 4, without recurrence across independent animals. Recurrent shared variants were otherwise predominantly noncoding/intergenic and were consistent with background technical or alignment-associated signal rather than recurrent biological driver events.

One tumor, Mouse 2_T1, showed a marked outlier profile with 2427 total somatic SNV/indel calls, including 744 coding/splice and 555 protein-altering variants. However, focused review did not identify an obvious classical oncogenic driver or a clear DNA repair, polymerase, or mismatch-repair alteration explaining a bona fide hypermutator phenotype. The tumor also demonstrated an unusually low median VAF (median 5-6%) and a very low Ti/Tv ratio (0.224), indicating the callset is dominated by technical artifacts rather than biological mutations. Accordingly, Mouse 2_T1 was excluded from the primary cohort-level interpretation.

No SNV/indel was detected in any tumor for the somatic coding or splice-region of *Nfe2l2*. This conclusion is supported both by MuTect2 somatic variant calls and by independent BAM-level pileup inspection of *Nfe2l2* coding/splice target regions, which found no convincing tumor-specific alternate allele support at either strict or exploratory thresholds confirming MuTect2 results.

### Image analysis

All immunofluorescence images were analyzed manually by NDP.view2 or ImageJ. Sample information was not blinded for the analyzer. Clone counts were quantified by drawing pre-defined regions in each lobe of the liver and marking cells positive for tdTomato and/or GS signal. Areas varied from 1 mm^2^ to the entire lobe to achieve a sufficient number of cells counted and avoid bias due to cell density. For zonation clone counts, GS in addition to E-Cadherin were used to mark the pericentral and periportal zones, respectively. Pre-defined regions of each lobe were drawn, and further subdivided into zones 1, 2, and 3. Double positive tdTomato and GS clones were quantified per zone area. For all cell size quantifications, pre-defined areas were drawn in each lobe, and only cells with visible DAPI signal were traced. Approximately 150–200 cells of each type defined in the analysis were counted per mouse. For all clone size quantifications, pre-defined areas were drawn in each lobe, and clones were traced such that cells with the same staining pattern in physical contact were treated as part of the same clone. Approximately 150–200 clones of each type defined in the analysis were counted in each mouse. Entire lobes were quantified in scenarios where clones were very large to avoid bias. Serial sections with at least 40 μm gaps between sections were quantified when insufficient cells were counted in one slide. Areas varied from 1 mm^2^ to the entire lobe to achieve a sufficient number of cells counted and avoid bias due to cell density and tissue location. For CHOP, BiP, and Ki67 quantifications, predefined areas in each lobe were marked, and each tdT^+^ cell was labelled based on its staining pattern for GS and CHOP, BiP, or Ki67. As CHOP and Ki67 are localized to the nucleus, only DAPI-positive cells were quantified in CHOP and Ki67 stained samples. At least 100 cells were quantified per lobe. For analysis of the frequency of NQO1 positive clones in the CNMR mice, double positive tdTomato and GS clones were traced, denoted as either NQO1 positive or negative, and separated into sextiles based on clone area. Only clones 5 cells or more were counted.

### Statistical analysis and reproducibility

Source data are provided as a Source Data file. Data were processed and visualized in GraphPad Prism v.8.4.3 and R v.4.2.1. Each statistical test performed is detailed in the corresponding figure legends. In summary, an unpaired t-test was performed when two independent groups are compared, and a paired t-test was performed when comparing cell populations from the same mouse. Data were log(10) transformed to improve normality and stabilize variance for statistical analyses. Cell size analyses were performed using linear mixed-effects models fitted to log-transformed measurements. Models included Population (i.e. GS+ vs GS − ) as a fixed effect and mouse replicates as a random effect with random slopes. Mixed models were fitted with the nlme package, and the emmeans package was used for post-hoc contrasts. Estimated marginal means and contrasts were back-transformed to the original measurement scale and reported as ratios with 95% confidence intervals (CIs). Percent changes and 95% CIs are provided for analyses with a p-value less than 0.1.

For clone size analyses in Fig. 2C,F, thresholds to distinguish single- vs. multi-cellular clones for each mouse were established empirically by determining the area distribution of individual *Ctnnb1*^*ex3*^ hepatocytes. The same mixed-effects analysis was then applied independently to single-cell and multicellular clones to test differences in phenotype above or below the threshold in CMR^tdT^ or aged CR^tdT^ mice within each population. The average threshold across mice for each genotype was plotted for each timepoint as a visual reference.

*n* numbers refer to number of mice for all experiments and are described in figure legends. All experiments were replicated at least 3 times, except for select scRNA-seq data described in their respective figure legends. Sample sizes were determined based on our previous experience with the models and published studies in the field. No statistics were used to predetermine the sample size. All mice were randomly assigned to experimental groups.

### Reporting summary

Further information on research design is available in the Nature Portfolio Reporting Summary linked to this article.

## Supplementary information

Supplementary Information

Reporting Summary

Transparent Peer Review file

## Source data

Source Data

## Data Availability

RNA sequencing data have been deposited in the GEO database under accession codes GSE306578 and GSE334406. Raw exome sequencing data have been deposited in the SRA database under accession code PRJNA1465411. The remaining data are available within the Article, Supplementary Information or Source Data file. Source data are provided with this paper.

## References

[1] Blokzijl, F. et al. Tissue-specific mutation accumulation in human adult stem cells during life. *Nature* **538**, 260–264 (2016). 27698416 10.1038/nature19768 PMC5536223

[2] Ng, S. W. K. et al. Convergent somatic mutations in metabolism genes in chronic liver disease. *Nature* **598**, 473–478 (2021). 34646017 10.1038/s41586-021-03974-6

[3] Zhu, M. et al. Somatic mutations increase hepatic clonal fitness and regeneration in chronic liver disease. *Cell* **177**, 608–621 e612 (2019). 30955891 10.1016/j.cell.2019.03.026 PMC6519461

[4] Surveillance, E., and End Results (SEER) Program Populations (1969-2020) *(*www.seer.cancer.gov/popdata*)*, *National Cancer Institute, DCCPS, Surveillance Research Program, released**2022*.

[5] Jaiswal, S. & Ebert, B. L. Clonal hematopoiesis in human aging and disease. *Science* **366** 10.1126/science.aan4673 (2019). PMC8050831 31672865

[6] Martincorena, I. Seeds of cancer in normal skin. *Nature* **586**, 504–506 (2020). 33028991 10.1038/d41586-020-02749-9

[7] Martincorena, I. et al. Somatic mutant clones colonize the human esophagus with age. *Science* **362**, 911–917 (2018). 30337457 10.1126/science.aau3879 PMC6298579

[8] Marusyk, A. & DeGregori, J. Declining cellular fitness with age promotes cancer initiation by selecting for adaptive oncogenic mutations. *Biochim Biophys. Acta* **1785**, 1–11 (2008). 17980163 10.1016/j.bbcan.2007.09.001 PMC2234267

[9] Cancer Genome Atlas Research Network. Electronic address, w. b. e. & Cancer Genome Atlas Research, N. Comprehensive and Integrative Genomic Characterization of Hepatocellular Carcinoma. *Cell* **169**, 1327-1341 e1323 (2017). 10.1016/j.cell.2017.05.046 PMC5680778 28622513

[10] Harada, N. et al. Lack of tumorigenesis in the mouse liver after adenovirus-mediated expression of a dominant stable mutant of beta-catenin. *Cancer Res* **62**, 1971–1977 (2002). 11929813

[11] Rebouissou, S. et al. Genotype-phenotype correlation of CTNNB1 mutations reveals different ss-catenin activity associated with liver tumor progression. *Hepatology* **64**, 2047–2061 (2016). 27177928 10.1002/hep.28638

[12] Lemberger, U. J. et al. Hepatocyte specific expression of an oncogenic variant of beta-catenin results in cholestatic liver disease. *Oncotarget* **7**, 86985–86998 (2016). 27895309 10.18632/oncotarget.13521 PMC5349966

[13] Nejak-Bowen, K. N. et al. Accelerated liver regeneration and hepatocarcinogenesis in mice overexpressing serine-45 mutant beta-catenin. *Hepatology* **51**, 1603–1613 (2010). 20432254 10.1002/hep.23538 PMC2908905

[14] Tward, A. D. et al. Distinct pathways of genomic progression to benign and malignant tumors of the liver. *Proc. Natl. Acad. Sci. USA*. **104**, 14771–14776 (2007). 17785413 10.1073/pnas.0706578104 PMC1964540

[15] Tao, J. et al. Modeling a human hepatocellular carcinoma subset in mice through coexpression of met and point-mutant beta-catenin. *Hepatology* **64**, 1587–1605 (2016). 27097116 10.1002/hep.28601 PMC5073058

[16] Monga, S. P. β-catenin signaling and roles in liver homeostasis, injury, and tumorigenesis. *Gastroenterology* **148**, 1294–1310 (2015). 25747274 10.1053/j.gastro.2015.02.056 PMC4494085

[17] Xu, C. et al. β-Catenin signaling in hepatocellular carcinoma. *J. Clin. Investig.* **132** 10.1172/JCI154515 (2022). PMC8843739 35166233

[18] Harada, N. et al. Hepatocarcinogenesis in mice with beta-catenin and Ha-ras gene mutations. *Cancer Res* **64**, 48–54 (2004). 14729607 10.1158/0008-5472.can-03-2123

[19] Guo, J. et al. The origin of hepatocellular carcinoma depends on metabolic zonation. *Science* **391**, eadv7129 (2025). 10.1126/science.adv7129 PMC12999168 41196951

[20] Raven, A. et al. Hepatic zonation determines tumorigenic potential of mutant beta-catenin. *Nature* **649**, 739–748 (2025). 41261129 10.1038/s41586-025-09733-1 PMC12804091

[21] Madisen, L. et al. A robust and high-throughput Cre reporting and characterization system for the whole mouse brain. *Nat. Neurosci.* **13**, 133–140 (2010). 20023653 10.1038/nn.2467 PMC2840225

[22] Srivastava, A. In vivo tissue-tropism of adeno-associated viral vectors. *Curr. Opin. Virol.* **21**, 75–80 (2016). 27596608 10.1016/j.coviro.2016.08.003 PMC5138125

[23] Kiourtis, C. et al. Specificity and off-target effects of AAV8-TBG viral vectors for the manipulation of hepatocellular gene expression in mice. *Biol. Open* **10**, bio058678 (2021). 34435198 10.1242/bio.058678 PMC8487635

[24] Malato, Y. et al. Fate tracing of mature hepatocytes in mouse liver homeostasis and regeneration. *J. Clin. Investig.* **121**, 4850–4860 (2011). 22105172 10.1172/JCI59261 PMC3226005

[25] Sekine, S., Lan, B. Y., Bedolli, M., Feng, S. & Hebrok, M. Liver-specific loss of beta-catenin blocks glutamine synthesis pathway activity and cytochrome p450 expression in mice. *Hepatology* **43**, 817–825 (2006). 16557553 10.1002/hep.21131

[26] Tan, X., Behari, J., Cieply, B., Michalopoulos, G. K. & Monga, S. P. Conditional deletion of beta-catenin reveals its role in liver growth and regeneration. *Gastroenterology* **131**, 1561–1572 (2006). 17101329 10.1053/j.gastro.2006.08.042

[27] Chafey, P. et al. Proteomic analysis of beta-catenin activation in mouse liver by DIGE analysis identifies glucose metabolism as a new target of the Wnt pathway. *Proteomics* **9**, 3889–3900 (2009). 19639598 10.1002/pmic.200800609

[28] Dow, M. et al. Integrative genomic analysis of mouse and human hepatocellular carcinoma. *Proc. Natl. Acad. Sci. USA*. **115**, E9879–E9888 (2018). 30287485 10.1073/pnas.1811029115 PMC6196518

[29] Font-Burgada, J. et al. Hybrid periportal hepatocytes regenerate the injured liver without giving rise to cancer. *Cell* **162**, 766–779 (2015). 26276631 10.1016/j.cell.2015.07.026 PMC4545590

[30] Nakagawa, H. et al. ER stress cooperates with hypernutrition to trigger TNF-dependent spontaneous HCC development. *Cancer Cell* **26**, 331–343 (2014). 25132496 10.1016/j.ccr.2014.07.001 PMC4165611

[31] Strathearn, L. S. et al. Plastic hepatocyte states limit liver cancer development. *Nat. Commun.* **16**, 11647 (2025). 41290681 10.1038/s41467-025-66568-0 PMC12748961

[32] Chen, Z., Tian, R., She, Z., Cai, J. & Li, H. Role of oxidative stress in the pathogenesis of nonalcoholic fatty liver disease. *Free Radic. Biol. Med.* **152**, 116–141 (2020). 32156524 10.1016/j.freeradbiomed.2020.02.025

[33] Menon, S. et al. Chronic activation of mTOR complex 1 is sufficient to cause hepatocellular carcinoma in mice. *Sci. Signal* **5**, ra24 (2012). 22457330 10.1126/scisignal.2002739 PMC3743103

[34] Kato, H. et al. mTORC1 serves ER stress-triggered apoptosis via selective activation of the IRE1-JNK pathway. *Cell Death Differ.* **19**, 310–320 (2012). 21779001 10.1038/cdd.2011.98 PMC3263505

[35] Walsh, J. et al. Identification and quantification of the basal and inducible Nrf2-dependent proteomes in mouse liver: biochemical, pharmacological and toxicological implications. *J. Proteom.* **108**, 171–187 (2014). 10.1016/j.jprot.2014.05.007 PMC4115266 24859727

[36] Shelton, L. M. et al. Integrated transcriptomic and proteomic analyses uncover regulatory roles of Nrf2 in the kidney. *Kidney Int.* **88**, 1261–1273 (2015). 26422507 10.1038/ki.2015.286 PMC4676083

[37] Singh, S., Singh, P. P., Roberts, L. R. & Sanchez, W. Chemopreventive strategies in hepatocellular carcinoma. *Nat. Rev. Gastroenterol. Hepatol.* **11**, 45–54 (2014). 23938452 10.1038/nrgastro.2013.143 PMC4334449

[38] Tao, J. et al. Nuclear factor erythroid 2-related factor 2 and beta-Catenin Coactivation in Hepatocellular Cancer: Biological and Therapeutic Implications. *Hepatology* **74**, 741–759 (2021). 33529367 10.1002/hep.31730 PMC8326305

[39] Stauffer, J. K. et al. Coactivation of AKT and beta-catenin in mice rapidly induces formation of lipogenic liver tumors. *Cancer Res.* **71**, 2718–2727 (2011). 21324921 10.1158/0008-5472.CAN-10-2705 PMC3074499

[40] He, F. et al. NRF2 activates growth factor genes and downstream AKT signaling to induce mouse and human hepatomegaly. *J. Hepatol.* **72**, 1182–1195 (2020). 32105670 10.1016/j.jhep.2020.01.023 PMC8054878

[41] Yecies, J. L. et al. Akt stimulates hepatic SREBP1c and lipogenesis through parallel mTORC1-dependent and independent pathways. *Cell Metab.* **14**, 21–32 (2011). 21723501 10.1016/j.cmet.2011.06.002 PMC3652544

[42] Du, K., Herzig, S., Kulkarni, R. N. & Montminy, M. TRB3: a tribbles homolog that inhibits Akt/PKB activation by insulin in liver. *Science* **300**, 1574–1577 (2003). 12791994 10.1126/science.1079817

[43] Villanueva, A. et al. Pivotal role of mTOR signaling in hepatocellular carcinoma. *Gastroenterology* **135**, 1972-1983, 1983 e1971-1911 (2008). 10.1053/j.gastro.2008.08.008 PMC2678688 18929564

[44] Ono, H. et al. Hepatic Akt activation induces marked hypoglycemia, hepatomegaly, and hypertriglyceridemia with sterol regulatory element binding protein involvement. *Diabetes* **52**, 2905–2913 (2003). 14633850 10.2337/diabetes.52.12.2905

[45] Calvisi, D. F. et al. Increased lipogenesis, induced by AKT-mTORC1-RPS6 signaling, promotes development of human hepatocellular carcinoma. *Gastroenterology* **140**, 1071–1083 (2011). 21147110 10.1053/j.gastro.2010.12.006 PMC3057329

[46] Liu, F. et al. Oncogenic beta-catenin stimulation of AKT2-CAD-mediated pyrimidine synthesis is targetable vulnerability in liver cancer. *Proc. Natl. Acad. Sci. USA* **119**, e2202157119 (2022). 36122209 10.1073/pnas.2202157119 PMC9522414

[47] Loesch, R. et al. Deleting the beta-catenin degradation domain in mouse hepatocytes drives hepatocellular carcinoma or hepatoblastoma-like tumor growth. *J. Hepatol.* **77**, 424–435 (2022). 35257829 10.1016/j.jhep.2022.02.023

[48] Brunner, S. F. et al. Somatic mutations and clonal dynamics in healthy and cirrhotic human liver. *Nature* **574**, 538–542 (2019). 31645727 10.1038/s41586-019-1670-9 PMC6837891

[49] Savall, M. et al. Cooperation between the NRF2 pathway and oncogenic β-catenin during HCC tumorigenesis. *Hepatol. Commun.* **5**, 1490–1506 (2021). 34510835 10.1002/hep4.1746 PMC8435276

[50] Harada, N. et al. Intestinal polyposis in mice with a dominant stable mutation of the beta-catenin gene. *EMBO J*. **18**, 5931–5942 (1999). 10.1093/emboj/18.21.5931 PMC1171659 10545105

[51] Weglarz, T. C., Degen, J. L. & Sandgren, E. P. Hepatocyte transplantation into diseased mouse liver. Kinetics of parenchymal repopulation and identification of the proliferative capacity of tetraploid and octaploid hepatocytes. *Am. J. Pathol.* **157**, 1963–1974 (2000). 10.1016/S0002-9440(10)64835-3 PMC1885759 11106569

[52] Reddy, N. M., Potteti, H. R., Mariani, T. J., Biswal, S. & Reddy, S. P. Conditional deletion of Nrf2 in airway epithelium exacerbates acute lung injury and impairs the resolution of inflammation. *Am. J. Respir. Cell. Mol. Biol.* **45**, 1161–1168 (2011). 10.1165/rcmb.2011-0144OC PMC3262666 21659655

[53] Xu, J. et al. A spatiotemporal atlas of mouse liver homeostasis and regeneration. *Nat. Genet*. **56**, 953–969 (2024). 10.1038/s41588-024-01709-7 38627598

