# Supplementary material for: Adaptive oncogenesis of β-catenin emerges from declining liver tissue integrity
Source: Nat Commun. 2026 Aug 10;17:9565. doi: 10.1038/s41467-026-76373-y (PMC13550535; doi:10.1038/s41467-026-76373-y)
Supplement: Supplementary file 1 [file 41467_2026_76373_MOESM1_ESM.pdf]

Antibody	Vendor	Catalog Number	Dilution
**Goat anti-mCherry (tdTomato)**	Sicgen	AB0040-200	1:1500 (R^{tdT} Samples)<br>1:500 (Transposons)
**Mouse anti-GS**	BD	610517	1:200
**Rabbit anti-GFP (YFP)**	Invitrogen	A-11122	1:100
**Chicken anti-GFP (YFP)**	Novus	NB100-1614	1:100
**Mouse anti-CHOP**	CST	2895	1:100 with TSA kit
**Rabbit anti-BiP**	CST	3177	1:100
**Rabbit anti-NQO1**	Sigma	HPA007308	1:100
**Goat anti-E-Cadherin**	R&D Systems	AF748	1:100
**Rabbit anti-P-Akt (Ser473)**	CST	4060S	1:100
**Rabbit anti-P-Akt (Thr308)**	CST	4056S	1:100
**Rabbit anti-Phospho-PRAS40 (Thr246)**	CST	2997S	1:100
**Rabbit anti-Fatty Acid Synthase**	CST	3180S	1:100
**Rabbit anti-SCD1**	CST	2794S	1:100
**Rabbit anti-HMGCS1**	Proteintech	17643-1-AP	1:100
**Rabbit anti-Ki67**	Abcam	ab15580	1:500
**Donkey anti-mouse 488**	Invitrogen	A21202	1:600
**Donkey anti-goat 555**	Invitrogen	A21432	1:600
**Donkey anti-goat 647**	Invitrogen	A21447	1:600
**Donkey anti-goat 755**	Invitrogen	SA5-10091	1:600
**Donkey anti-rabbit 555**	Invitrogen	A31572	1:600
**Donkey anti-rabbit 647**	Invitrogen	A31573	1:600
**Donkey anti-chicken 555**	Jackson ImmunoResearch	703-165-155	1:600
**Goat Anti-Mouse IgG (Biotin conjugated)**	Vector Laboratories	BA-9200	1:200 with TSA kit
**Rabbit anti-NRF2**	GeneTex	GTX103322	1:1000
**Rabbit anti-GAPDH**	CST	2118	1:5000
**Anti-rabbit IgG (HRP-linked)**	CST	7074	1:2500 (NRF2)<br>1:5000 (GAPDH)

Supplementary Table 1. Antibodies Used for Experiments

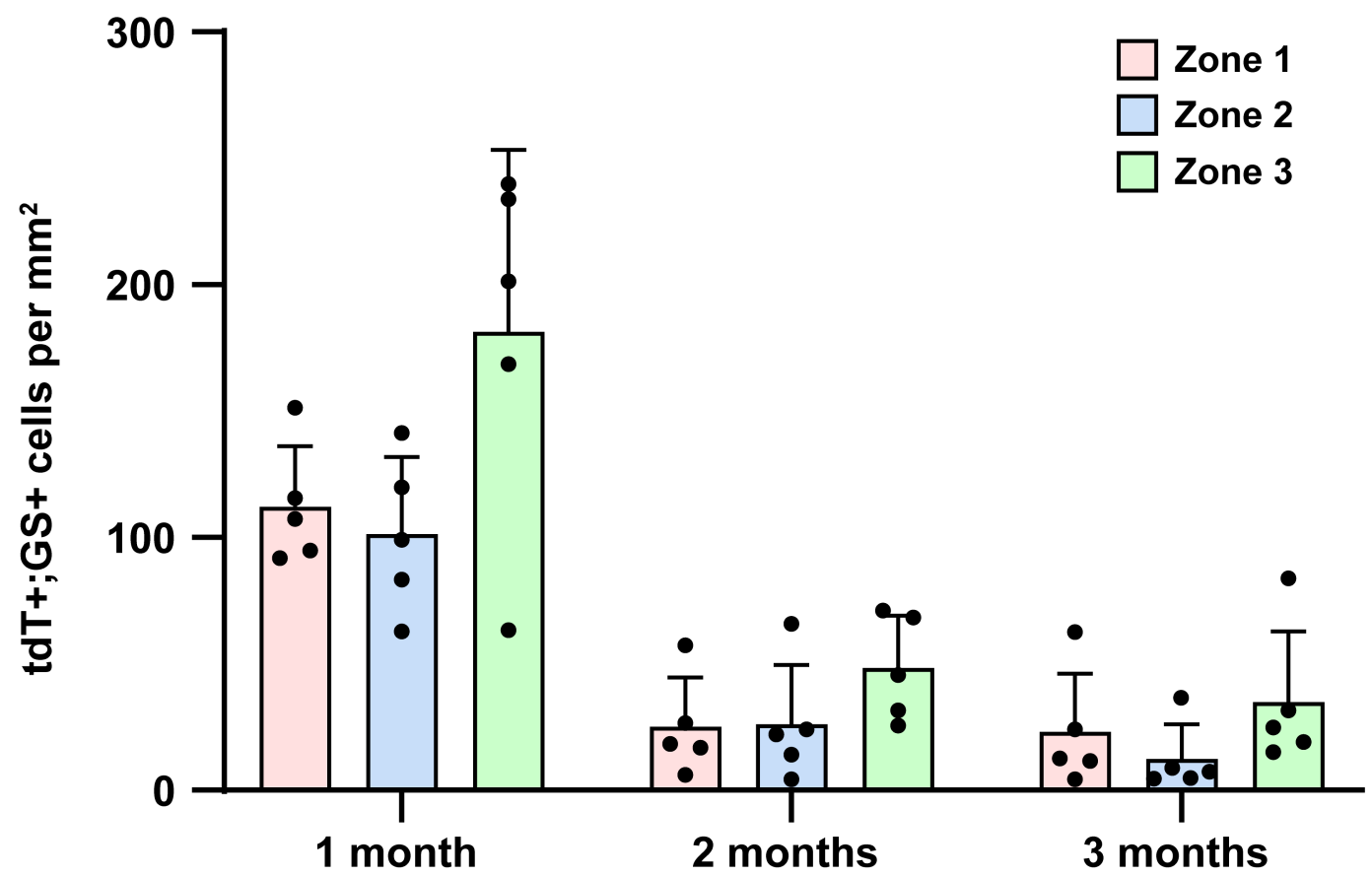

Supplementary Figure 1. β-catenin–mutant hepatocyte attrition is not affected by liver zonation.

Quantification of the number of tdT^{+};GS^{+} hepatocytes per mm^2 within either Zone 1 (periportal; E-$Cadherin^{+}$), Zone 2 (E-$Cadherin^{-}$;GS^{-}), or Zone 3 (pericentral; GS^{+}) at the indicated timepoints post-AAV injection. Each point represents the mean count from a mouse. Each bar represents the average of the mice means +SD. n=5 for each timepoint. Source data are provided as a Source Data file.

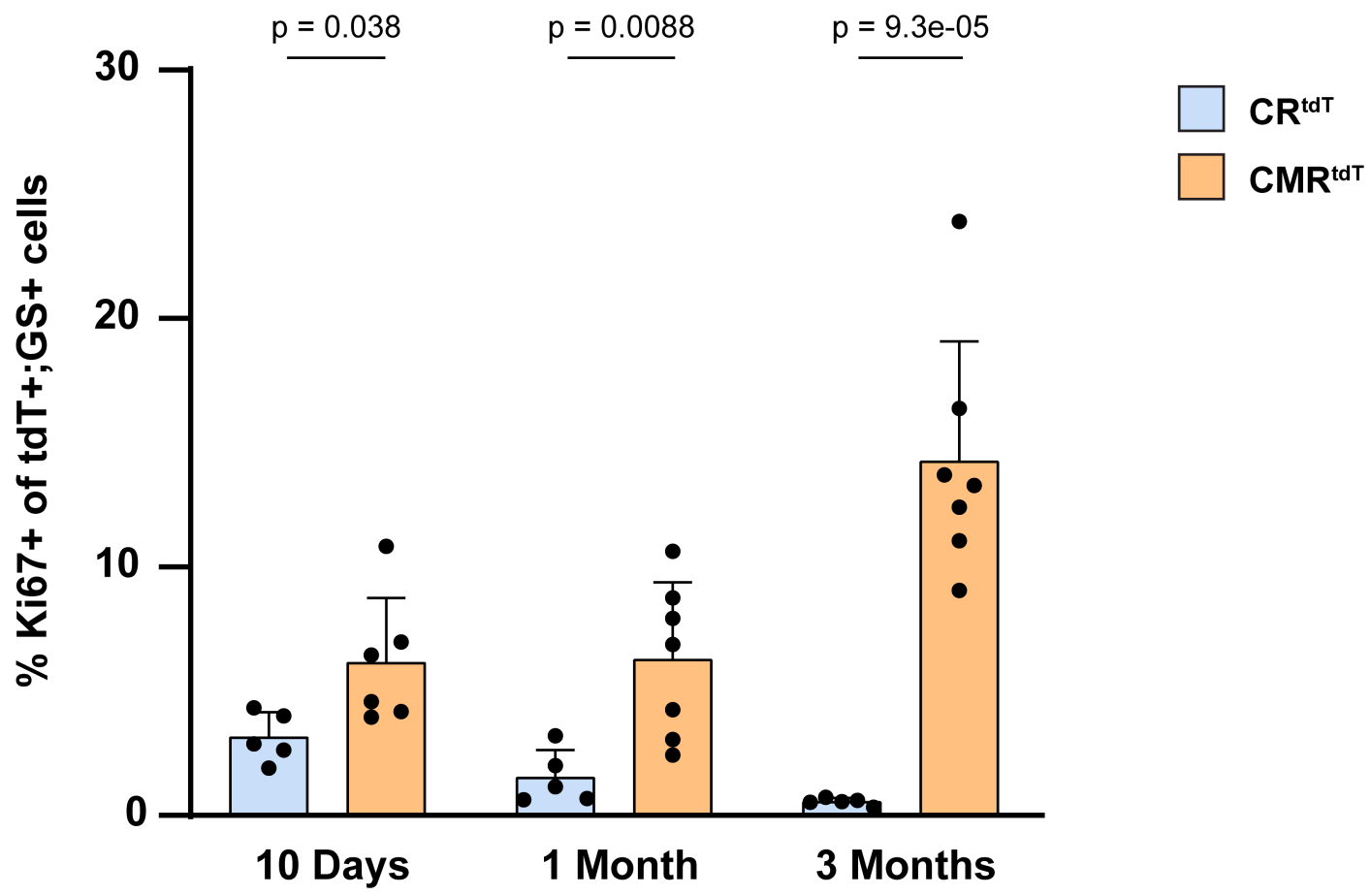

Supplementary Figure 2. ***Ctnnb1**^{ex3}* **hepatocytes proliferate in the damaged liver but not in the normal liver.**

%tdT^{+};GS^{+} cells that are also Ki67^{+} from same samples described in (Fig. 1C and 2C) at the indicated timepoints. Each point represents the mean count from a mouse. Each bar represents the average of the mice means +SD. Statistical significance was determined between CR^{tdT} and CMR^{tdT} groups using an unpaired, two-tailed t-test using the mice means. n=5 for CR^{tdT} at each timepoint. n=6, 7, 7 for 10 days, 1 month, and 3 months CMR^{tdT}, respectively. Source data are provided as a Source Data file.

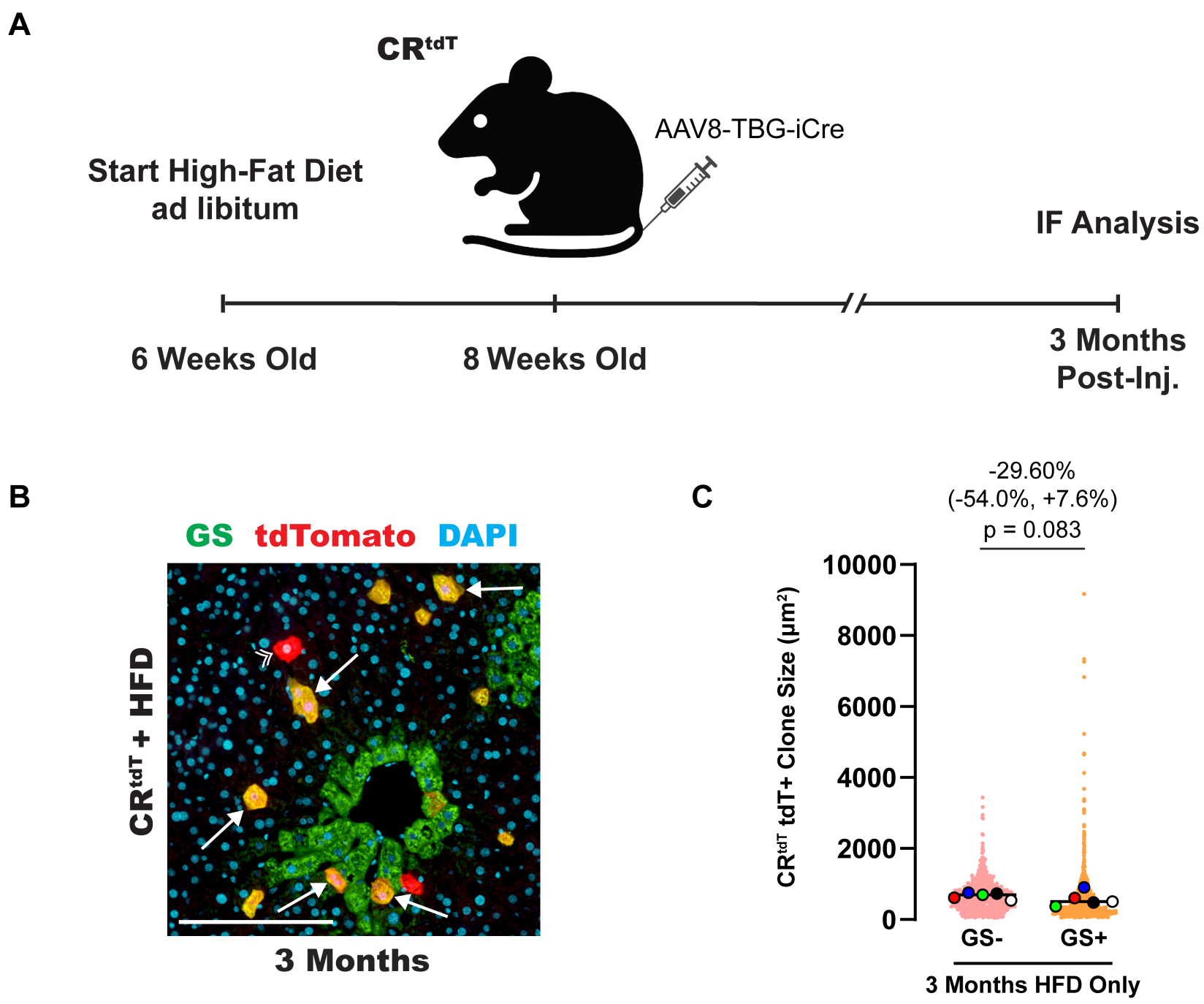

Supplementary Figure 3. *Ctnnb1*^{ex3} hepatocytes do not clonally expand with High-Fat Diet alone at 3 months.

A) Schematic of High-Fat Diet treatment in CR^{tdT} mice experimental design. B) Representative immunofluorescence images of CR^{tdT} High-Fat Diet treated mice 3 months after injection stained for tdT and GS. White arrows: tdT^{+};GS^{+} clones, double arrowheads: tdT^{+} only cells. Scale bar=200μm. C) Quantification of tdT^{+};GS^{-} (GS^{-}) and tdT^{+};GS^{+} (GS^{+}) clone sizes in CR^{tdT} High-Fat Diet treated liver samples. Points are individual clones; colored dots are mouse means; black lines indicate the mean of mouse means. Same color indicates same mouse. Statistical significance between GS^{-} and GS^{+} clone size in each class was determined by fitting a linear mixed effects model with a likelihood-ratio test. Significance of pairwise comparisons was determined using post-hoc comparisons from the fitted mixed-effects model, with two-sided tests. Percentage change (top), 95% CI (middle), and p-value (bottom) of GS^{+} vs. GS^{-} cell size is listed above. n=5. Panel A was created in BioRender. Hewett, C. (2026) https://BioRender.com/t3qttns. Source data are provided as a Source Data file.

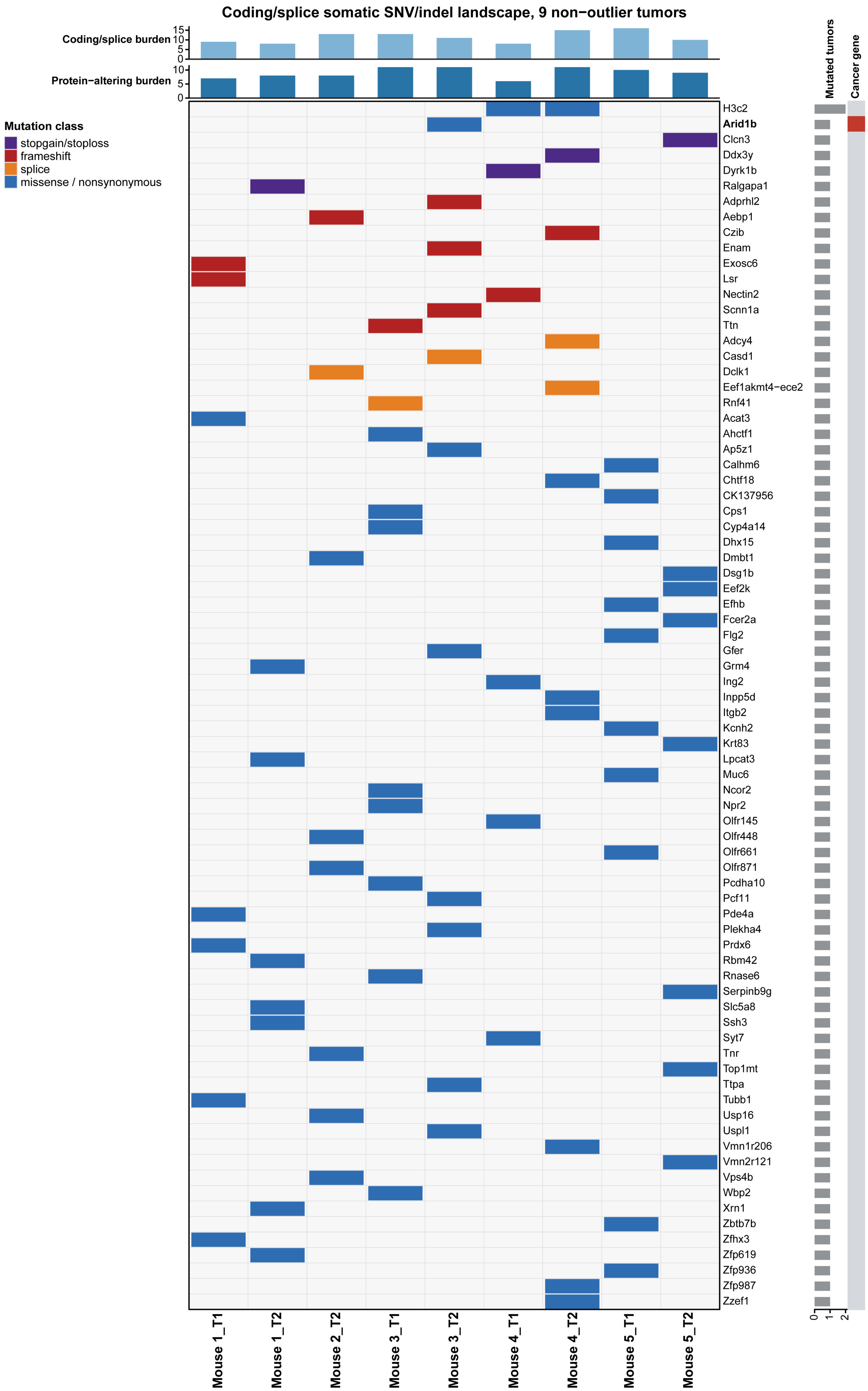
Coding/splice somatic SNV/indel landscape, 9 non−outlier tumors
Coding/splice burden
Protein−altering burden
Mutation class
stopgain/stoploss
frameshift
splice
missense / nonsynonymous
Mutated tumors
Cancer gene
H3c2
Arid1b
Clcn3
Ddx3y
Dyrk1b
Ralgapa1
Adprhl2
Aebp1
Czib
Enam
Exosc6
Lsr
Nectin2
Scnn1a
Ttn
Adcy4
Casd1
Dclk1
Eef1akmt4−ece2
Rnf41
Acat3
Ahctf1
Ap5z1
Calhm6
Chtf18
CK137956
Cps1
Cyp4a14
Dhx15
Dmbt1
Dsg1b
Eef2k
Efhb
Fcer2a
Flg2
Gfer
Grm4
Ing2
Inpp5d
Itgb2
Kcnh2
Krt83
Lpcat3
Muc6
Ncor2
Npr2
Olfr145
Olfr448
Olfr661
Olfr871
Pcdha10
Pcf11
Pde4a
Plekha4
Prdx6
Rbm42
Rnase6
Serpinb9g
Slc5a8
Ssh3
Syt7
Tnr
Top1mt
Ttpa
Tubb1
Usp16
Uspl1
Vmn1r206
Vmn2r121
Vps4b
Wbp2
Xrn1
Zbtb7b
Zfhx3
Zfp619
Zfp936
Zfp987
Zzef1
Mouse 1_T1
Mouse 1_T2
Mouse 2_T2
Mouse 3_T1
Mouse 3_T2
Mouse 4_T1
Mouse 4_T2
Mouse 5_T1
Mouse 5_T2

Supplementary Figure 4. No recurrent cooperation with additional oncogenic mutations on CMR^{tdT} tumors.

Whole-exome sequencing was performed on tumors isolated from 6.5-month CMR^{tdT} mice. Two tumors (T1 and T2) were sequenced in five independent mice, and variants were identified by comparing with matched surrounding non-tumor liver tissue from the same mouse. Mouse 2_T1 showed a marked outlier profile and was excluded from the primary cohort-level interpretation, further described in Methods.

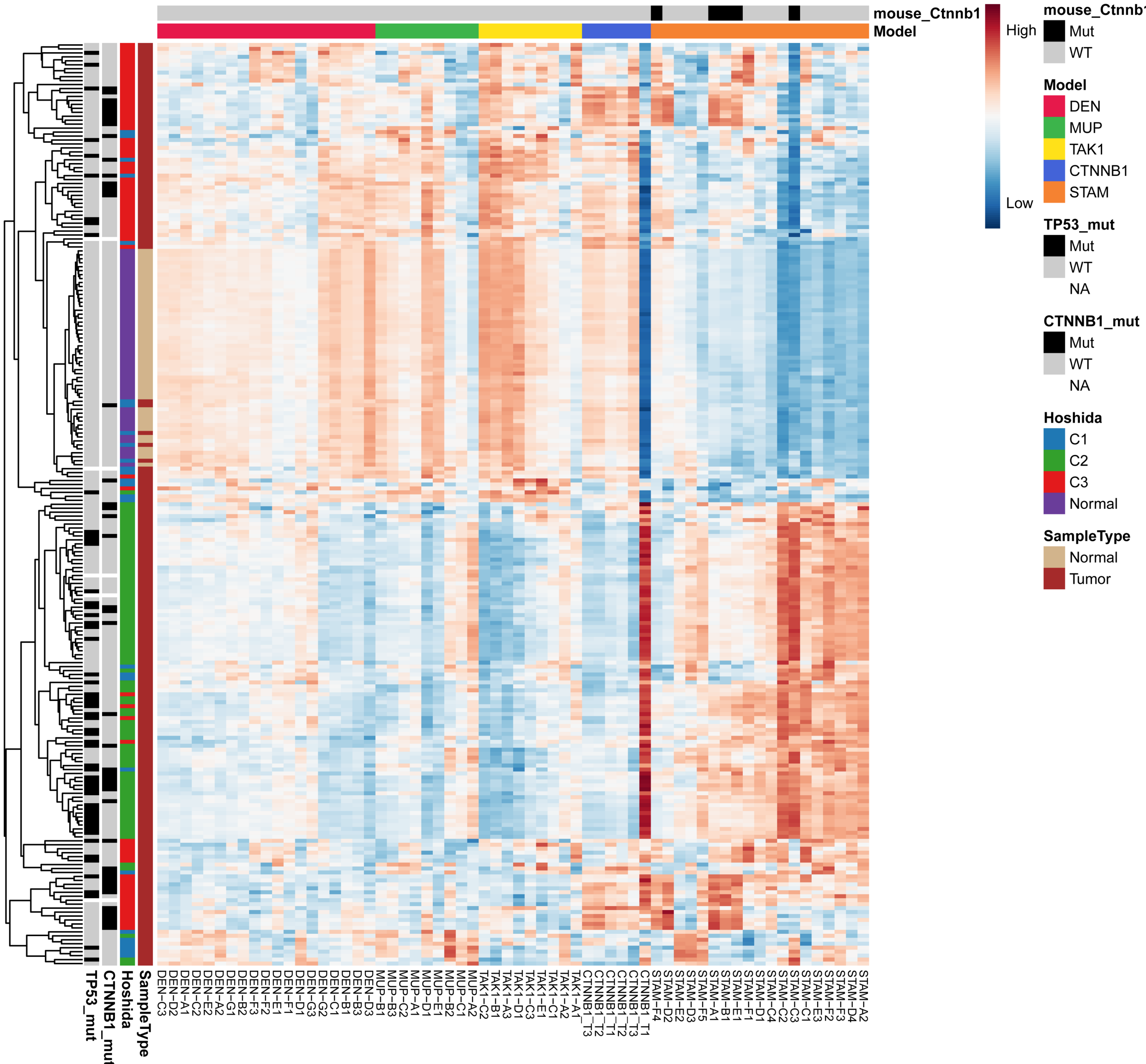

Supplementary Figure 5. Gene-expression profile correlations between mouse tumors and human HCCs.

Pairwise comparisons of mouse tumors grouped by model (columns) and human liver samples (normal and HCC) (rows). Ctnnb1 mutational status is listed for all mouse tumors on top. CTNNB1 and TP53 mutational status is listed for all human HCC tumors on the left. Human sample type and Hoshida clustering assignments of human HCCs are listed on the left. Human HCCs were clustered based on pairwise similarities with mouse tumors using 800 HCC-related gene-expression levels. Red shading indicates high gene-expression correlation between mouse and human tumors, whereas blue shading indicates low correlation. n=19, 9, 9, 6, 19 for DEN, MUP, TAK1, CTNNB1, and STAM, respectively. n=183 HCC patients.

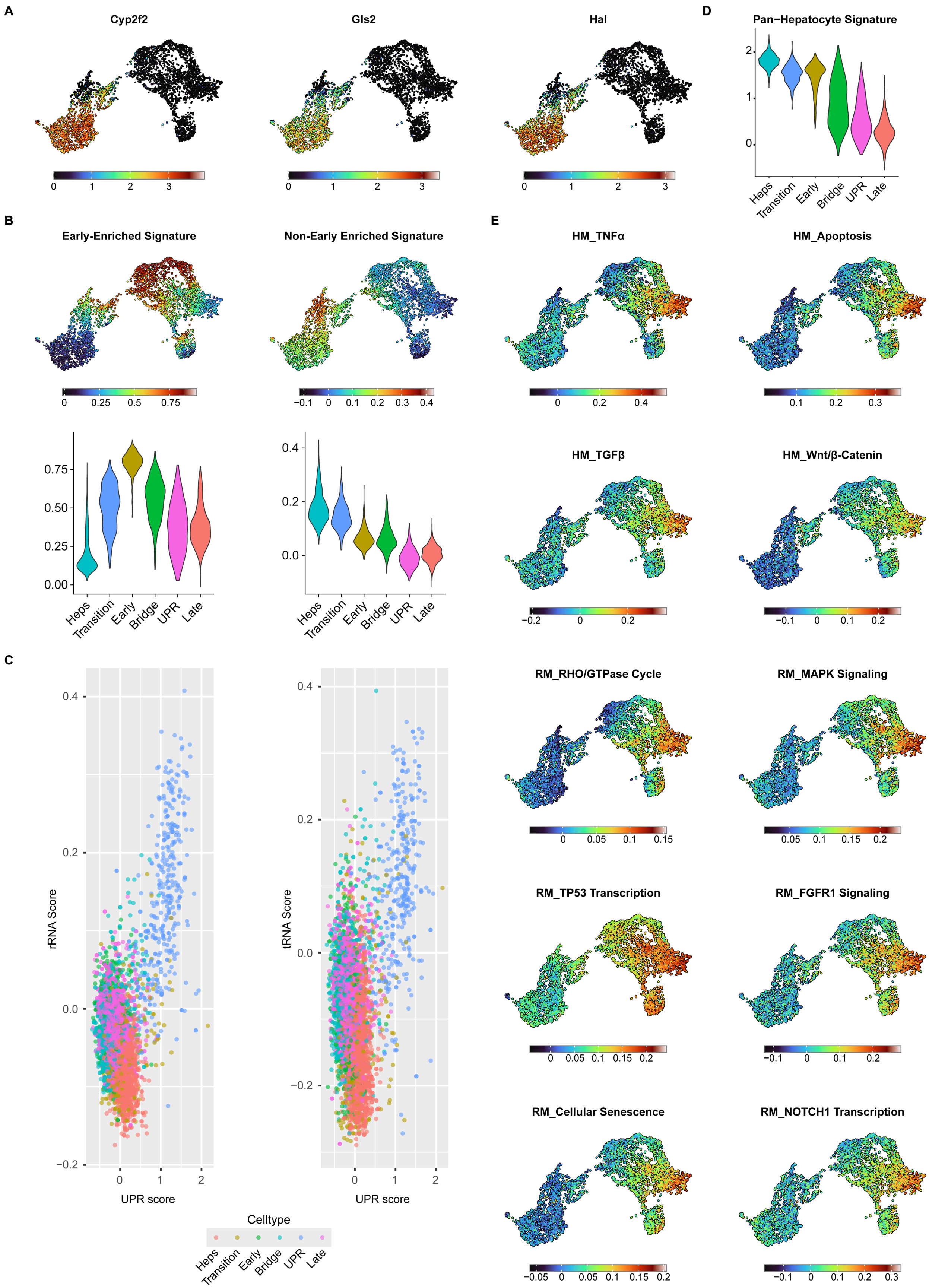
A
Cyp2f2
Gls2
Hal
D
Pan−Hepatocyte Signature
Heps
Transition
Early
Bridge
UPR
Late
B
Early-Enriched Signature
Non-Early Enriched Signature
E
HM_TNFα
HM_Apoptosis
HM_TGFβ
HM_Wnt/β-Catenin
C
rRNA Score
tRNA Score
UPR score
RM_RHO/GTPase Cycle
RM_MAPK Signaling
RM_TP53 Transcription
RM_FGFR1 Signaling
RM_Cellular Senescence
RM_NOTCH1 Transcription
Celltype

Supplementary Figure 6. Additional scRNA-seq characterization of β-catenin–mutant hepatocyte clusters in the normal liver.

A) UMAP plots showing expression of *Cyp2f2*, *Gls2*, and *Hal* in the dataset in Fig. 3. B) UMAP plots showing expression of the zone 3 signature (Fig. 3D) split in two sub-signatures based on whether the genes are enriched in the Early cluster. Relative enrichment levels for each cluster identified in Fig. 3C are depicted in a violin plot (below). C) Scatterplots showing rRNA and tRNA versus UPR gene set scores for all hepatocytes in the dataset in Fig. 3, with each cluster highlighted with different colors. D) Violin plots of relative pan-hepatocyte gene signature expression for each cluster identified in Fig. 3C. E) UMAP plots showing expression levels of the signatures built from the Hallmark and Reactome pathways identified in Fig. 3G.

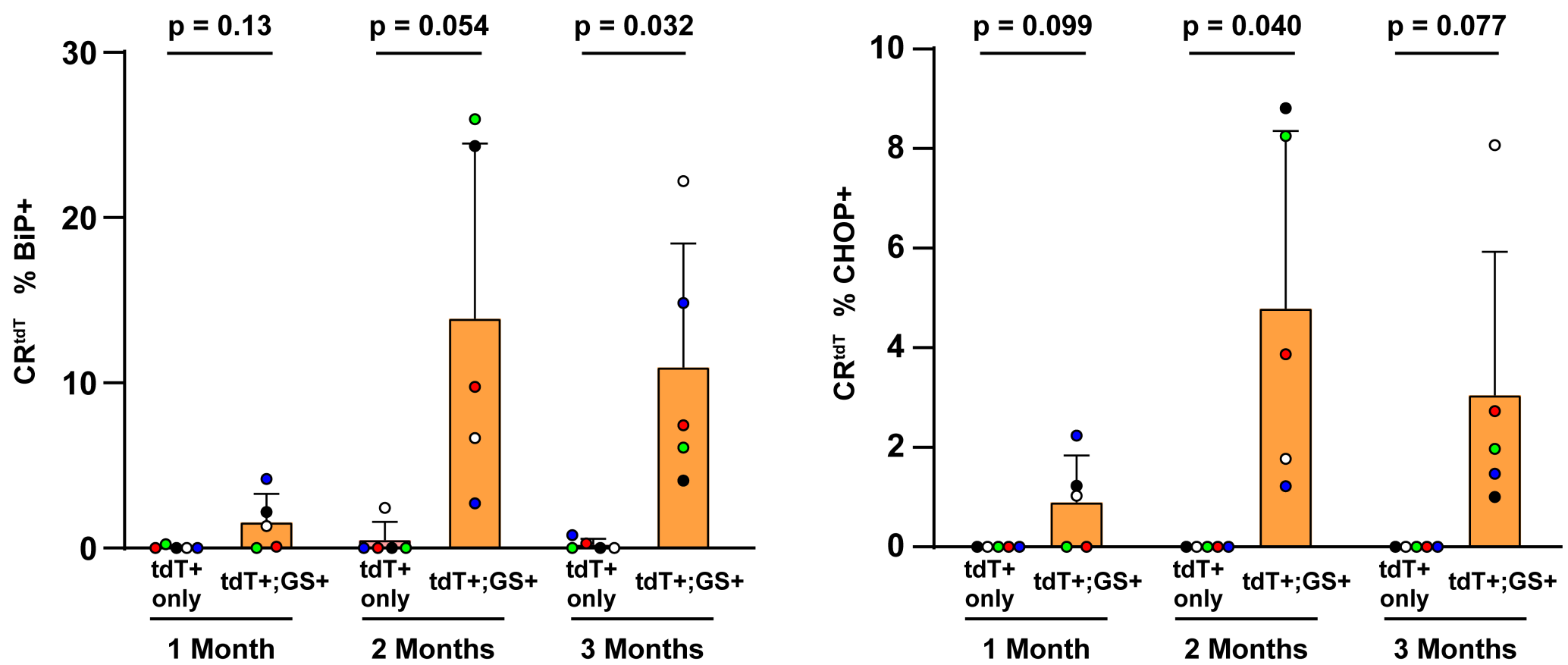

Supplementary Figure 7. CHOP and BiP are selectively elevated in *Ctnnb1^{ex3}* hepatocytes.

%tdT^{+} and tdT^{+};GS^{+} cells that are also CHOP^{+} or BiP^{+} from CR^{tdT} samples in Fig. 1C. Each point represents the mean count for each mouse, and each pair of colored points at each timepoint corresponds to the same mouse. Each bar represents the average of the mice means +SD. Statistical significance was determined between tdT^{+} and tdT^{+};GS^{+} groups at each timepoint using a paired, two-tailed t-test using the mean of the areas counted from each mouse. n=5. Source data are provided as a Source Data file.

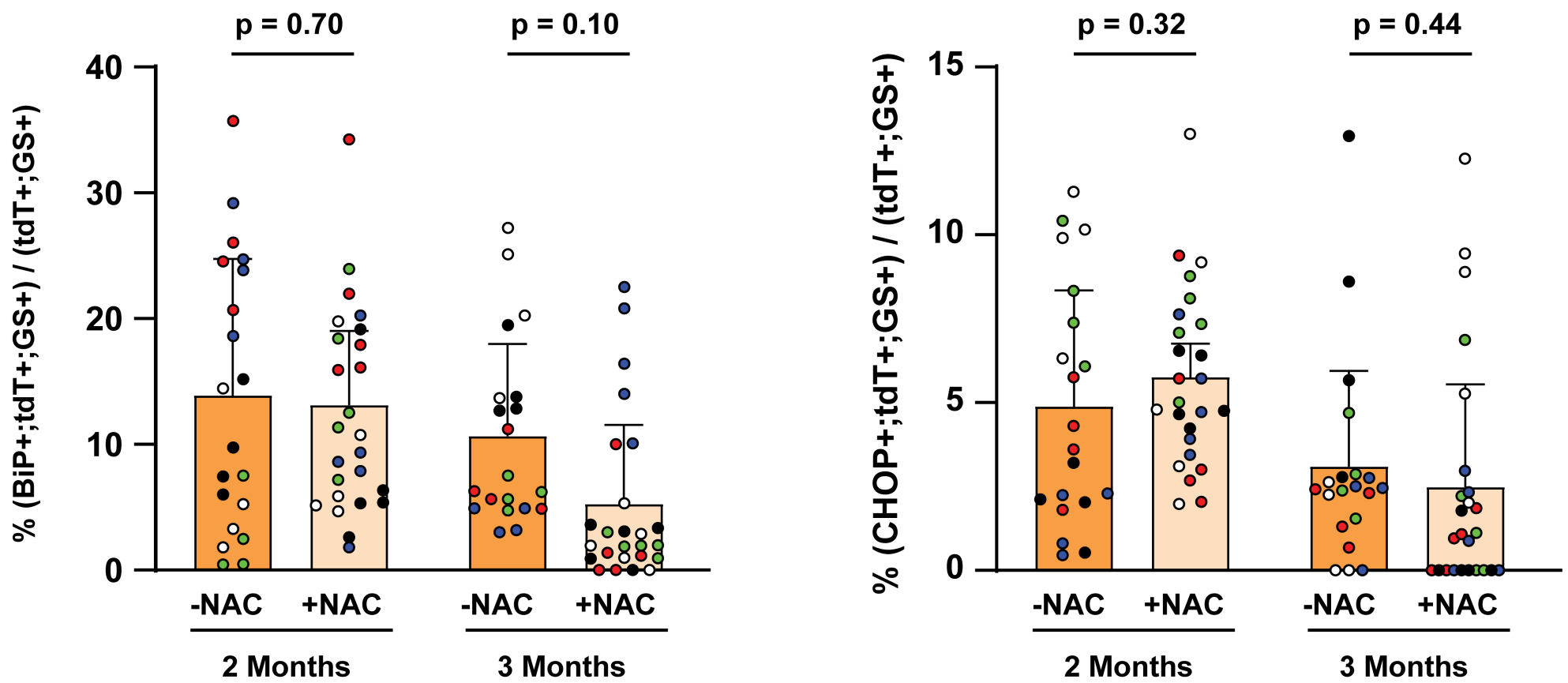

Supplementary Figure 8. NAC treatment does not significantly reduce UPR markers in *Ctnnb1*^{ex3} hepatocytes.

%tdT^{+};GS^{+} cells that are also CHOP^{+} or BiP^{+} from NAC treated CR^{tdT} samples in Fig. 4E, and CR^{tdT} samples from Fig. 1C-D. Each point represents an area counted within a lobe, and each set of colored points within a treatment group corresponds to the same mouse. Four or five areas were counted in each mouse. Each bar represents the average of the mice means +SD. Statistical significance was determined between -NAC and +NAC groups at each timepoint using an unpaired, two-tailed t-test using the mean of the areas counted from each mouse. n=5. Source data are provided as a Source Data file.

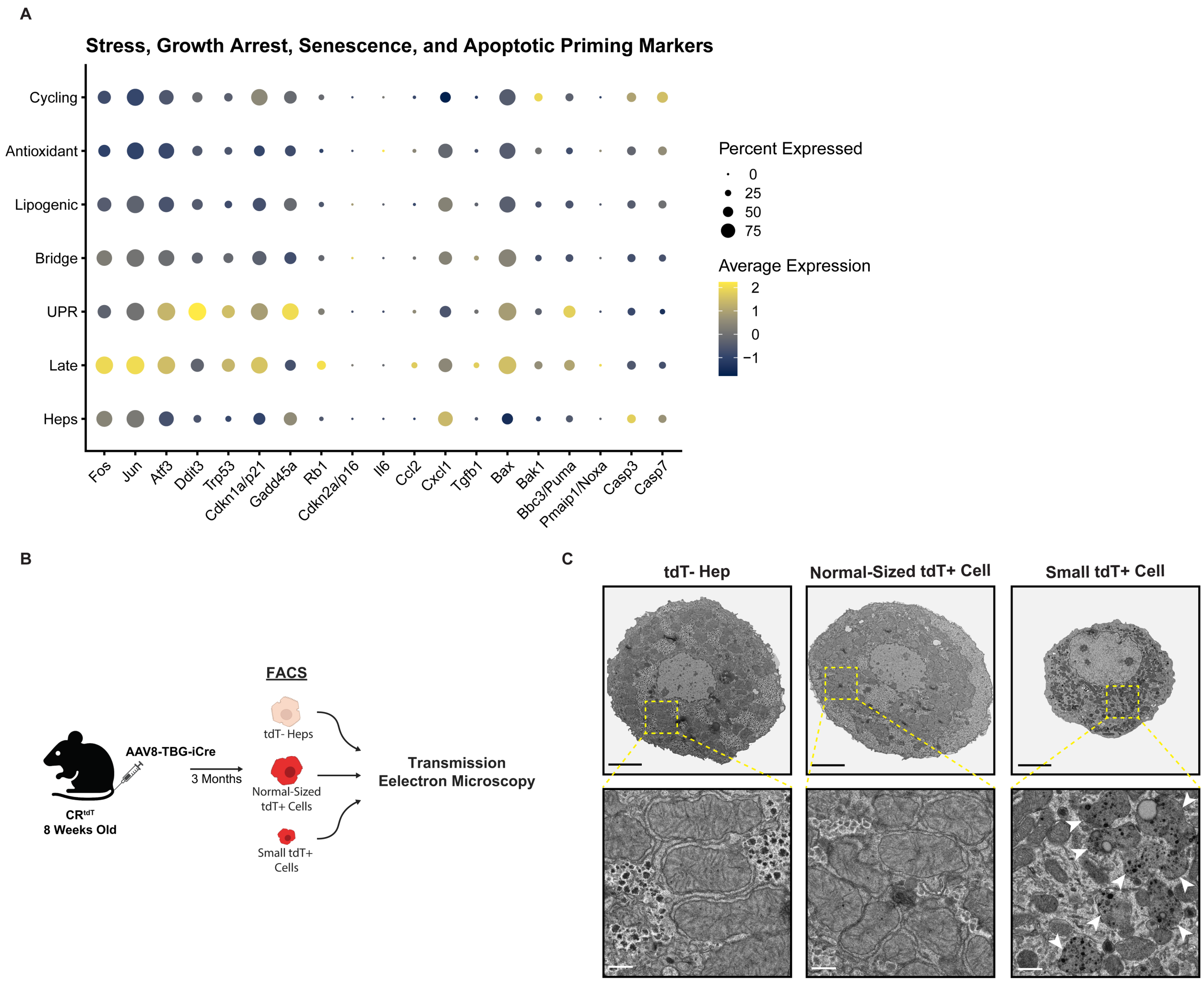

Supplementary Figure 9. Cellular stress pathways in *Ctnnb1^{ex3}* hepatocytes in the normal liver.

A) Dot plot of markers for cellular stress, growth arrest, senescence, and apoptotic priming in Ctnnb*1^{ex3}* hepatocytes from CMR^{tdT} mice in the dataset in Fig. 5. B) Schematic of experimental approach to image hepatocytes by electron microscopy. 3 months post-AAV injection in a CR^{tdT} mouse, hepatocytes were isolated and FACS sorted to separate tdT^{-} hepatocytes, Normal-Sized tdT^{+}, and Small tdT^{+} cells. Each cell population was fixed and imaged by transmission electron microscopy. C) Electron microscopy images of representative cells from the three populations isolated. Yellow-dashed boxes indicate the area displayed in higher magnification. Arrowheads highlight autolysosome structures. Findings were confirmed by imaging two independent mouse samples. Black scale bars=4μm, White scale bars=0.5μm. Panel B was created in BioRender. Hewett, C. (2026) https://BioRender.com/5pwn7dp.

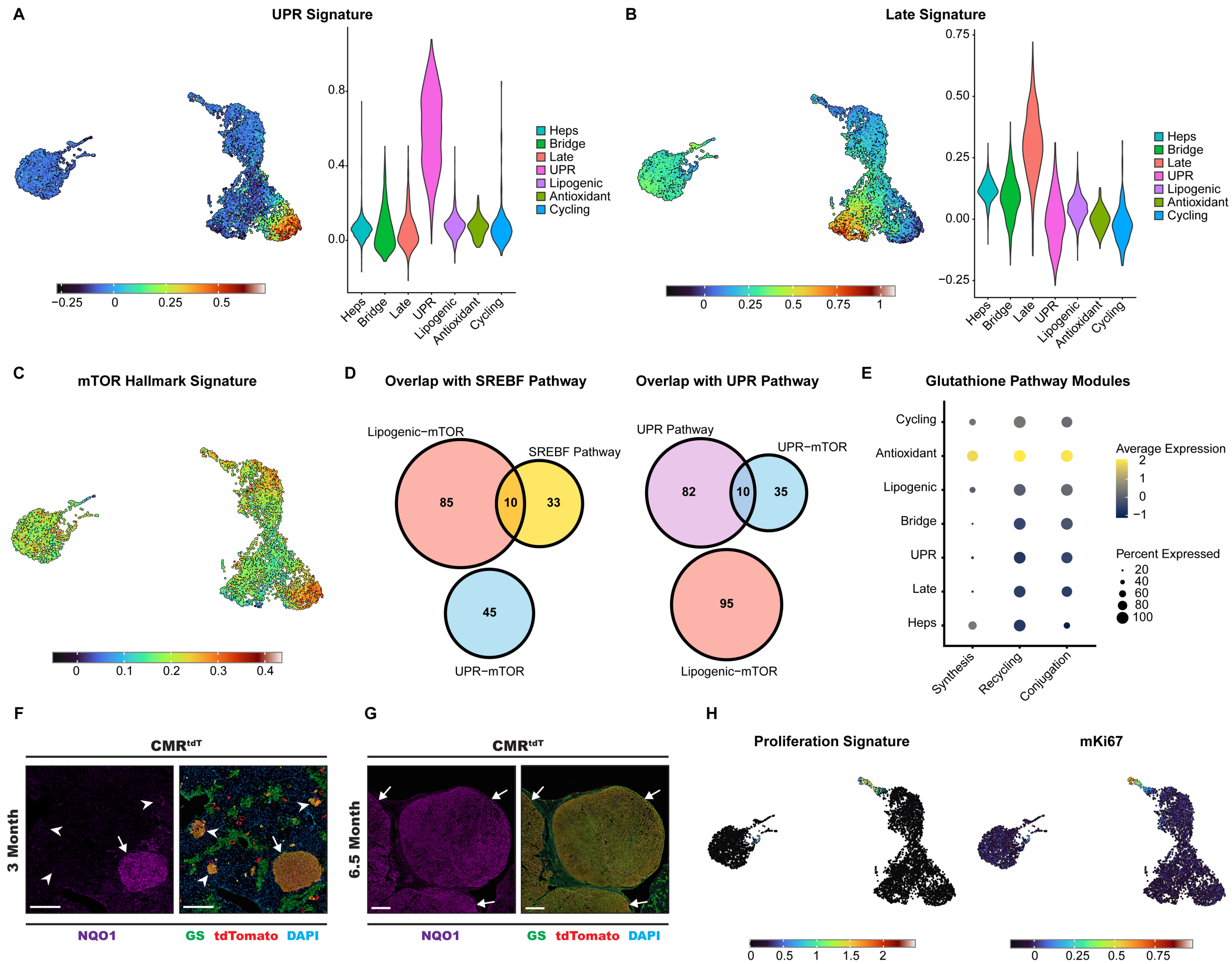

Supplementary Figure 10. Extended scRNA-seq and phenotypic characterization of β-catenin–mutant hepatocytes in chronic liver disease.

A-B) UMAP visualizations of UPR (A) and Late (B) gene signatures identified in Fig. 3 applied to the chronic liver damage dataset and corresponding violin plots. C) UMAP plot of the Hallmark mTOR signature corresponding to the Lipogenic and UPR clusters. D) Venn Diagram visualization of bivalent mTOR response overlap with SREBF or UPR pathways. Numbers within each colored area represent genes upregulated by the indicated pathway, with numbers in the overlapping areas indicating the number of upregulated genes shared by both pathways. E) Dot plot of glutathione metabolism in *Ctnnb1*^{ex3} hepatocytes from the dataset in Fig. 5. F-G) Representative immunofluorescence images of NQO1 staining in CMR^{tdT} mice at 3 (F) or 6.5 (G) months stained for tdT, GS, and NQO1. White arrows: tdT^{+};GS^{+} clones positive for NQO1. Arrowheads: small tdT^{+};GS^{+} clones negative for NQO1. Scale bars= 400μm (F) and 1mm (G). n=7 (F) and 6 (G). H) UMAP plots visualizing expression of a general proliferation signature and *mKi67*.

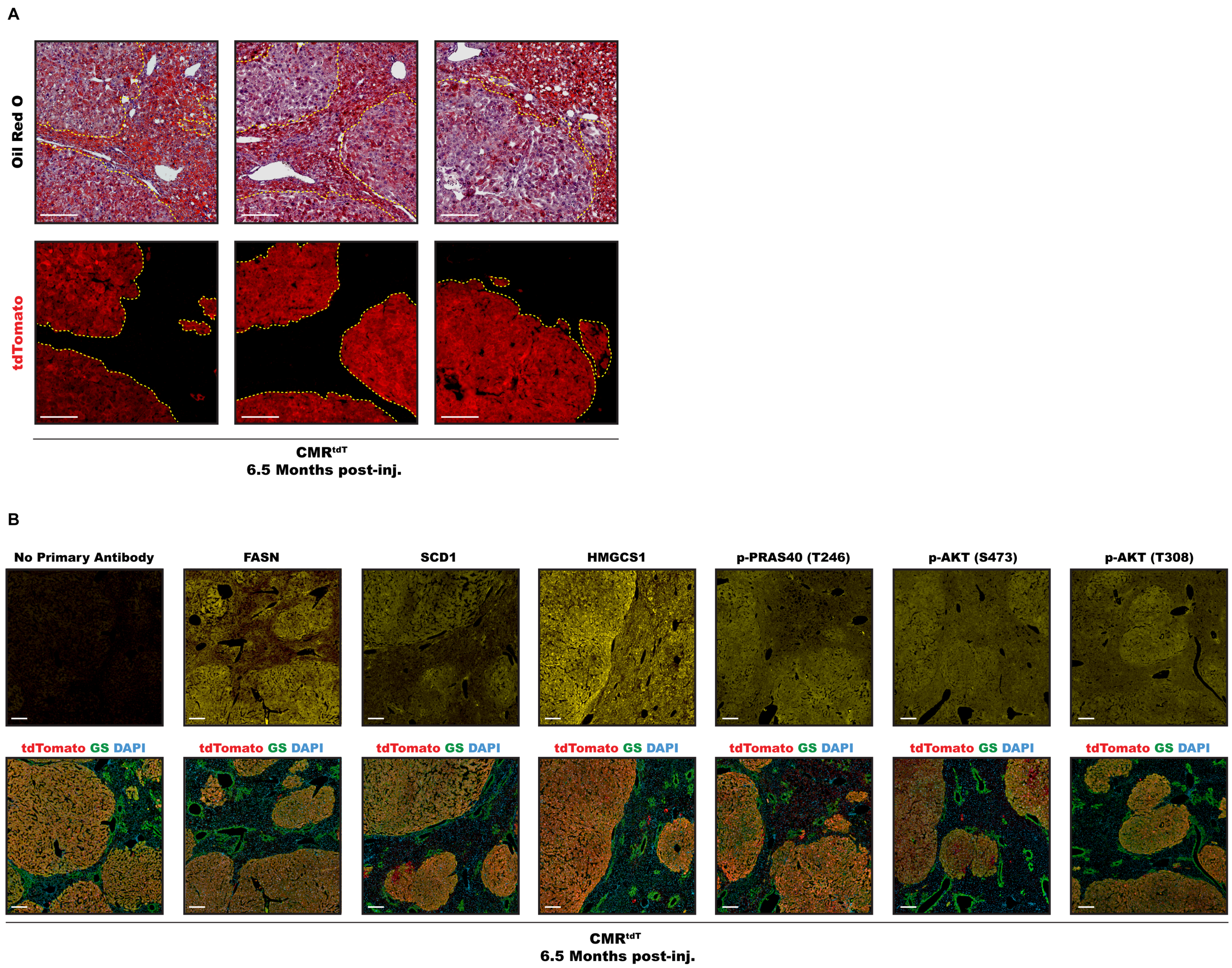

Supplementary Figure 11. Lipogenic markers are maintained in CMR^{tdT} tumors without neutral lipid accumulation.

A) Representative Oil Red O staining and endogenous tdTomato fluorescence in three independent CMR^{tdT} liver tissue areas 6.5 months post-AAV injection. Yellow-dashed lines circumscribe tdT^{+} tumors. Scale bars=200µm. n=6. B) Representative immunofluorescence images of CMR^{tdT} liver 6.5 months post-AAV injection stained with tdT, GS, and either No Primary Antibody, FASN, SCD1, HMGCS1, p-PRAS40 (T246), p-AKT (S473), or p-AKT (T308). Scale bars=200µm. n=6.

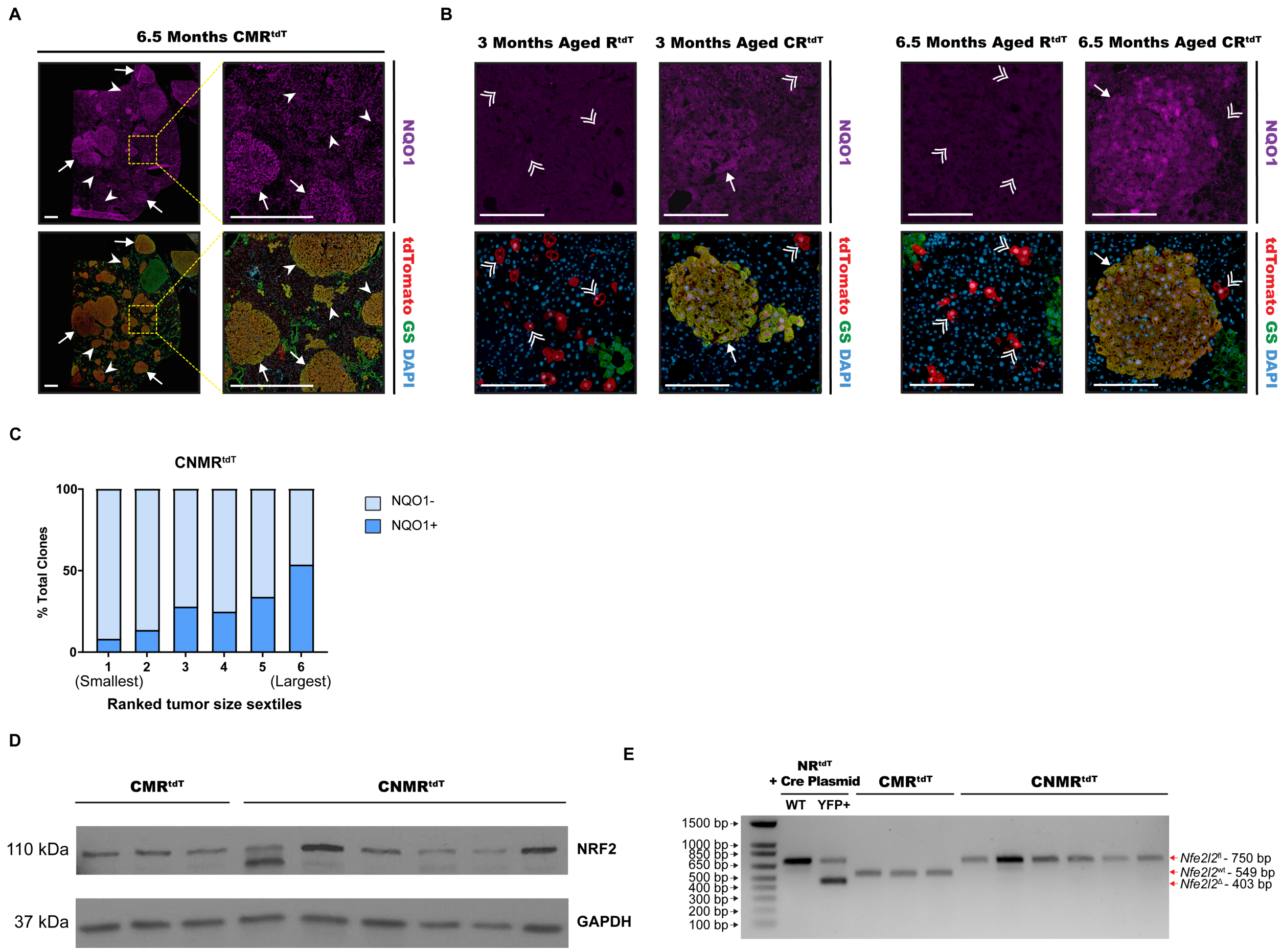

Supplementary Figure 12. NRF2-active (NQO1$^+$) β-catenin–mutant clones are enriched during clonal expansion and tumorigenesis.

A) Representative immunofluorescence images of CMR^{tdT} liver at the indicated timepoints post-AAV injection stained for tdT, GS, and NQO1. White arrows: tdT$^+$;GS$^+$ clones positive for NQO1. Arrowheads: tdT$^+$;GS$^+$ clones negative for NQO1. Yellow-dashed boxes: area displayed in higher magnification. Scale bars=1mm. B) Representative immunofluorescence pictures of Aged CR^{tdT} mice described in Fig. 2E at the indicated timepoints post-AAV injection stained for tdT, GS, and NQO1. White arrows: tdT$^+$;GS$^+$ clones. Double-arrowheads: tdT$^+$ only clones. Scale bars=200µm. C) %tdT+;GS+ clones in 6.5-month CNMR^{tdT} mice containing 5 cells or more that are either NQO1 positive or negative. Clones were divided into sextiles based on area. n=3. D) Western blot of excised liver tumors from CMR^{tdT} and CNMR^{tdT} mice 6.5 months post-AAV injection with the indicated antibodies. Each lane represents one tumor. E) Gel electrophoresis of *Nfe2l2* gene from hepatocytes isolated from NR^{tdT} mice injected with the Cre-containing plasmid described in Fig. 4C one-week post-HDTV and the same tumors as in (D). 549 bp band: wild-type *Nfe2l2* (*Nfe2l2^{wt}*); 750 base-pair (bp) band: non-recombined, floxed *Nfe2l2* (*Nfe2l2^{fl}*); 403 bp band: recombined *Nfe2l2* (*Nfe2l2$^{\Delta}$*). Source data are provided as a Source Data file.

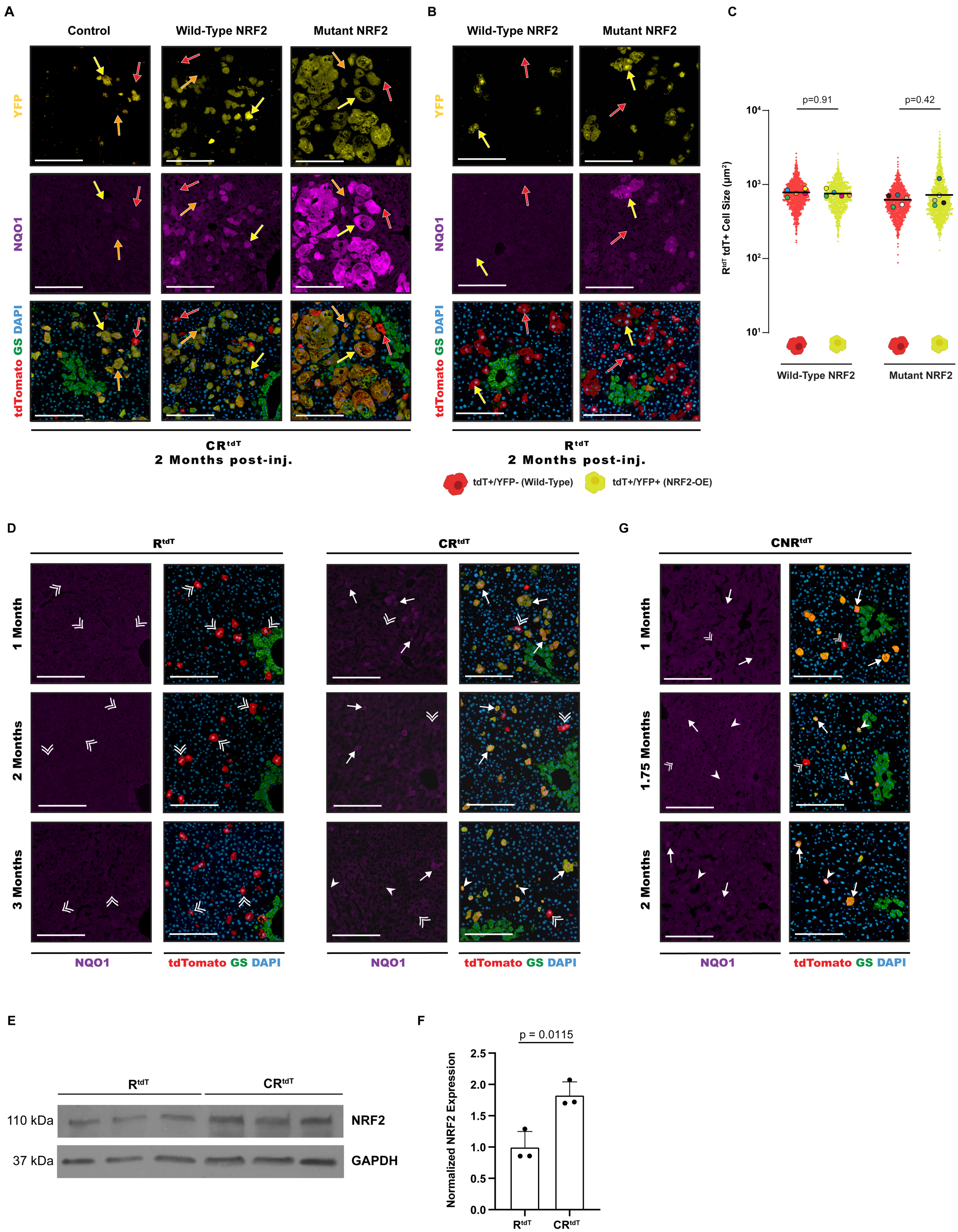
A
Control
Wild-Type NRF2
Mutant NRF2
YFP
NQO1
tdTomato GS DAPI
CRtdT
2 Months post-inj.
B
Wild-Type NRF2
Mutant NRF2
RtdT
2 Months post-inj.
tdT+/YFP- (Wild-Type)
tdT+/YFP+ (NRF2-OE)
C
p=0.91
p=0.42
RtdT tdT+ Cell Size (µm²)
Wild-Type NRF2
Mutant NRF2
D
RtdT
CRtdT
1 Month
2 Months
3 Months
NQO1
tdTomato GS DAPI
G
CNRtdT
1 Month
1.75 Months
2 Months
E
RtdT
CRtdT
110 kDa
NRF2
37 kDa
GAPDH
F
p = 0.0115
Normalized NRF2 Expression
2.5
2.0
1.5
1.0
0.5
0.0
RtdT
CRtdT

Supplementary Figure 13. NRF2 activity in *Ctnnb1^{ex3}* hepatocytes across experimental contexts in the normal liver.

A-B) Representative immunofluorescence pictures of (A) CR^{tdT} mice and (B) R^{tdT} mice injected with NRF2-containing transposons shown in Fig. 6E stained for tdT, GS, YFP, and NQO1. Red arrows: tdT^{+} only; orange arrows: tdT^{+};GS^{+};YFP^{-} hepatocytes (*Ctnnb1^{ex3}* hepatocytes); yellow arrows: tdT^{+};GS^{+};YFP^{+} hepatocytes (*Ctnnb1^{ex3}* hepatocytes + integrated transposon). Scale bars=200µm. C) Cell size quantification of the two hepatocyte populations in R^{tdT} mice injected with the transposons described in (B) 2 months post-HDTVi. Points are individual cells; colored dots are mouse means; black lines indicate the mean of mouse means. Same color indicates same mouse. Statistical significance between the two cell populations' cell sizes was determined by fitting a linear mixed effects model with a likelihood-ratio test. Significance of pairwise comparisons was determined using post-hoc comparisons from the fitted mixed-effects model, with two-sided tests. n=5 for each group. D) Representative immunofluorescence pictures of R^{tdT} and CR^{tdT} mice shown in Fig. 1B stained for tdT, GS, and NQO1. White arrows: normal size tdT^{+};GS^{+} cells. Arrowheads: small tdT^{+};GS^{+} cells. Double-arrowheads: tdT^{+} only cells. Scale bars=200µm. n=5 for all groups. E) Western blot of purified tdT^{+} hepatocytes from R^{tdT} and CR^{tdT} mice 1 month post-AAV injection with the indicated antibodies. Each lane represents one mouse. F) Normalized NRF2 expression from the western blot in (E). Each dot represents one mouse, with black lines indicating mean +SD. Statistical significance was determined between R^{tdT} and CR^{tdT} groups using an unpaired, two-tailed t-test. n=3 for each strain. G) Representative immunofluorescence pictures of CNR^{tdT} mice shown in Fig. 7C stained for tdT, GS, and NQO1. White arrows: normal size tdT^{+};GS^{+} cells. Arrowheads: small tdT^{+};GS^{+} cells. Double-arrowheads: tdT^{+} only cells. Scale bars=200µm. n=4, 4, and 3 for 1, 1.75, and 2 months, respectively. Panels B and C were created in BioRender. Hewett, C. (2026) https://BioRender.com/v3wb0p5. Source data are provided as a Source Data file.

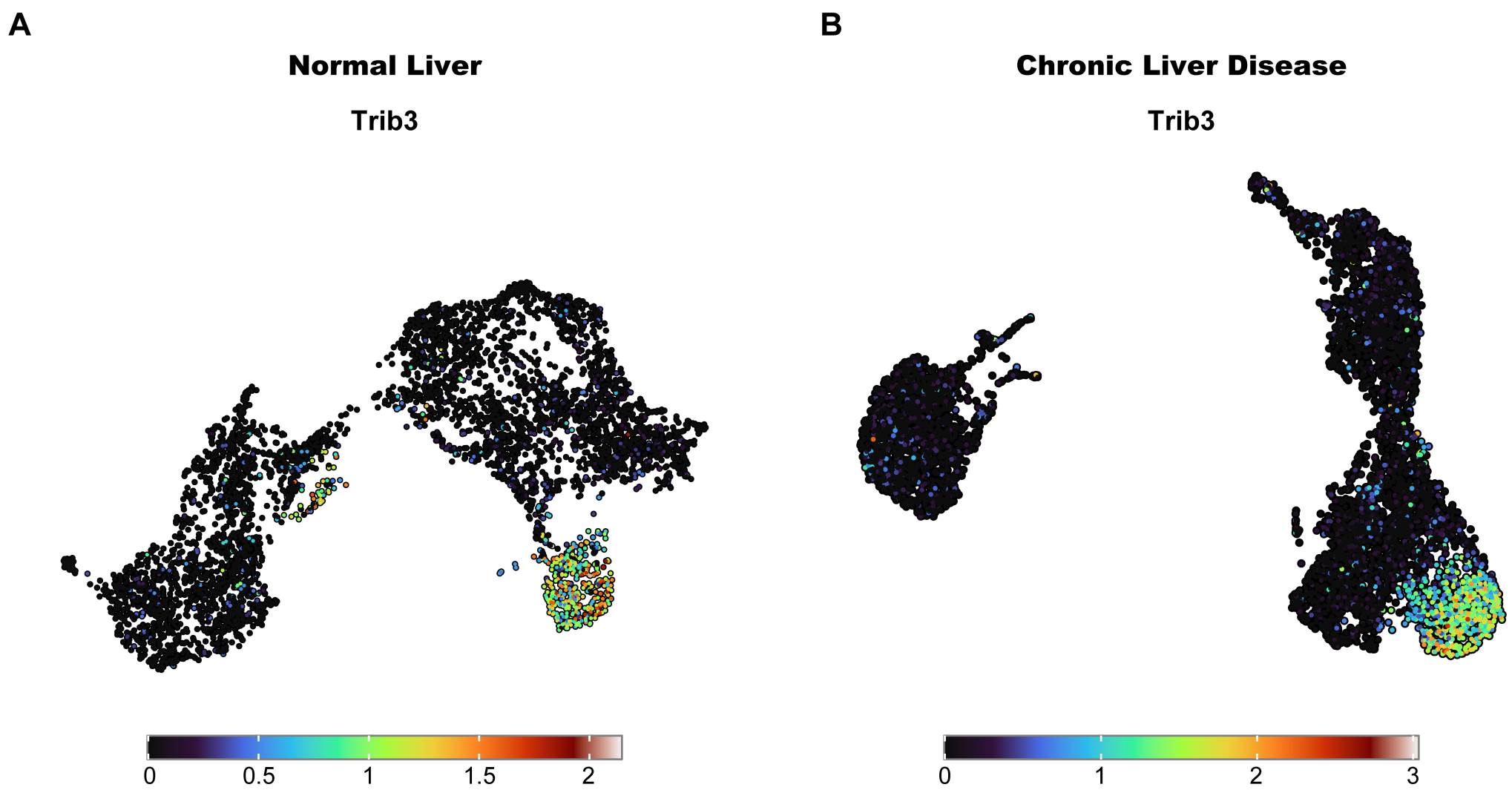

Supplementary Figure 14. Trib3 expression is enriched in the UPR cluster in both the normal liver and in chronic liver disease.

A-B) UMAP visualizations of *Trib3* (TRB3) in the (A) normal liver (Fig. 3) and (B) chronic liver damage (Fig. 5).

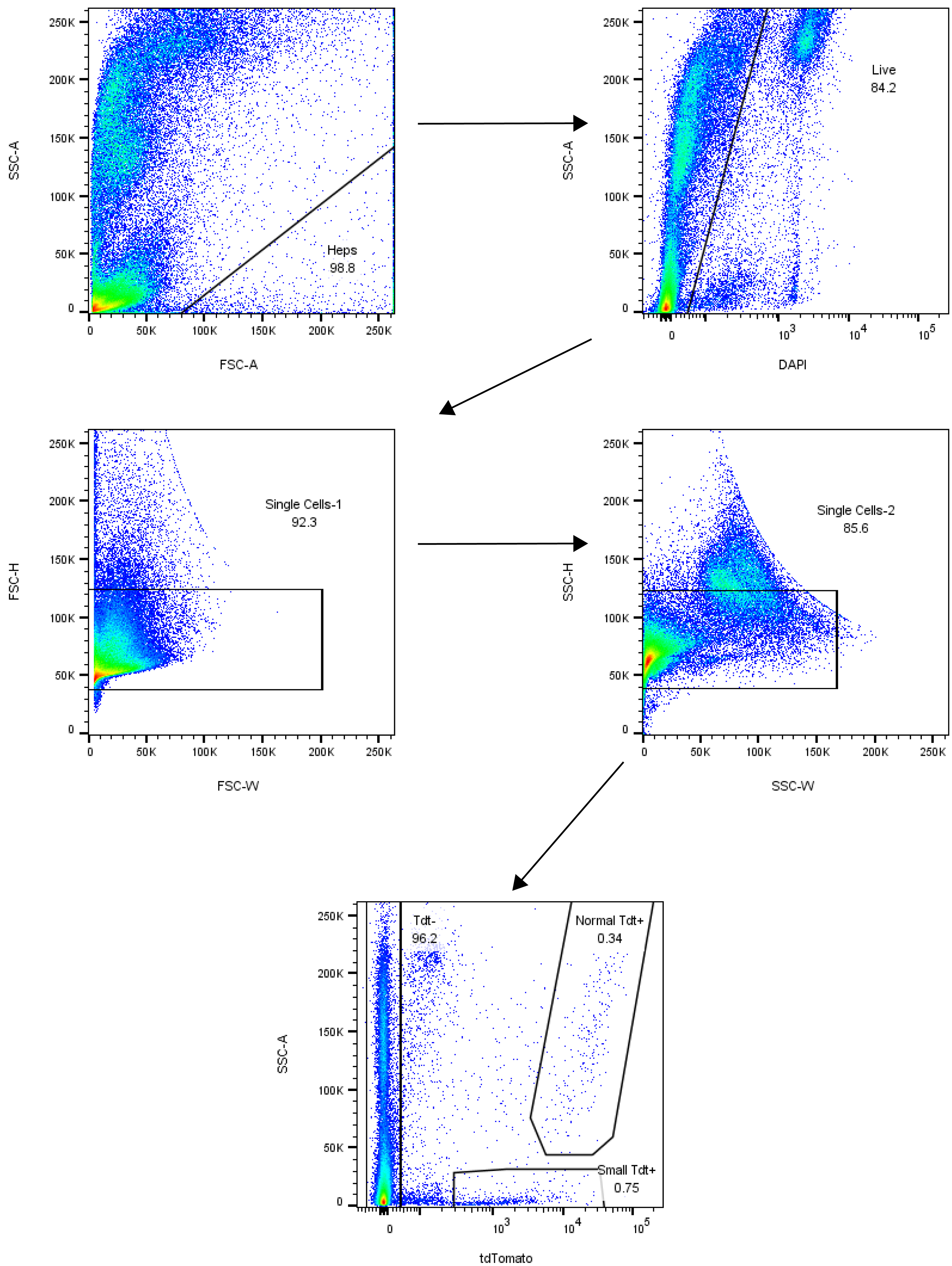

Heps
98.8
SSC-A
FSC-A
Live
84.2
DAPI
Single Cells-1
92.3
FSC-H
FSC-W
Single Cells-2
85.6
SSC-H
SSC-W
Tdt-
96.2
Normal Tdt+
0.34
Small Tdt+
0.75
tdTomato

Supplementary Figure 15. Flow cytometry gating strategy for sorting of tdT^- and tdT^+ mouse hepatocyte populations.

Example of the gating strategy used for sorting tdT^-, normal-sized tdT^+, and small tdT^+ hepatocyte populations isolated from the mouse liver.

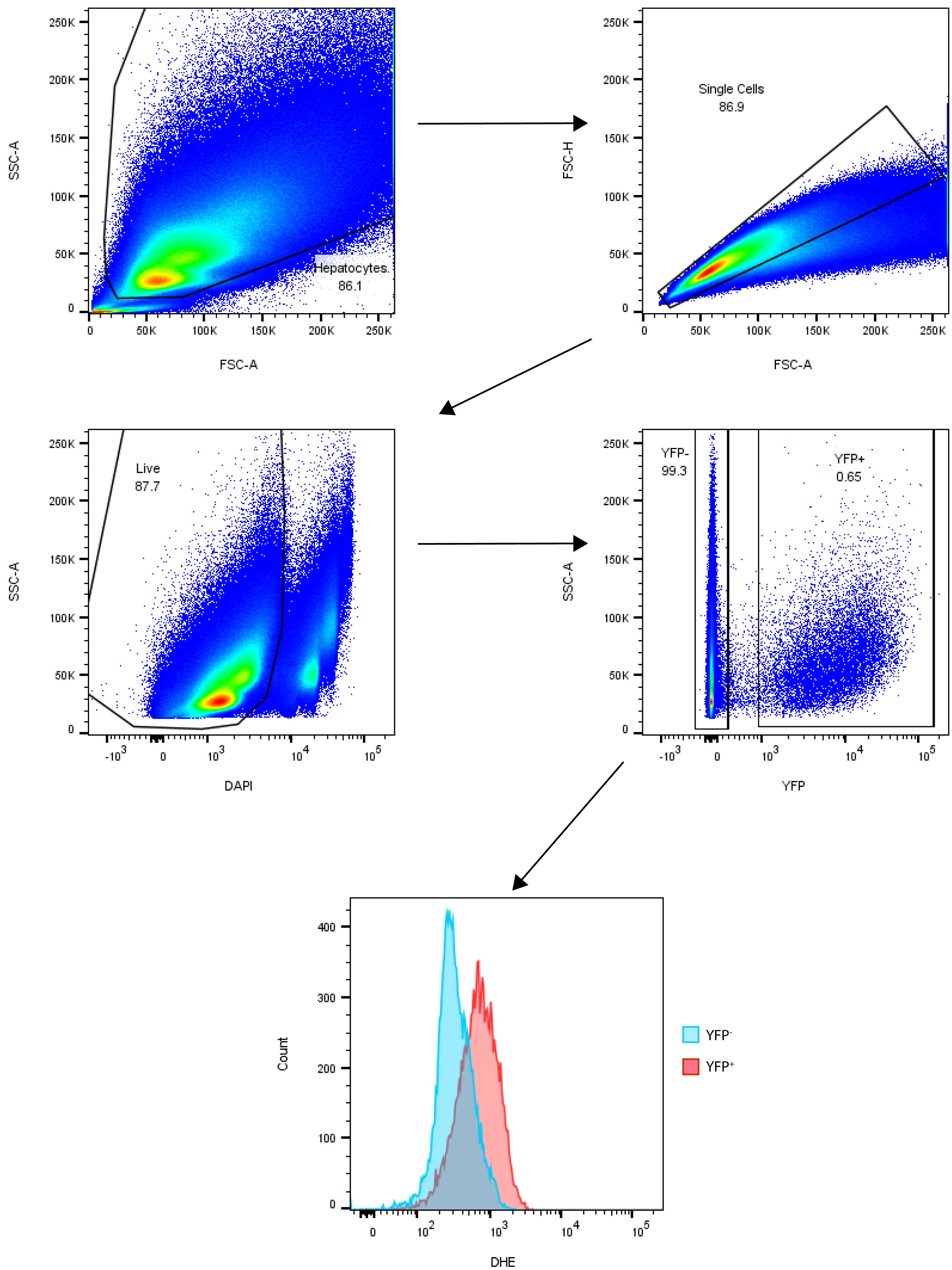
Hepatocytes
86.1
SSC-A
FSC-A
Single Cells
86.9
FSC-H
FSC-A
Live
87.7
SSC-A
DAPI
YFP-
99.3
YFP+
0.65
SSC-A
YFP
Count
DHE
YFP⁻
YFP⁺

Supplementary Figure 16. Flow cytometry gating strategy for DHE analysis of YFP^- and YFP^+ mouse hepatocyte populations.

Example of the gating strategy used for DHE analysis of hepatocytes from the mouse liver.
