# Supplementary material for: Adaptive oncogenesis of β-catenin emerges from declining liver tissue integrity
Source: Nat Commun. 2026 Aug 10;17:9565. doi: 10.1038/s41467-026-76373-y (PMC13550535; doi:10.1038/s41467-026-76373-y)
Supplement: Supplementary file 2 [file 41467_2026_76373_MOESM2_ESM.pdf]

nature portfolio

Corresponding author(s): Joan Font-Burgada

Last updated by author(s): Jul 22, 2026

Reporting Summary

Nature Portfolio wishes to improve the reproducibility of the work that we publish. This form provides structure for consistency and transparency in reporting. For further information on Nature Portfolio policies, see our Editorial Policies and the Editorial Policy Checklist.

Statistics

For all statistical analyses, confirm that the following items are present in the figure legend, table legend, main text, or Methods section.

n/a	Confirmed	
☐	☒	The exact sample size (*n*) for each experimental group/condition, given as a discrete number and unit of measurement
☐	☒	A statement on whether measurements were taken from distinct samples or whether the same sample was measured repeatedly
☐	☒	The statistical test(s) used AND whether they are one- or two-sided<br>*Only common tests should be described solely by name; describe more complex techniques in the Methods section.*
☐	☒	A description of all covariates tested
☐	☒	A description of any assumptions or corrections, such as tests of normality and adjustment for multiple comparisons
☐	☒	A full description of the statistical parameters including central tendency (e.g. means) or other basic estimates (e.g. regression coefficient) AND variation (e.g. standard deviation) or associated estimates of uncertainty (e.g. confidence intervals)
☐	☒	For null hypothesis testing, the test statistic (e.g. *F*, *t*, *r*) with confidence intervals, effect sizes, degrees of freedom and *P* value noted<br>*Give P values as exact values whenever suitable.*
☒	☐	For Bayesian analysis, information on the choice of priors and Markov chain Monte Carlo settings
☒	☐	For hierarchical and complex designs, identification of the appropriate level for tests and full reporting of outcomes
☒	☐	Estimates of effect sizes (e.g. Cohen's *d*, Pearson's *r*), indicating how they were calculated

Our web collection on statistics for biologists contains articles on many of the points above.

Software and code

Policy information about availability of computer code

Data collection	Nanozoomer S60 (Hamamatsu), Axio Observer 7 (Zeiss), FACSMelody (BD), FACSymphony S6 (BD), FACSAria II (BD), HiSeq X platform (Illumina, PE150), NovaSeq 6000 (Illumina, PE 150), NovaSeq X Plus (Illumina, PE 150)
Data analysis	GraphPad Prism 7.05, ImageJ 1.53q, FlowJo 10.8.1, Java 1.8.0_172 (64-bit), NDP.view2, CellRanger 8.0.0 (10x Genomics), R 4.2.1 (Seurat 5.0.2, SeuratObject 5.0.2, stringr 1.5.1, ggplot2 3.5.2, patchwork 1.3.1, decoupleR 2.3.3, enrichR 3.4, org.Mm.eg.db 3.15.0, dplyr 1.1.4, SCpubr 2.0.2, clusterProfiler 4.4.4, enrichplot 1.16.2, tibble 3.3.0, ReactomePA 1.40.0, KEGGREST 1.36.3, msigdbr 24.1.0, viridis 0.6.5, ComplexHeatmap 2.14.0, grid base package), fastp 0.23.1, Burrows-Wheeler Aligner 0.7.17, Sambamba 1.0.0, Picard 2.18.9, GATK 4.3.0, MuTect2 4.3.0, FREEC 11.4, Salmon 1.5.1

For manuscripts utilizing custom algorithms or software that are central to the research but not yet described in published literature, software must be made available to editors and reviewers. We strongly encourage code deposition in a community repository (e.g. GitHub). See the Nature Portfolio guidelines for submitting code & software for further information.

Data

Policy information about availability of data

All manuscripts must include a data availability statement. This statement should provide the following information, where applicable:

- Accession codes, unique identifiers, or web links for publicly available datasets
- A description of any restrictions on data availability
- For clinical datasets or third party data, please ensure that the statement adheres to our policy

All data are available in the main text or the supplementary information. Raw RNA sequencing data have been deposited in the GEO database under accession codes GSE306578 [https://www.ncbi.nlm.nih.gov/geo/query/acc.cgi?acc=GSE306578] and GSE334406 [https://www.ncbi.nlm.nih.gov/geo/query/acc.cgi?acc=GSE334406]. Raw exome sequencing data has been deposited in the SRA database under accession code PRJNA1465411 [https://www.ncbi.nlm.nih.gov/sra/PRJNA1465411]. The remaining data are available within the Article, Supplementary Information or Source Data file.

Research involving human participants, their data, or biological material

Policy information about studies with human participants or human data. See also policy information about sex, gender (identity/presentation), and sexual orientation and race, ethnicity and racism.

Reporting on sex and gender	*Use the terms sex (biological attribute) and gender (shaped by social and cultural circumstances) carefully in order to avoid confusing both terms. Indicate if findings apply to only one sex or gender; describe whether sex and gender were considered in study design; whether sex and/or gender was determined based on self-reporting or assigned and methods used.*<br>*Provide in the source data disaggregated sex and gender data, where this information has been collected, and if consent has been obtained for sharing of individual-level data; provide overall numbers in this Reporting Summary. Please state if this information has not been collected.*<br>*Report sex- and gender-based analyses where performed, justify reasons for lack of sex- and gender-based analysis.*
Reporting on race, ethnicity, or other socially relevant groupings	*Please specify the socially constructed or socially relevant categorization variable(s) used in your manuscript and explain why they were used. Please note that such variables should not be used as proxies for other socially constructed/relevant variables (for example, race or ethnicity should not be used as a proxy for socioeconomic status).*<br>*Provide clear definitions of the relevant terms used, how they were provided (by the participants/respondents, the researchers, or third parties), and the method(s) used to classify people into the different categories (e.g. self-report, census or administrative data, social media data, etc.)*<br>*Please provide details about how you controlled for confounding variables in your analyses.*
Population characteristics	*Describe the covariate-relevant population characteristics of the human research participants (e.g. age, genotypic information, past and current diagnosis and treatment categories). If you filled out the behavioural & social sciences study design questions and have nothing to add here, write "See above."*
Recruitment	*Describe how participants were recruited. Outline any potential self-selection bias or other biases that may be present and how these are likely to impact results.*
Ethics oversight	*Identify the organization(s) that approved the study protocol.*

Note that full information on the approval of the study protocol must also be provided in the manuscript.

Field-specific reporting

Please select the one below that is the best fit for your research. If you are not sure, read the appropriate sections before making your selection.

☒ Life sciences ☐ Behavioural & social sciences ☐ Ecological, evolutionary & environmental sciences

For a reference copy of the document with all sections, see nature.com/documents/nr-reporting-summary-flat.pdf

Life sciences study design

All studies must disclose on these points even when the disclosure is negative.

Sample size	No statistical methods were used to determine sample sizes.
Data exclusions	No data were excluded from this study.
Replication	All experiments were replicated at least 3 times, except for select scRNA-seq data described in their respective figure legends.
Randomization	For all experiments, mice were randomly allocated to different experimental groups.
Blinding	Investigators were not blinded while performing experiments or analyses.

Reporting for specific materials, systems and methods

We require information from authors about some types of materials, experimental systems and methods used in many studies. Here, indicate whether each material, system or method listed is relevant to your study. If you are not sure if a list item applies to your research, read the appropriate section before selecting a response.

Materials & experimental systems

n/a	Involved in the study
☐	☒ Antibodies
☒	☐ Eukaryotic cell lines
☒	☐ Palaeontology and archaeology
☐	☒ Animals and other organisms
☒	☐ Clinical data
☒	☐ Dual use research of concern
☒	☐ Plants

Methods

n/a	Involved in the study
☒	☐ ChIP-seq
☐	☒ Flow cytometry
☒	☐ MRI-based neuroimaging

Antibodies

Antibodies used

Goat anti-mCherry (tdTomato), Sicgen, AB0040-200, 1:1500 (RtdT Samples), 1:500 (Transposons)
Mouse anti-GS, BD, 610517, 1:200
Rabbit anti-GFP (YFP), Invitrogen, A-11122, 1:100
Chicken anti-GFP (YFP), Novus, NB100-1614, 1:100
Mouse anti-CHOP, CST, 2895, 1:100 with TSA kit
Rabbit anti-BiP, CST, 3177, 1:100
Rabbit anti-NQO1, Sigma, HPA007308, 1:100
Goat anti-E-Cadherin, R&D Systems, AF748, 1:100
Rabbit anti-p-Akt(Ser473), CST, 4060S, 1:100
Rabbit anti-p-Akt(Thr308), CST, 4056S, 1:100
Rabbit anti-phospho-PRAS40 (Thr246), CST, 2997S, 1:100
Rabbit anti-Fatty Acid Synthase, CST, 3180S, 1:100
Rabbit anti-SCD1, CST, 2794S, 1:100
Rabbit anti-HMGCS1, Proteintech, 17643-1-AP, 1:100
Donkey anti-mouse 488, Invitrogen, A21202, 1:600
Donkey anti-goat 555, Invitrogen, A21432, 1:600
Donkey anti-goat 647, Invitrogen, A21447, 1:600
Donkey anti-goat 755, Invitrogen, SA5-10091, 1:600
Donkey anti-rabbit 555, Invitrogen, A31572, 1:600
Donkey anti-rabbit 647, Invitrogen, A31573, 1:600
Donkey anti-chicken 555, Jackson ImmunoResearch, 703-165-155, 1:600
Goat Anti-Mouse IgG (Biotin conjugated), Vector Laboratories, BA-9200, 1:200 with TSA kit
Rabbit anti-NRF2, GeneTex, GTX103322, 1:1000
Rabbit anti-GAPDH, CST, 2118, 1:5000
Anti-rabbit IgG (HRP-linked), CST, 7074, 1:2500 (NRF2), 1:5000 (GAPDH)

Validation

Antibodies were validated by the manufacturer.

Animals and other research organisms

Policy information about studies involving animals; ARRIVE guidelines recommended for reporting animal research, and Sex and Gender in Research

Laboratory animals

Mice between 6-9 weeks were used for experiments.

Wild animals

No wild animals were used in this study.

Reporting on sex

Male mice were used in all experiments. Because the Ctnnb1 mutation is significantly biased towards male HCC patients clinically, we used male mice for all experiments to better recapitulate human HCC.

Field-collected samples

No samples from this study were field-collected.

Ethics oversight

Animal experiments were performed according to protocol 26-06 approved by Fox Chase Cancer Center Institutional Animal Care and Use Committee (IACUC).

Note that full information on the approval of the study protocol must also be provided in the manuscript.

Plants

Seed stocks

Report on the source of all seed stocks or other plant material used. If applicable, state the seed stock centre and catalogue number. If plant specimens were collected from the field, describe the collection location, date and sampling procedures.

Novel plant genotypes

Describe the methods by which all novel plant genotypes were produced. This includes those generated by transgenic approaches, gene editing, chemical/radiation-based mutagenesis and hybridization. For transgenic lines, describe the transformation method, the number of independent lines analyzed and the generation upon which experiments were performed. For gene-edited lines, describe the editor used, the endogenous sequence targeted for editing, the targeting guide RNA sequence (if applicable) and how the editor was applied.

Authentication

Describe any authentication procedures for each seed stock used or novel genotype generated. Describe any experiments used to assess the effect of a mutation and, where applicable, how potential secondary effects (e.g. second site T-DNA insertions, mosiacism, off-target gene editing) were examined.

Flow Cytometry

Plots

Confirm that:

- [x] The axis labels state the marker and fluorochrome used (e.g. CD4-FITC).
- [x] The axis scales are clearly visible. Include numbers along axes only for bottom left plot of group (a 'group' is an analysis of identical markers).
- [x] All plots are contour plots with outliers or pseudocolor plots.
- [x] A numerical value for number of cells or percentage (with statistics) is provided.

Methodology

Sample preparation

Mouse livers were harvested following intracardiac perfusion with Heparin (Sigma-Aldrich; H3149) in PBS, followed by Zinc-Formalin (StatLab; BFZ-1), then post-fixed overnight in Zinc-Formalin. After PBS washes, livers were incubated in 100 mM Tris (pH 9.5) (Teknova; T1095) with 10 mM DTT (Sigma; 43816) for 2 h at room temperature. Tissues were cryoprotected by overnight incubations in 15% and then 30% sucrose, and embedded in Tissue-Tek® OCT (Sakura, 4583) and stored at -80 °C.

To isolate hepatocytes and tdT^+ cells for single-cell RNA-sequencing and electron microscopy, mouse livers were perfused via the portal vein at 37 °C. Perfusion began with 50 mL of Ca^{2+}/Mg^{2+}-free HBSS supplemented with 10 mM HEPES and 0.5 mM EGTA (Alfa Aesar; J60767-AE) at 4 mL/min, followed by Ca^{2+}/Mg^{2+}-containing HBSS with 10 mM HEPES and 4 mg/mL Liberase TM (Sigma; 5401127001) at the same rate. Perfusion continued until clear signs of digestion occurred in the liver parenchyma under the liver capsule. Livers were excised, the gallbladder removed, and digested tissues transferred to 15 mL Ca^{2+}/Mg^{2+}-free HBSS. Liver capsules were gently disrupted to release the digested cells, which were then filtered through a 100 µm and 70 µm strainer and centrifuged at 50 × g for 1 min without brake. The pellet contained hepatocytes, while the supernatant contained non-parenchymal cells. Hepatocytes from the hepatocyte fraction were washed in Ca^{2+}/Mg^{2+}-free HBSS twice more, then resuspended in 30% Percoll-William's medium and centrifuged at 150 × g for 5 min. The resulting hepatocyte pellet was resuspended in William's medium, stained with 1 µg/mL DAPI, and sorted by FACS using a BD FACSAria II with a 100 µm nozzle. To isolate small tdT+ hepatocytes from the supernatant fraction, the supernatant from the first centrifugation step was separated and centrifuged at 1000 x g for 5 min to pellet the cells. The pellet was resuspended in Ca2+/Mg2+-free HBSS to wash the cells and centrifuged again at the same speed. The resulting pellet was resuspended at a concentration of 10 million cells per ml in 2% FBS-DMEM and sorted using a BD FACSAria II. For DHE experiments to analyze ROS activity, hepatocytes were isolated in the same way as described above. Hepatocytes were prepared in William's medium at 1x106 cells/mL and treated with DHE (Abcam; ab236206) for 30 minutes at 37 °C. Cells were centrifuged at 100 x g for 5 min to remove remaining DHE, resuspended in William's medium, and analyzed using a BD FACSymphony S6.

For Western blot analysis, liver tumors were excised following PBS-Heparin cardiac infusion and snap-frozen in liquid nitrogen.

For DNA isolation and genotyping, DNA was isolated from frozen liver tissues according to the DNeasy Blood & Tissue Kit (Qiagen; 69504) manufacturer's protocol.

For scRNAseq, cells were counted on a Zeiss Axio Observer and assessed for viability (>85% live) and singlet fraction. Sorted populations were pooled at a 1:1 ratio of tdT^- and tdT^+ cells, and loaded into a single well of a Next GEM Chip G. Cell partitioning into Gel Beads-in-emulsions (GEMs) and barcoding was carried out using the Chromium Controller, following the manufacturer's protocol. Barcoded cDNA libraries were prepared from GEMs for each mouse, and sequencing was performed on an Illumina HiSeq X platform (PE150).

For Electron Microscopy, cells were washed with PBS to remove the resuspension buffer and fixed with 2% paraformaldehyde (Electron Microscopy Sciences; 15710) and 2% glutaraldehyde (Electron Microscopy Sciences; 16020) in 0.1M sodium cacodylate buffer (Electron Microscopy Sciences; 11650) for one hour, and stored at 4 °C. Samples were washed with 0.1M sodium cacodylate buffer pH 7.4 with 2mM calcium chloride and then fixed with freshly prepared 1.5% potassium ferrocyanide and 1% osmium tetroxide in 0.1M cacodylate buffer pH 7.4 with 1mM calcium chloride for 2 h at RT. Samples were then en bloc stained with 1% uranyl acetate overnight at RT, protected from light. The following day, the samples were dehydrated with a graded series of acetone and then infiltrated with EMBed-812 resin. Samples were

polymerized in BEEM capsules or aluminum dishes with Embed-812 resin at 60˚C for 24-48 h. Using a Leica UC7 ultramicrotome, 60-70 nm thick sections were collected onto 200-mesh copper grids coated with a formvar/carbon film. The grids were post-stained with 1% uranyl acetate in 50% methanol and Reynolds lead citrate. Samples were imaged on a Zeiss Libra 120 TEM, and images were acquired with a Gatan Ultrascan 1000 CCD.

For RNA sequencing, RNA was extracted from excised 6.5 month CMRtdT liver tumors using the AllPrep DNA/RNA Mini Kit (Qiagen; 80204) according to the manufacturer's protocol. Samples mRNA libraries were prepared by Novogene with poly A enrichment and sequenced using a NovaSeq 6000 (PE 150).

For Whole Exome Sequencing, DNA was isolated from frozen liver tissues, two tumors and surrounding non-tumor bearing from five independent mice, according to the AllPrep DNA/RNA Mini Kit (Qiagen; 80204) manufacturer's protocol. The genomic DNA was randomly sheared into short fragments with the size of 180-280 bp. The obtained fragments were end repaired, A-tailed, and further ligated with Illumina adapters. Hybridization capture of libraries was proceeded according to the following procedures. Briefly, the prepped libraries were hybridized in the buffer with biotin-labeled probes, and magnetic beads with streptavidin were used to capture the exons of genes. Subsequently, non-hybridized fragments were washed out and probes were digested. The captured libraries were enriched by PCR amplification, and sequencing was performed using a NovaSeq X Plus (PE 150).

Instrument

Flow Cytometry for sequencing experiments and electron microscopy were done using the FACSAria II using a 100 µm nozzle. Samples for western blot using isolated cells were done with the FACSMelody using a 100 µm nozzle. All other experiments were done using the FACSymphony S6 with a 100 µm nozzle.

Software

BD FACSDiva and FACSChorus software was used to perform flow cytometric acquisition, analysis and sorting. Flow Jo software was used to process data for presentation.

Cell population abundance

Sorted cells were scanned on a Zeiss Axio Observer to obtain cell count and confirm >85% viability and presence of single cells. For scRNA-seq, sorted cell populations were pooled together in the following ratios: tdT- hepatocytes, 50%; tdT+ cells, 50%.

Gating strategy

Examples of gating strategies for cells analyzed and sorted are provided in Supplementary Information.

☒ Tick this box to confirm that a figure exemplifying the gating strategy is provided in the Supplementary Information.
