# Supplementary material for: Adaptive oncogenesis of β-catenin emerges from declining liver tissue integrity
Source: Nat Commun. 2026 Aug 10;17:9565. doi: 10.1038/s41467-026-76373-y (PMC13550535; doi:10.1038/s41467-026-76373-y)
Supplement: Supplementary file 3 [file 41467_2026_76373_MOESM3_ESM.pdf]

nature portfolio

Peer Review File

Adaptive Oncogenesis of β-Catenin Emerges from Declining Liver Tissue Integrity

Corresponding Author: Dr Joan Font-Burgada

This file contains all reviewer reports in order by version, followed by all author rebuttals in order by version.

Version 0:

Reviewer comments:

Reviewer #1

(Remarks to the Author)
In this study, the authors investigated the important question of how tissue state can regulate the expansion of mutant hepatocyte clones and tumorigenesis through the use of Ctnnb1-mutant murine models. Recent studies have shown that the known cancer-driving mutations are present in healthy tissues, suggesting that there are factors other than solely mutagenesis that contribute to cancer and disease progression. The authors utilize a liver-specific Ctnnb1ex3fl/+ ROSA26LSL-tdTomato/+ (CR) mouse model, as well as variations of this model, to demonstrate that in the normal liver, Ctnnb1 mutation leads to eventual elimination of mutant clones, even though it is a known oncogenic driver. They then add the MUP-uPA with HFD model of MASH to induce liver disease and find that in the MASH (CMR) model, Ctnnb1 mutation now induces tumorigenesis. They then performed scRNAseq on the CR mice and identified unique clusters of Ctnnb1 mutant hepatocytes, including transition (zone 1 and 3 signature), early (strong zone 3), and late (loss of hepatocyte identity, cellular stress). In addition, ER stress was implicated in the identity of the UPR cluster, and they found that Ctnnb1 mutant clones exhibited increase ROS and ER stress. The authors then repeated scRNAseq on the CMR livers and identified new clusters – lipogenic (fatty acid and cholesterol biosynthesis pathways, likely driven by mTOR and SREBF), antioxidant (cellular detoxification signature, likely driven by NRF2, high in tumors), and proliferating (closely associated with antioxidant), suggesting that a subset of Ctnnb1 mutant clones in the stressed liver upregulate certain programs that may lead to expansion and tumorigenesis. Since NRF2 seemed to be associated with expansion in the Ctnnb1 clones, the authors generated NRF2 knockout CMR mice (CNMR) and observed that most of the visible tumors in this model were NQO1+, suggesting that the NQO1- clones were eliminated. They then saw that Ctnnb1-mutant clones with constitutively active NRF1 exhibited enhanced survival and growth, suggesting that NRF2 signaling regulates the cell fate of Ctnnb1-mutant clones. Deleting endogenous NRF2 in the healthy liver accelerated Ctnnb1-mutant clearance, and AKT activation led to increased clonal expansion in CR mice as well as upregulation of NRF2 activity.

This is a well done study. The experiments are carefully performed and interpreted. The conceptual conclusions are sound and interesting.

Major Comments
1. The authors attempt to address an important topic in elucidating how known cancer-driving mutations work in tandem with environmental and other non-mutagenic factors to drive mutant clonal expansion and tumorigenesis. CTNNB1 mutations are highly prevalent in human cancer, and are also found in healthy livers, which presents an opportunity to explore this gap. Although the authors focus on the MASH model to induce chronic liver damage, I am interested to see if NRF2 and AKT signaling are also critical for tumorigenesis in other liver disease models, such as alcohol or carbon tet or HFD alone. Even a short term clonal expansion study with 1-2 of these additional injuries might be informative. Also, why does age mimic MUP-UPA? What injury is going on in the older population of mice?

2. In most of the experiments and mouse models other than the MASH model, the readout is mostly cell size and expansion based on immunofluorescence staining. We know now that clonal expansion of Ctnnb1 mutants, especially before the 4-6 month range, is not always directly correlated to the degree of later tumor development. Thus, it may be important to comment on whether or not increased NRF2 and AKT signaling in these mutants is sufficient to drive tumorigenesis in the non-MASH model (Fig. 7). Could there be other pathways critical for Ctnnb1-mutant tumorigenesis in these diseased livers? Also, in the tumor models, it would be good to show measurements such as liver weight/body weight ratio and surface tumor count, especially when comparing the CMR vs. CNMR tumors.

3. Recent studies have shown a difference between zone 1 and 3 mutant hepatocytes. Is the AAV leading to zone 3 mutants predominantly? Is there any difference in behavior of the zone 1 or 2 hepatocytes that have Ctnnb1 mutations?

4. Can the authors confirm if the tumors that form are actually only Ctnnb1 mutant? What about other TSG or oncogenes?

Minor comments
1. In Figure 4B, what is the frequency of Chop+ and Bip+ in the GS+/tdTomato double positive cells? This may focus on the Ctnnb1-mutant clones, as the GS-/tdTomato cells should experience relatively little ER stress compared to the GS+ cells.
2. In Figure 2, can you clarify the distinction between the single and multi clones and how they are evaluated differently?
3. In Figure 6, what are the relative frequencies of larger NQO1+ tumors to smaller/middling NQO1- tumors in the CNMR model?

Reviewer #2

(Remarks to the Author)
In this study, the authors investigate how tissue integrity modulates the oncogenic potential of activating Ctnnb1 mutations in the liver. Using long-term clonal lineage tracing and single-cell RNA sequencing, they compare Ctnnb1 mutated hepatocytes in healthy, aged, and chronically injured livers. They show that in young, healthy tissue, mutant hepatocytes remain quiescent and are progressively eliminated, whereas in aged or chronically injured livers they undergo clonal expansion and form tumors without additional oncogenic events. Mechanistically, elimination in intact tissue is linked to stress-induced unfolded protein response (UPR) pathways, while expansion in diseased tissue is associated with AKT–NRF2 activation. The authors propose that tissue integrity shapes the fitness landscape of β-catenin–mutant cells. Overall, the study provides important mechanistic insight into the context-dependent mutagenic potential of CTNNB1 and significantly advances our understanding of how tissue state shapes β-catenin–driven hepatocarcinogenesis.
Although comprehensive and supported by multiple genetic models, several aspects of the proposed model require clarification. The introduction states that β-catenin activation in healthy liver induces hepatomegaly (hyperplasia and hypertrophy) and death, yet the present data indicate that activation in a limited number of hepatocytes does not drive proliferation or clonal expansion. The discussion further notes that CTNNB1 mutations can lead to HCC in certain mouse models when a large fraction of hepatocytes is mutated. However, CTNNB1-mutated HCC also occurs in human non-cirrhotic livers. Clarifying how these observations align with the current findings would strengthen the manuscript.
The proposed mechanism for mutant hepatocyte attrition in healthy liver involves ROS-induced UPR activation. It would be important to clearly state whether an NRF2 response is detected in these cells, as suggested in Figure S4. Given that NFE2L2 is a β-catenin target gene (Gougelet et al., Hepatology 2014), its expression may be induced, but information regarding NRF2 stabilization and downstream target activation is needed. Moreover, while NAC treatment rescues mutant cell attrition, it remains unclear whether it resolves UPR activation and, more importantly, whether long-term ROS suppression would permit tumor development in an otherwise healthy liver.
The induction of a SREBP-dependent lipogenic program in mutant hepatocytes within chronically injured liver is intriguing, through a potential AKT-mediated SREBP1 activation. However, it is not demonstrated whether this transcriptional program results in lipid accumulation; neutral lipid staining would help address this point. In addition, human CTNNB1-mutated HCCs are rarely steatotic and typically display oxidative lipid metabolism, whereas AKT activation is more common in G1–G4 subclasses. G5-G6 classes display a lower proliferative rate than the other classes. It would therefore be informative to determine whether AKT signaling, lipogenesis, and NRF2 activation are maintained in tumors arising in the CMR model, or whether these early changes are transient.
To further resolve the part played by AKT activation in maintaining the mutated hepatocytes, the authors used a constitutively active AKT in their model, showing that it is sufficient to maintain CTNNB1 mutated hepatocytes in a sane liver allowing their rapid clonal expansion but a clonal expansion is also observed in the wild type CTNNB1 hepatocytes in those livers. How is it possible under these condition to separate the pro-proliferative aspect of AKT hyperactivation in their model to a specific function in the mutated hepatocytes?
Several minor points should also be addressed. (i) The nutritional status of mice at sacrifice (fed, refed, or fasted) is not specified, as metabolic pathways are highly nutrition-sensitive. (ii) Liver zonation should be clearly defined (zone 1 periportal; zone 3 pericentral/perivenous), particularly given its regulation by β-catenin. (iii) The statement that CTNNB1 mutations frequently co-occur with KEAP1 or NFE2L2 mutations should be reconsidered, as such associations are not statistically true (Savall et al., Hepatology Communications 2021). (iv) The authors suggest that lipogenesis may confer a selective advantage to transformed cells; further clarification of this point would be helpful.

Reviewer #3

(Remarks to the Author)
This study by Illendula et al. features many elegant reporter and lineage-tracing studies and introduces a potential new paradigm of tumor development dependent on mutational fitness. However, there were significant concerns with experimental design and data interpretation.

• The authors show that a subset of hepatocytes adopt an AKT-NRF2 permissive signaling state to unleash beta-catenin oncogenicity, but only allude to disease etiology, local tissue degeneration, and specific signaling pathways being engaged as possible reasons why only some hepatocytes develop this phenotype. Further studies are needed to describe the

mechanisms of this phenomenon. For example, is glutathione metabolism different in these cells?

• The beta-catenin mutated tumors do not replicate patient subtypes, since beta-catenin mutations occur concomitantly with other aberrations. Authors should demonstrate similarity to patient HCC subsets.

• The coactivation of mutated beta-catenin and NRF2 has been shown previously (PMID: 33529367); thus, this finding is not particularly novel.

• The authors demonstrate that AKT-NRF2 activation mediates beta-catenin-driven tumorigenesis. In addition to NRF2, beta-catenin interacts with Lkb1, Kras, and C-met, and these interactions can drive oncogenesis. Did the authors evaluate whether these were altered during hepatocyte expansion as tissue integrity deteriorated? The authors should also discuss whether these may also allow a permissive state for beta-catenin oncogenicity.

• Proliferation is a hallmark of beta-catenin mutated hepatocytes. Can the authors explain why proliferating Ctnnb1ex3 mutated hepatocytes were not present in the normal livers? Additionally, proliferation should be measured directly instead of through pathway analysis and size of tdTomato clusters.

• Constitutive activation of beta-catenin at early ages leads to oncogenesis as aging and chronic injury progress. However, would the authors expect similar changes in hepatocyte fitness and pathways if the beta-catenin mutation were introduced after the animals had aged and chronic injury had progressed?

• Would clonal expansion and RNA changes persist if animals were taken off HFD and continued to age (Figures 2 and 5)? Findings may provide evidence that treating chronic injury can prevent the expansion of mutant hepatocytes and attenuate tumorigenesis.

• Considering the authors link clonal expansion and tumorigenesis of beta-catenin to activation of the AKT-NRF2 axis, treating animals with a pharmacological agent that inhibits NRF2 to suppress mutant hepatocyte expansion and tumorigenesis would enhance the manuscript's translational relevance.

• Related to this, is NRF2 activation cell intrinsic (activated in hepatocytes with beta-catenin mutation because of UPR and oxidative stress) or extrinsic (activated in tissue microenvironment because of chronic injury)? This needs to be clarified, because it seems the authors are trying to make both arguments.

• In the discussion, the authors state that tissue-level changes modify the repertoire and frequency of cellular signaling states in which mutations interact, opening new phenotypic opportunities. The discussion should expand on this premise and discuss the potential role of the tumor microenvironment in mutant hepatocyte expansion.

• Some of the data seems to be overinterpreted or speculative. For example, in the clustering analysis in Figure 3, the two clusters (Heps and Ctnnb1ex3) are distinct, yet part of the Heps cluster expresses Glul, Axin2, and other beta-catenin target genes. The authors classify these as ‘transition’; however, because they are part of the Heps cluster, it is likely that they are normal zone 3 hepatocytes, which are constitutively active for beta-catenin and should be present in every single-cell RNA sequencing prep from normal liver. Additionally, there is no comparison between Ctnnb1ex3 hepatocytes and zone 3 hepatocytes in the same liver in terms of marker expression and pathways. Since zone 3 hepatocytes also have constant activation of beta-catenin, why do they not also undergo UPR and elimination?

• The conclusion that the stress-resilient state with upregulation of multiple signaling pathways makes Ctnnb1ex3 hepatocytes more susceptible needs additional justification and confirmation. The stress-resilient state also needs to be better defined; if these cells are activating pathways that resist stress, why are they being eliminated?

• Cell processes such as senescence and apoptosis should also be analyzed in the Late Ctnnb1ex3 hepatocytes to elucidate the mechanism by which they are eliminated.

• The mutational status of Nfe2l2 should be assessed in CMRtdT tumors to determine whether NRF2 activation occurs through mutation in the context of chronic injury or beta-catenin dependent changes in cellular fitness.

Minor:

• The figure legend for Figure 1C doesn’t match the figure panel. Only average points appear in the bar graphs. No individual points are shown.
• The inclusion of dot plots in Supplemental Figure 3 would help demonstrate the loss of hepatocyte identity and changes in signaling pathways in the Bridge, UPR, and Late clusters.
• The mouse models employed lack description. Mouse strain and sex need to be described in the methods and shown in figures. Would hormonal changes over time have a role in beta-catenin’s oncogenicity?

Reviewer #4

(Remarks to the Author)

I co-reviewed this manuscript with one of the reviewers who provided the listed reports. This is part of the Nature Communications initiative to facilitate training in peer review and to provide appropriate recognition for Early Career Researchers who co-review manuscripts.

Version 1:

Reviewer comments:

Reviewer #1

(Remarks to the Author)
Thank you for addressing our comments.

Reviewer #2

(Remarks to the Author)
The authors have satisfactorily addressed my concerns regarding the manuscript by providing additional data that strengthen the mechanistic insights and clarify several aspects of the study. One minor point remains regarding the immunostaining presented in Figure S11B. The inclusion of a negative control (e.g., incubation with the secondary antibody alone, without the primary antibody) would facilitate the interpretation of this panel, as the strong green autofluorescence known to be present in the liver appears to interfere with the specific signal. Overall, I believe that the authors have adequately addressed the issues raised during the review process.

Reviewer #3

(Remarks to the Author)
No additional changes or revisions are requested. All concerns have been addressed.

Open Access This Peer Review File is licensed under a Creative Commons Attribution 4.0 International License, which permits use, sharing, adaptation, distribution and reproduction in any medium or format, as long as you give appropriate credit to the original author(s) and the source, provide a link to the Creative Commons license, and indicate if changes were made.
In cases where reviewers are anonymous, credit should be given to 'Anonymous Referee' and the source.
The images or other third party material in this Peer Review File are included in the article's Creative Commons license, unless indicated otherwise in a credit line to the material. If material is not included in the article's Creative Commons license and your intended use is not permitted by statutory regulation or exceeds the permitted use, you will need to obtain permission directly from the copyright holder.
To view a copy of this license, visit https://creativecommons.org/licenses/by/4.0/

We thank the Referees for providing thoughtful, constructive feedback. All Referees' comments and suggestions have been addressed through additional experimentation and edits in the manuscript. As a result, the manuscript is much improved, for which we are sincerely grateful. Please find the point-by-point response to the Referees' comments below, which includes corresponding figure panels and text from the manuscript to facilitate the review process.

Referee #1:

In this study, the authors investigated the important question of how tissue state can regulate the expansion of mutant hepatocyte clones and tumorigenesis through the use of Ctnnb1-mutant murine models. Recent studies have shown that the known cancer-driving mutations are present in healthy tissues, suggesting that there are factors other than solely mutagenesis that contribute to cancer and disease progression. The authors utilize a liver-specific Ctnnb1ex3fl/+ ROSA26LSL-tdTomato/+ (CR) mouse model, as well as variations of this model, to demonstrate that in the normal liver, Ctnnb1 mutation leads to eventual elimination of mutant clones, even though it is a known oncogenic driver. They then add the MUP-uPA with HFD model of MASH to induce liver disease and find that in the MASH (CMR) model, Ctnnb1 mutation now indus tumorigenesis. They then performed scRNAseq on the CR mice and identified unique clusters of Ctnnb1 mutant hepatocytes, including transition (zone 1 and 3 signature), early (strong zone 3), and late (loss of hepatocyte identity, cellular stress). In addition, ER stress was implicated in the identity of the UPR cluster, and they found that Ctnnb1 mutant clones exhibited increase ROS and ER stress. The authors then repeated scRNAseq on the CMR livers and identified new clusters – lipogenic (fatty acid and cholesterol biosynthesis pathways, likely driven by mTOR and SREBF), antioxidant (cellular detoxification signature, likely driven by NRF2, high in tumors), and proliferating (closely associated with antioxidant), suggesting that a subset of Ctnnb1 mutant clones in the stressed liver upregulate certain programs that may lead to expansion and tumorigenesis. Since NRF2 seemed to be associated with expansion in the Ctnnb1 clones, the authors generated NRF2 knockout CMR mice (CNMR) and observed that most of the visible tumors in this model were NQO1+, suggesting that the NQO1- clones were eliminated. They then saw that Ctnnb1-mutant clones with constitutively active NRF1 exhibited enhanced survival and growth, suggesting that NRF2 signaling regulates the cell fate of Ctnnb1-mutant clones. Deleting endogenous NRF2 in the healthy liver accelerated Ctnnb1-mutant clearance, and AKT activation led to increased clonal expansion in CR mice as well as upregulation of NRF2 activity.

This is a well done study. The experiments are carefully performed and interpreted. The conceptual conclusions are sound and interesting.

We thank the Referee for the encouraging comments and positive feedback. We are grateful for the insightful suggestions, which have helped us to improve the manuscript.

Major Comments

1a. The authors attempt to address an important topic in elucidating how known cancer-driving mutations work in tandem with environmental and other non-mutagenic factors to drive mutant clonal expansion and tumorigenesis. CTNNB1 mutations are highly prevalent in human cancer, and are also found in healthy livers, which presents an opportunity to explore this gap. Although the authors focus on the MASH model to induce chronic liver damage, I am interested to see if NRF2 and AKT signaling are also critical for tumorigenesis in other liver disease models, such as alcohol or carbon tet or HFD alone. Even a short term clonal expansion study with 1-2 of these additional injuries might be informative.

Response to comment #1a: As suggested, we induced *Ctnnb1* mutations in CR^{tdT} mice treated with HFD-only or EtOH since these are clinically relevant models. Mice treated with HFD alone for 3 months (**Fig. S3**) had no clonal expansions compared to CMR^{tdT} mice, instead reducing in size similar to the normal liver model (**Fig. 1D**).

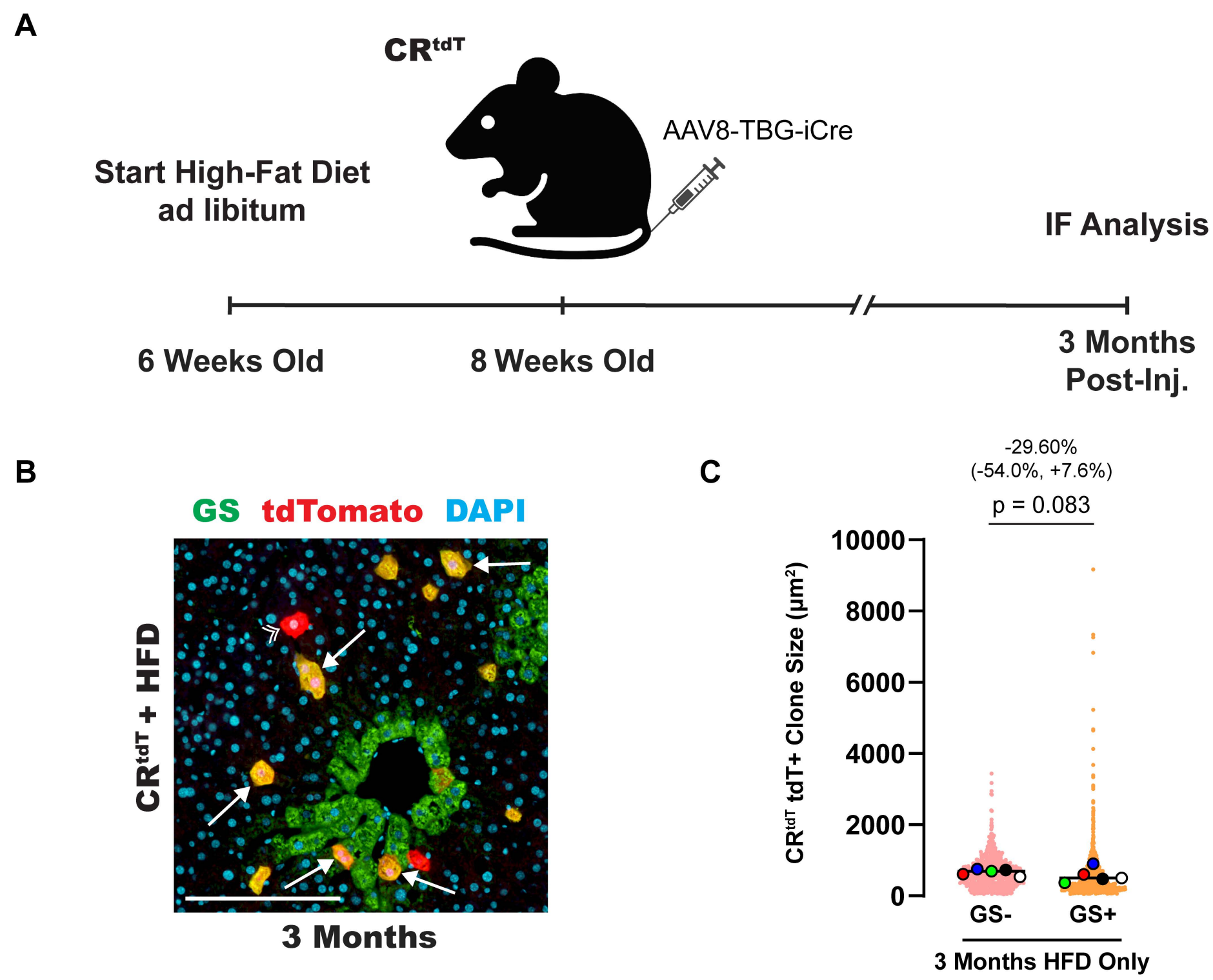

Fig. S3. *Ctnnb1^{ex3}* hepatocytes do not clonally expand with High-Fat Diet alone at 3 months. A) Schematic of High-Fat Diet treatment in CR^{tdT} mice experimental design. **B)** Representative immunofluorescence images of CR^{TdT} High-Fat Diet treated mice 3 months after injection stained for tdT and GS. White arrows: tdT^{+};GS^{+} clones, and arrowheads: tdT^{+};GS^{+} cells. Double arrowheads: tdT^{+} only cells. Scale = 200 µm. **C)** Quantification of tdT^{+};GS^{-} (GS^{-}) and tdT^{+};GS^{+} (GS^{+}) clone sizes in CR^{tdT} High-Fat Diet treated liver samples. Each point represents one clone, with each colored dot representing the mean clone size from each mouse. Same color indicates same mouse. Black solid lines represent the average of the mice means. Significance of pairwise comparisons was determined using post-hoc comparisons from the linear mixed model. The percentage change (top) and 95% confidence interval (bottom) of GS^{+} vs. GS^{-} cell size is listed above. n=5.

[Editorial note: panel a created in BioRender. Hewett, C. (2026) https://BioRender.com/t3qttns.]

In addition, we utilized the Lieber-DeCarli EtOH damage model. Unexpectedly, half of the mice died only 2 weeks after starting the diet, preventing a formal analysis robust enough to be included in the manuscript. Due to this, we prepared a referee only figure to present the trend found in the few remaining mice, which showed no clonal expansions at 1 month (**Referee Fig. 1**).

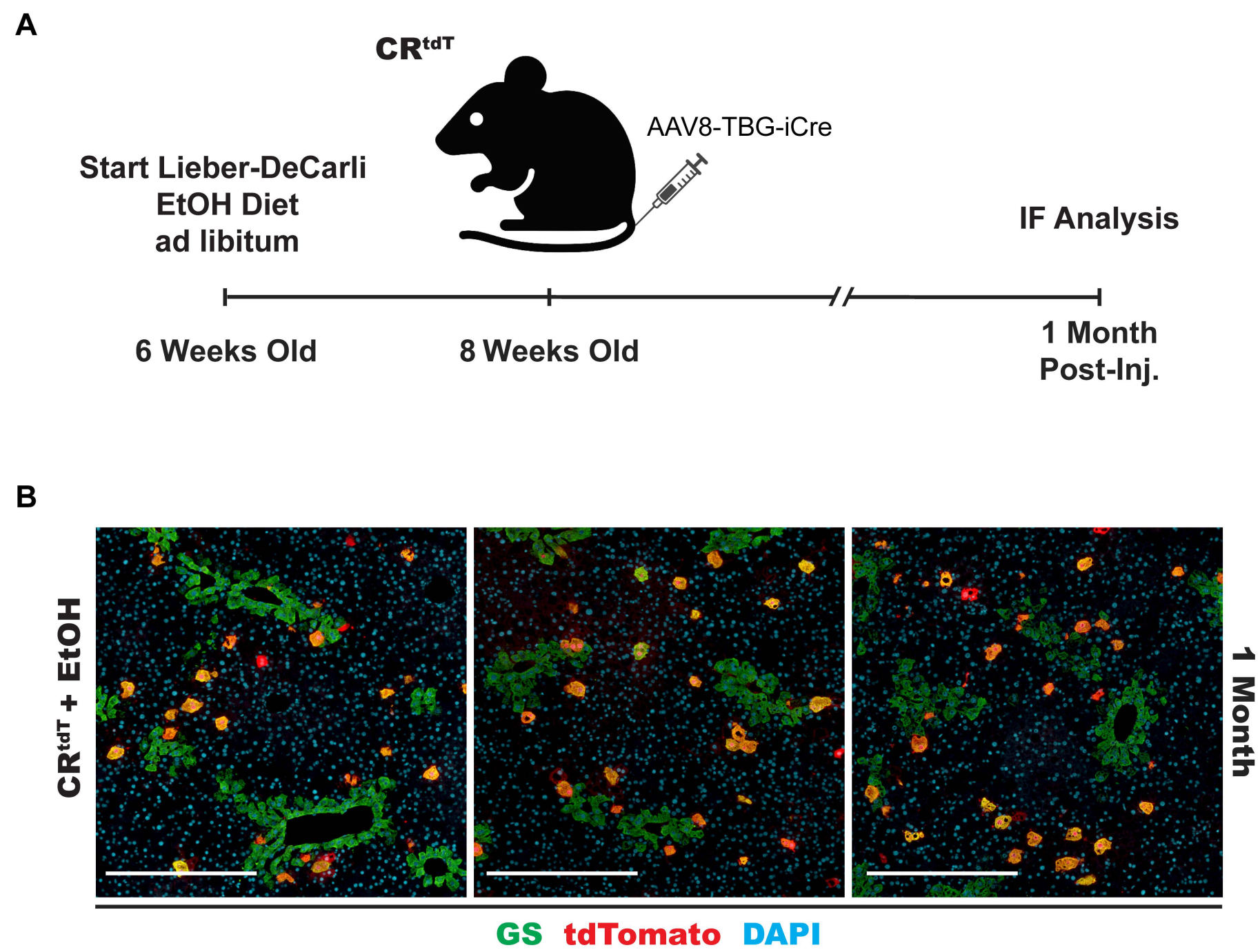

Referee Figure 1. Lieber-DeCarli EtOH diet in CR^{tdT} mice. A) Schematic of EtOH treatment in CR^{tdT} mice experimental design. **B)** Representative immunofluorescence images of CR^{tdT} EtOH treated mice 1 months after injection stained for tdT and GS. Scale = 400 µm.

Since these experiments don't allow us to draw any definitive conclusions regarding long term clonal expansions in additional CLD models, we decided to include the HFD-only data in the manuscript alongside the CMR^{tdT} model, to serve as a reference without the damage from the *MUP-uPA* transgene.

1b. Also, why does age mimic MUP-UPA? What injury is going on in the older population of mice?

Response to comment #1b: We chose CLD and aging models because the clinical risk for HCC increases with liver disease severity[1] and age[2]. Although we couldn't perform functional or genetic studies with aged mice due to logistical constraints, we found signs of NRF2 activation in expanding clones from aged livers (**Fig. S12B**), indicating that similar pathways are being activated in an aged liver context as with chronic damage. Even though overt damage is not seen in the aged liver, these results suggest that CLD and aging share common shifts in the fitness landscape, likely involving the AKT-NRF2 axis, that provide differential selective pressures on *Ctnnb1*-mutated hepatocytes.

2a. In most of the experiments and mouse models other than the MASH model, the readout is mostly cell size and expansion based on immunofluorescence staining. We know now that clonal expansion of Ctnnb1 mutants, especially before the 4-6 month range, is not always directly correlated to the degree of later tumor development. Thus, it may be important to comment on whether or not increased NRF2 and AKT signaling in these mutants is sufficient to drive tumorigenesis in the non-MASH model (Fig. 7). Could there be other pathways critical for Ctnnb1-mutant tumorigenesis in these diseased livers?

Response to comment #2a: While combining other oncogenes ectopically with *Ctnnb1* (Myc, c-Met, Yap, among others) has been shown to induce tumors in the normal liver, our scRNAseq data and experiments in the MASH model show that the AKT-NRF2 axis is the prominent driver for tumorigenesis without additional oncogenic mutations (please see response to comment #4). Importantly, ectopic activation of this axis together with mutant *Ctnnb1* is sufficient to overcome the maladaptive barriers present in the normal liver and promote clonal expansion. Nevertheless, we cannot exclude that additional injury-induced signaling pathways may cooperate with *Ctnnb1* mutations and further modulate tumor development in chronic liver disease.

We have revised the discussion to include: "**While additional pathways induced from chronic injury may also cooperate with oncogenic β-catenin, these findings identify the AKT-NRF2 axis as a dominant molecular gate that determines whether β-catenin mutated hepatocytes are positively or negatively selected.**"

2b. Also, in the tumor models, it would be good to show measurements such as liver weight/body weight ratio and surface tumor count, especially when comparing the CMR vs. CNMR tumors.

Response to comment #2b: We appreciate the Referee's suggestion, which helped us improve the presentation and interpretation of these data. We have revised the figure to include the complete set of $CNMR^{tdT}$ mice with corresponding macroscopic liver pictures, better illustrating the overall reduction in tumorigenesis compared to CMR^{tdT} mice (**Fig. 6C**).

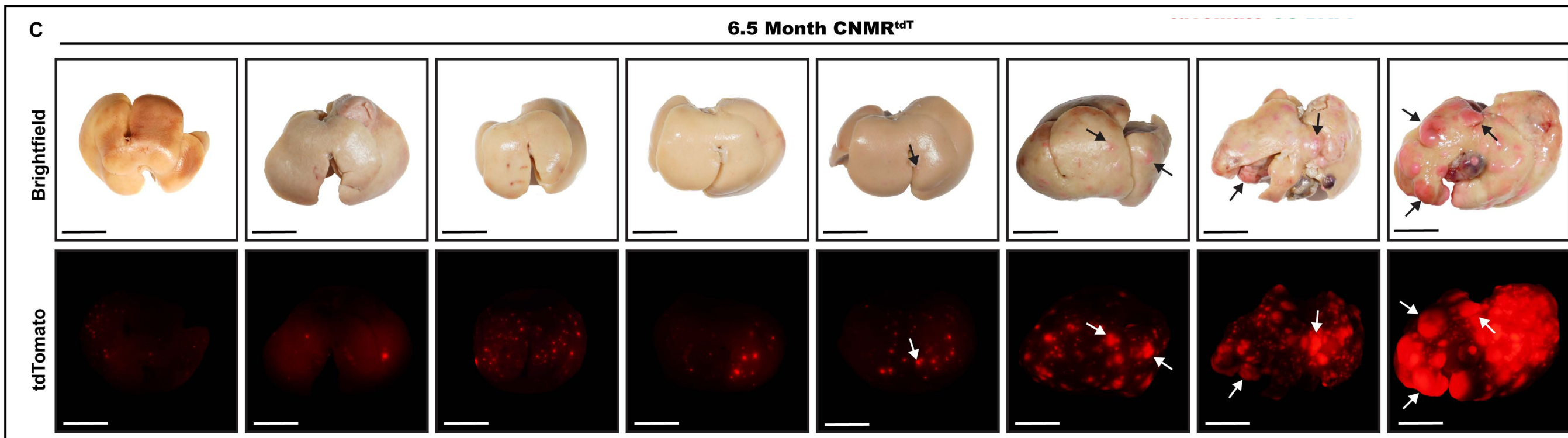

Fig. 6C. Brightfield and fluorescent macroscopic liver images from $CNMR^{tdT}$ mice at 6.5-month post-AAV injection. Arrows: representative tdT^+ macroscopic tumor. Scale = 1 cm.

The few $CNMR^{tdT}$ mice that developed tumor burdens comparable to CMR^{tdT} mice were found to contain recombination-escaping events by tumor genotyping and Western Blot (**Fig. S12E**). Since tumors with successful or incomplete recombination cannot be distinguished macroscopically, liver weight/body weight ratio or surface tumor quantification would not accurately reflect the contribution of NRF2 loss to *Ctnnb1*-mutant tumorigenesis. We have therefore decided to include the macroscopic overview together with tumor genotyping to provide the most accurate interpretation of the phenotype.

3. Recent studies have shown a difference between zone 1 and 3 mutant hepatocytes. Is the AAV leading to zone 3 mutants predominantly? Is there any difference in behavior of the zone 1 or 2 hepatocytes that have Ctnnb1 mutations?

Response to comment #3: We quantified the number of tdT$^+$;GS$^+$ hepatocytes per zone area in CR^{tdT} livers and found a preferential bias in the AAV infection rate in Zone 3 (**Fig. S1**). However, despite this initial zonal bias, longitudinal analysis revealed no significant differences in *Ctnnb1*-mutant hepatocyte attrition across zones over time.

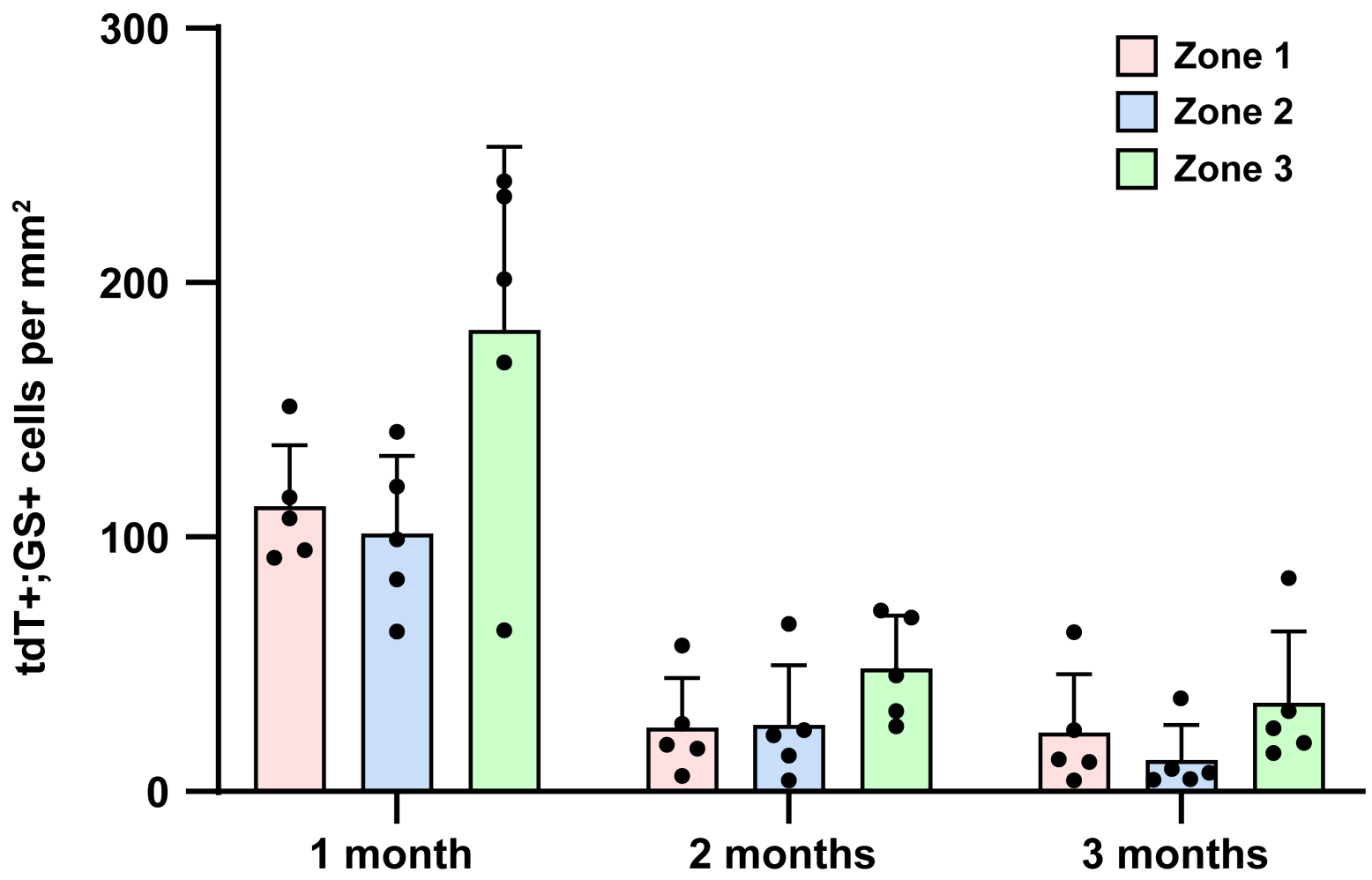

Fig. S1. β-catenin–mutant hepatocyte attrition is not affected by liver zonation. Quantification of the number of tdT$^+$;GS$^+$ hepatocytes per mm^2 within either Zone 1 (periportal; E-Cadherin$^+$), Zone 2 (E-Cadherin$^-$/GS$^-$), or Zone 3 (pericentral; GS$^+$) at the indicated timepoints post-AAV injection. Each point represents the mean count from a mouse. Each bar represents the average of the mice means ±. n=5 for each timepoint.

4. Can the authors confirm if the tumors that form are actually only Ctnnb1 mutant? What about other TSG or oncogenes?

Response to comment #4: This is a very important question to address, and we thank the Referee for bringing it up. We performed whole-exome sequencing on tumors isolated from 6.5-month CMR^{tdT} mice (**Fig. S4**). Across the nine tumors analyzed, a median of only 9 protein-altering variants were detected per tumor with no relevant recurrent mutations, including alterations in known TSGs or oncogenes. Only one tumor contained a missense mutation in *Arid1b.* These results further support that *Ctnnb1*-mutant hepatocytes in CMR^{tdT} mice can develop HCC without requiring cooperating mutations in other oncogenes or TSG.

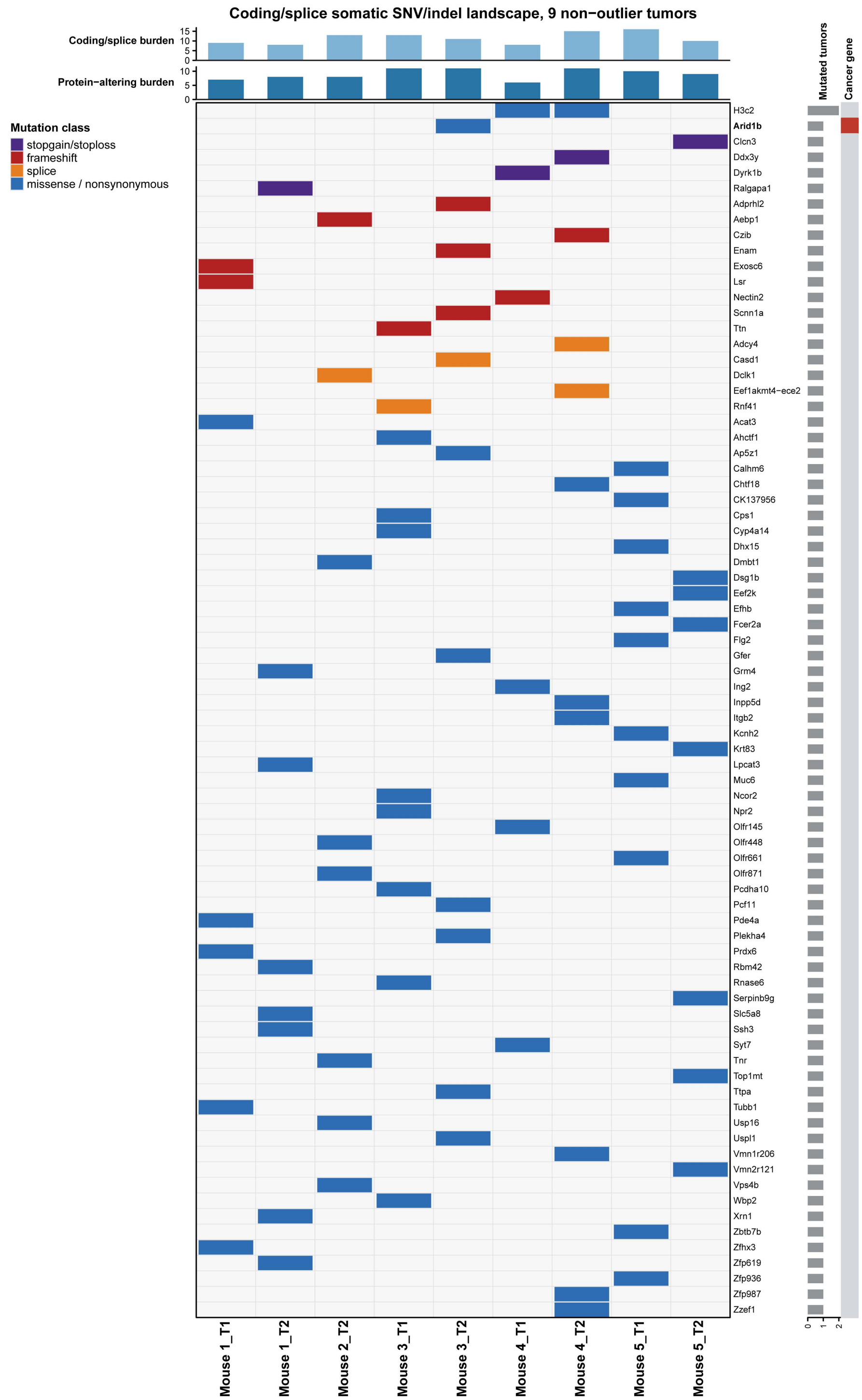

Fig. S4. No recurrent cooperation with additional oncogenic mutations on CMR^{tdT} tumors. Whole-exome sequencing was performed on tumors isolated from 6.5-month CMR^{tdT} mice. Two tumors (T1 and T2) were sequenced in five independent mice, and variants were identified by comparing with matched surrounding non-tumor liver tissue from the same mouse.

Minor comments

1. In Figure 4B, what is the frequency of Chop+ and Bip+ in the GS+/tdTomato double positive cells? This may focus on the Ctnnb1-mutant clones, as the GS-/tdTomato cells should experience relatively little ER stress compared to the GS+ cells.

Response to minor comment #1: Alongside our previous data quantifying the % CHOP$^+$ and BiP$^+$ in total tdT$^+$ cells in R^{tdT} and CR^{tdT} mice (**Fig. 4B**), we now include the quantification of CHOP$^+$ and BiP$^+$ frequency specifically within tdT$^+$;GS$^-$ and tdT$^+$;GS$^+$ hepatocytes in CR^{tdT} mice (**Fig. S7**).

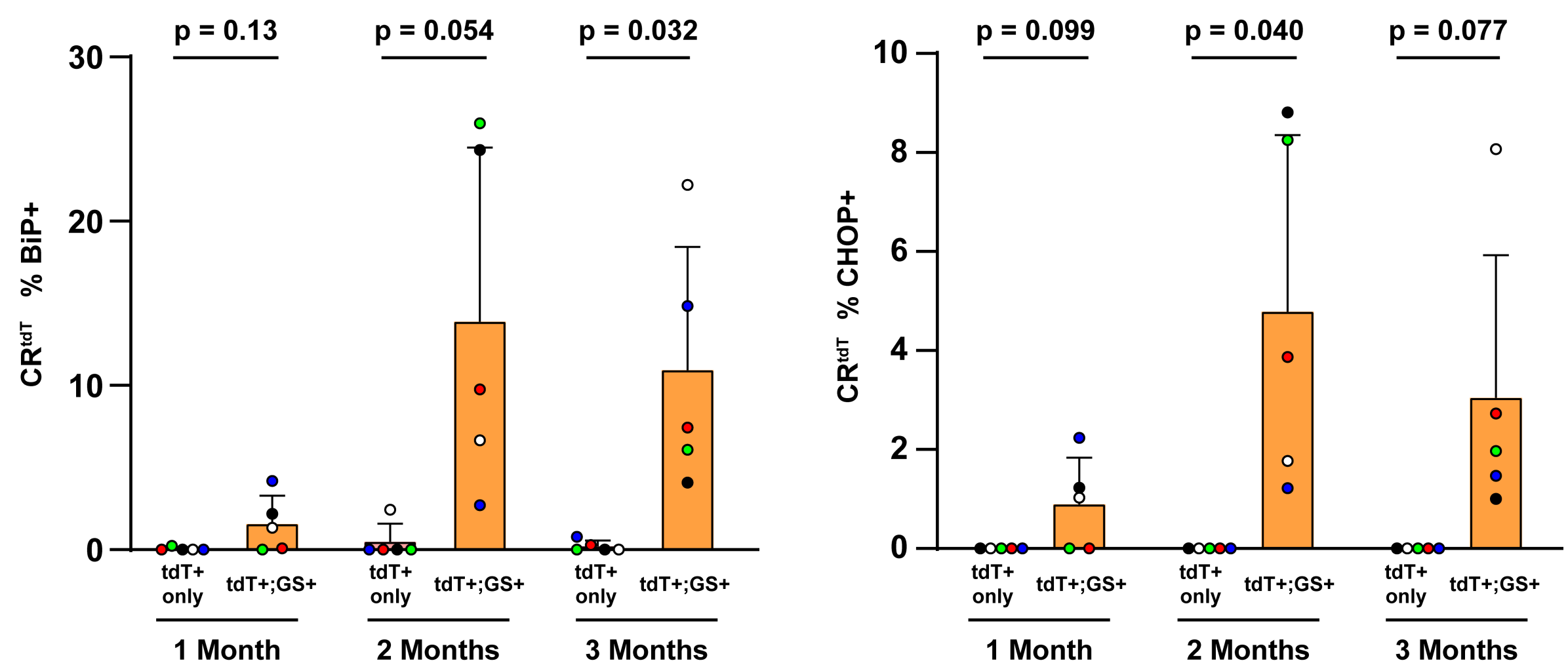

Fig. S7. CHOP and BiP are selectively elevated in *Ctnnb1^{ex3}* hepatocytes. Quantification of %tdT$^+$ and tdT$^+$;GS$^+$ cells that are also CHOP$^+$ or BiP$^+$ from CR^{tdT} samples in Fig. 1C-D. Each point represents the mean count for each mouse, and each pair of colored points at each timepoint correspond to the same mouse. Each bar represents the average of the mice means ±SD. Statistical significance was determined between tdT$^+$ and tdT$^+$;GS$^+$ groups at each timepoint by performing a paired, two-tailed t-test using the mean of the areas counted from each mouse. n=5.

2. In Figure 2, can you clarify the distinction between the single and multi clones and how they are evaluated differently?

Response to minor comment #2: In the CMR^{tdT} model, not all *Ctnnb1^{ex3}* hepatocytes underwent clonal expansion. Instead, many showed signs of cellular attrition as observed in the normal liver. Since these two populations represent two opposing biological outcomes (clonal expansion versus cellular attrition), statistical analyses combining them would be confounded. To address this, we analyzed single-cell and multicellular clones separately using empirically defined size thresholds.

To clarify this point, we edited the methods section as follows: "**For clone size analyses in Figure 2C,F, thresholds to distinguish single- vs. multi-cellular clones for each mouse were established empirically by determining the area distribution of individual *Ctnnb1^{ex3}* hepatocytes. The same mixed-effects analysis was then applied independently to single-cell and multicellular clones to test differences in phenotype above or below the threshold in CMR^{tdT} or aged CR^{tdT} mice within each population. The average threshold across mice for each genotype was plotted for each timepoint as a visual reference.**"

3. In Figure 6, what are the relative frequencies of larger NQO1+ tumors to smaller/middling NQO1- tumors in the CNMR model?

Response to minor comment #3: Of the eight 6.5-month CNMR^{tdT} liver samples, only three had large tumors (Please see response to comment #2b). Within these samples, we determined NQO1 positivity across tdT^{+};GS^{+} clones ranked by size. This analysis revealed a progressive enrichment of NQO1^{+} clones with increasing clone size, with the largest showing the highest frequency of NQO1 positivity (**Fig. S12C**).

C

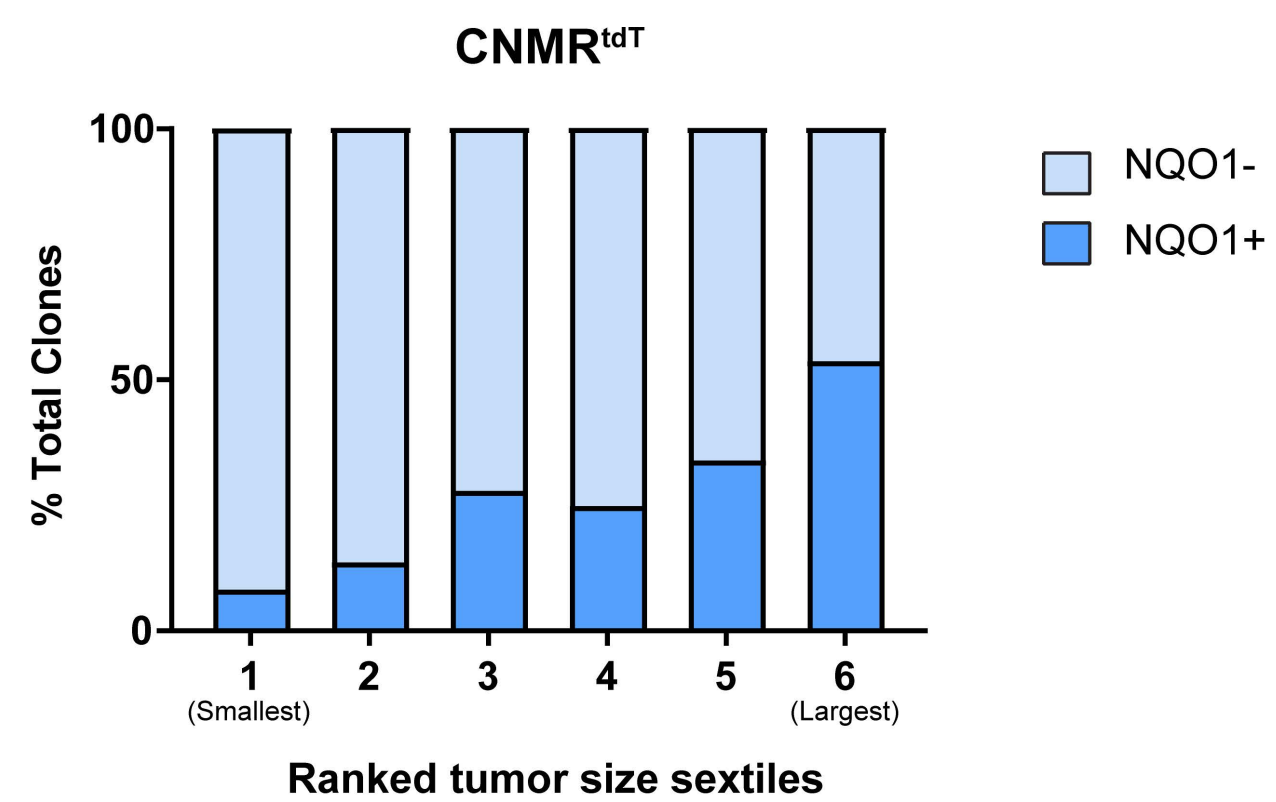

Fig. S12C. Quantification of % tdT^{+};GS^{+} clones in 6.5-month CNMR^{tdT} mice containing 5 cells or more that are either NQO1 positive or negative. Clones were divided into sextiles based on area. n= 3.

Referee #2:

In this study, the authors investigate how tissue integrity modulates the oncogenic potential of activating Ctnnb1 mutations in the liver. Using long-term clonal lineage tracing and single-cell RNA sequencing, they compare Ctnnb1 mutated hepatocytes in healthy, aged, and chronically injured livers. They show that in young, healthy tissue, mutant hepatocytes remain quiescent and are progressively eliminated, whereas in aged or chronically injured livers they undergo clonal expansion and form tumors without additional oncogenic events. Mechanistically, elimination in intact tissue is linked to stress-induced unfolded protein response (UPR) pathways, while expansion in diseased tissue is associated with AKT–NRF2 activation. The authors propose that tissue integrity shapes the fitness landscape of β-catenin–mutant cells. Overall, the study provides important mechanistic insight into the context-dependent mutagenic potential of CTNNB1 and significantly advances our understanding of how tissue state shapes β-catenin–driven hepatocarcinogenesis.

We thank the Referee for recognizing the importance of our work and for the constructive suggestions, which have helped to strengthen the manuscript and further support our conclusions.

1. Although comprehensive and supported by multiple genetic models, several aspects of the proposed model require clarification. The introduction states that β-catenin activation in healthy liver induces hepatomegaly (hyperplasia and hypertrophy) and death, yet the present data indicate that activation in a limited number of hepatocytes does not drive proliferation or clonal expansion. The discussion further notes that CTNNB1 mutations can lead to HCC in certain mouse models when a large fraction of hepatocytes is

mutated. However, CTNNB1-mutated HCC also occurs in human non-cirrhotic livers. Clarifying how these observations align with the current findings would strengthen the manuscript.

Response to comment #1: We thank the Referee for highlighting these points for clarification. To summarize our findings and discuss them in the context of previous literature, the Discussion has been modified as follows:

"This tissue state dependent fitness model reconciles previous reports demonstrating seemingly conflicting outcomes of oncogenic *Ctnnb1* activation in the normal liver depending on the number of hepatocytes recombined. Studies inducing oncogenic *Ctnnb1* in all hepatocytes reported hepatomegaly and liver failure. These phenotypes were attributed to widespread hepatocyte hypertrophy rather than proliferation, as well as extensive cell death[3,4], consistent with the initial hepatocyte hypertrophy and subsequent attrition and elimination observed in the long-term lineage tracing presented here. Similarly, the lack of tumorigenesis observed in studies using sparse recombination[3] is consistent with the finding that *Ctnnb1*-mutant hepatocytes are eliminated in the normal liver. In more recent work[5,6], the observation that tumors developed when the proportion of hepatocytes induced to carry the mutation was increased just below the threshold for acute lethality, supported the notion that β-catenin activating mutations have limited oncogenic potential in the liver. However, based on our model and data, the widespread loss of *Ctnnb1*-mutant hepatocytes predicted to occur in livers containing high numbers of mutant hepatocytes would trigger local tissue damage and regenerative responses, effectively mimicking features of chronic liver disease. Thus, the apparent weak oncogenic potential of β-catenin in this sub-lethal context of the normal liver can be reinterpreted as a consequence of altered tissue integrity and changes in the fitness landscape experienced by *Ctnnb1*-mutant hepatocytes. This negative selection model provides a potential explanation for the scarcity of *CTNNB1* mutations in the normal healthy human liver[7,8]."

2. The proposed mechanism for mutant hepatocyte attrition in healthy liver involves ROS-induced UPR activation. It would be important to clearly state whether an NRF2 response is detected in these cells, as suggested in Figure S4. Given that NFE2L2 is a β-catenin target gene (Gougelet et al., Hepatology 2014), its expression may be induced, but information regarding NRF2 stabilization and downstream target activation is needed.

Response to comment #2: To address this point, we performed a Western Blot on isolated tdT^+ hepatocytes from three independent mice for both R^{tdT} and CR^{tdT} genotypes at 1-month post-AAV injection (**Fig. S13E-F**).

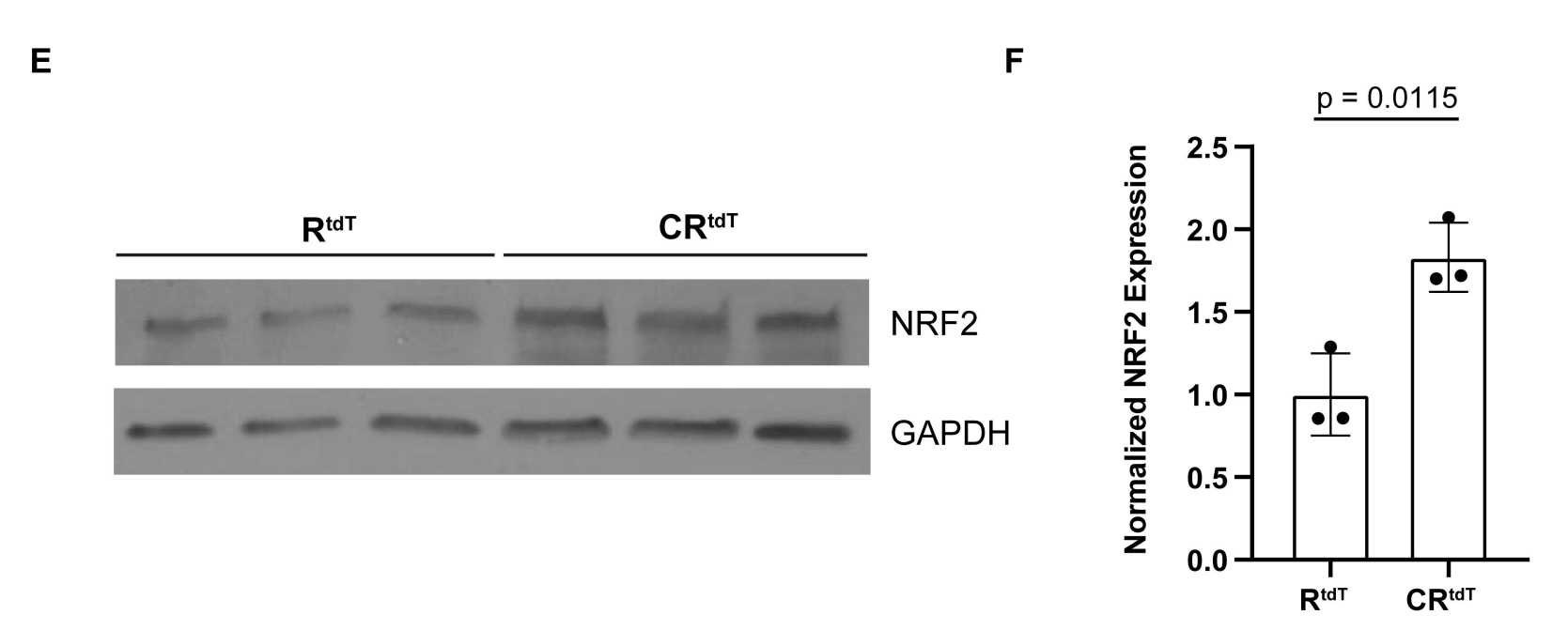

Fig. S13. E) Western blot of purified tdT^+ hepatocytes from R^{tdT} and CR^{tdT} mice 1 month post-AAV injection with the indicated antibodies. Each lane represents one mouse. **F)** Normalized NRF2 expression from the western blot in (E). Each dot represents one mouse, with black lines indicating mean ±SD. Statistical

As the referee predicted, NRF2 expression was increased in $Ctnnb1^{ex3}$ hepatocytes from CR^{tdT} mice compared to tdT^+ hepatocytes from R^{tdT} mice. This was accompanied by increased expression of the NRF2

target gene NQO1 (**Fig. S13D**), confirming activation of the NRF2 antioxidant response. However, despite NRF2 activation, *Ctnnb1*^{ex3} hepatocytes still have elevated ROS and undergo cellular attrition and elimination, indicating this compensatory response is insufficient to prevent elimination in the normal liver.

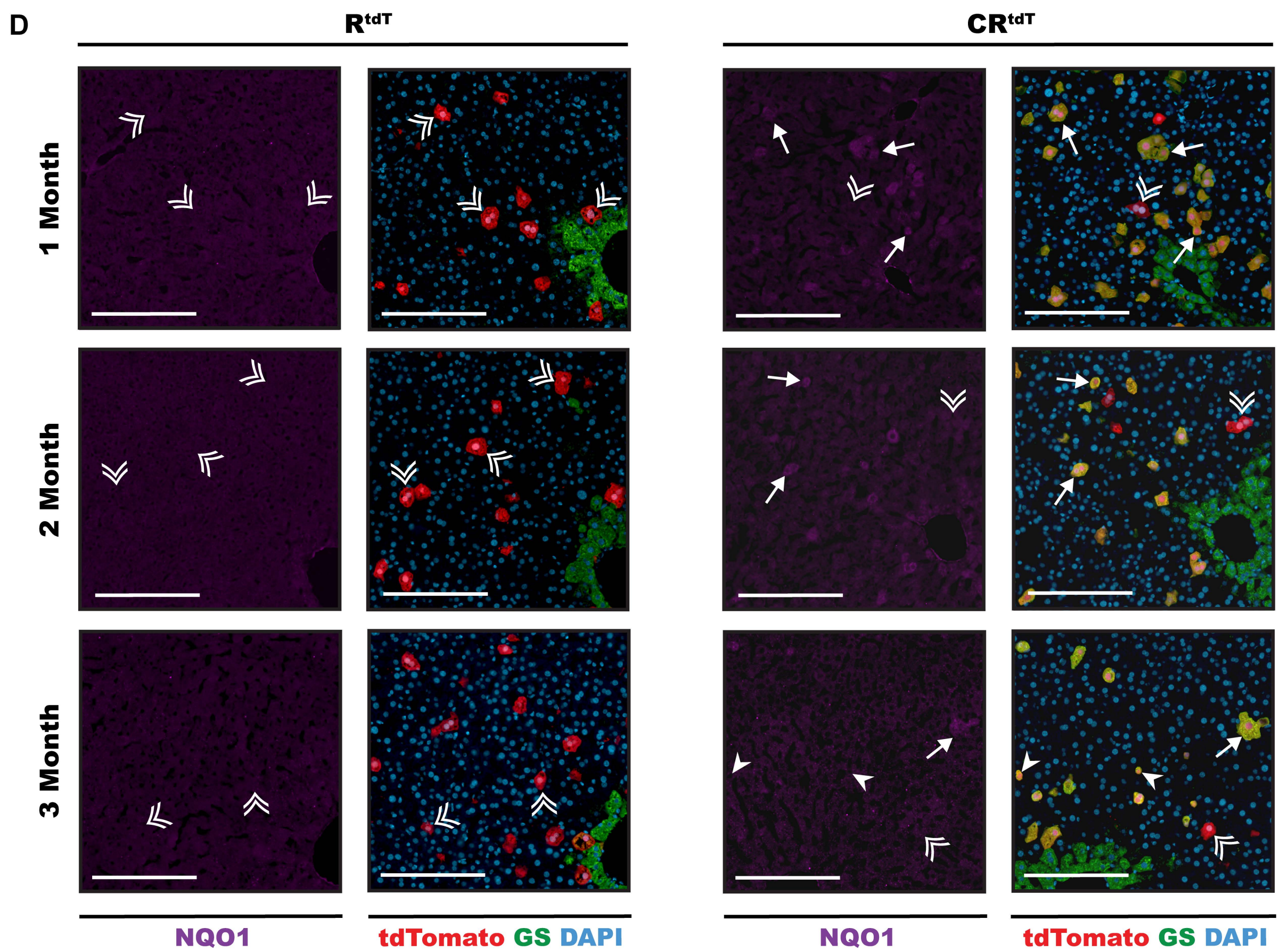

Fig. S13D. Representative immunofluorescence pictures of R^{tdT} and CR^{tdT} mice shown in Fig. 1B stained for tdT, GS, and NQO1. White arrows: normal size tdT^{+};GS^{+} cells. Arrowheads: small tdT^{+};GS^{+} cells. Double arrowheads: tdT^{+} only cells. Scale = 200 μm.

3. Moreover, while NAC treatment rescues mutant cell attrition, it remains unclear whether it resolves UPR activation and, more importantly, whether long-term ROS suppression would permit tumor development in an otherwise healthy liver.

<u>Response to comment #3:</u> To directly address whether NAC treatment resolves UPR activation, we quantified the percentage of CHOP^{+} and BiP^{+} *Ctnnb1*^{ex3} hepatocytes in NAC treated and untreated CR^{tdT} mice. Although NAC treatment showed a downward trend in BiP^{+} frequency, there was no significant decrease in the percentage of BiP+ or CHOP+ *Ctnnb1*^{ex3} hepatocytes compared to untreated mice (**Fig. S8**).

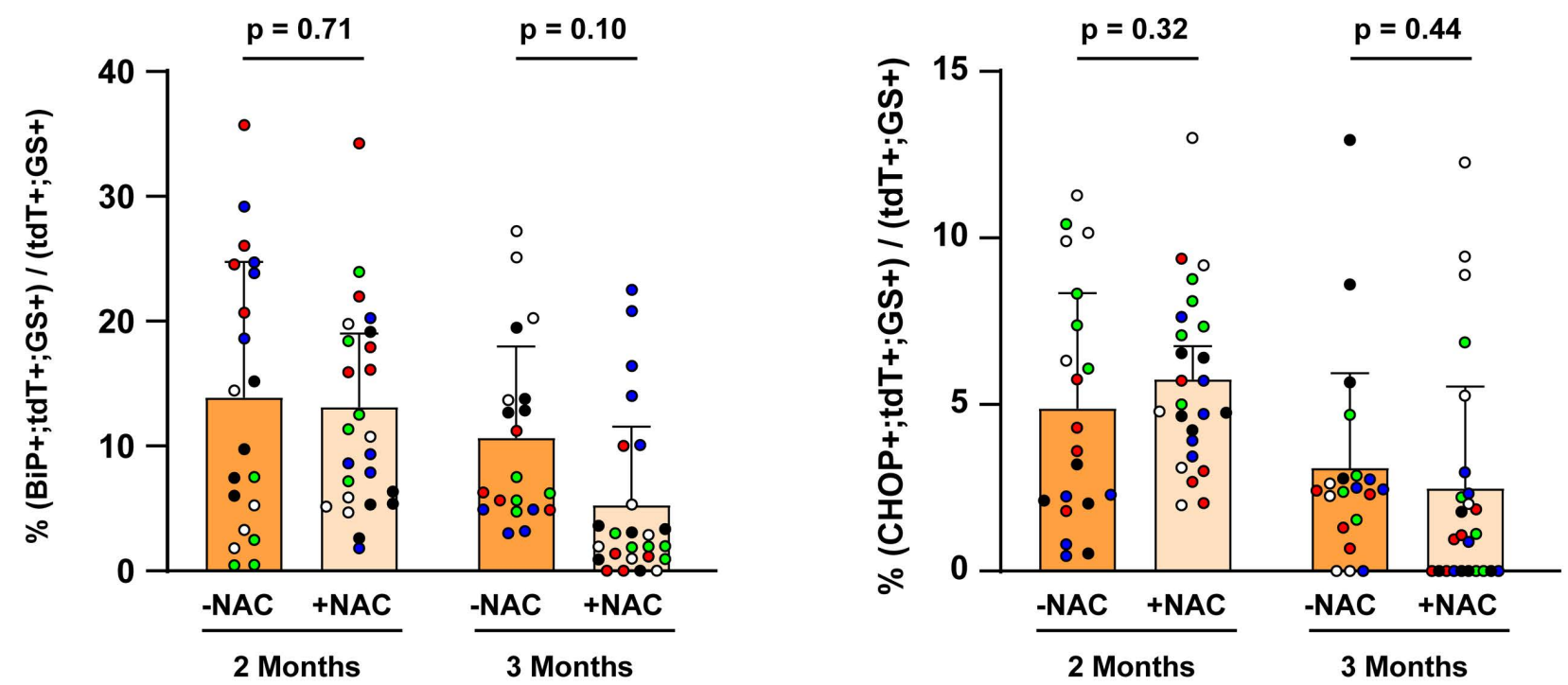

Fig. S8. NAC treatment does not significantly reduce UPR markers in *Ctnnb1*^{ex3} hepatocytes.

Quantification of %tdT^{+};GS^{+} cells that are also CHOP^{+} or BiP^{+} from NAC treated CR^{tdT} samples in Fig. 4E, and CR^{tdT} samples from Fig. 1C-D. Each point represents an area counted within a lobe, and each set of colored points within a treatment group correspond to the same mouse. Each bar represents the average of the mice means ±SD. Statistical significance was determined between -NAC and +NAC groups at each timepoint by performing an unpaired, two-tailed t-test using the mean of the areas counted from each mouse. n=5.

Consistent with this persistent stress response, our data showed that while NAC treatment normalized cellular size and attenuated cell loss, the number of *Ctnnb1*^{ex3} hepatocytes remained markedly lower than the number of tdT^{+} hepatocytes in R^{tdT} mice, and no clonal expansion was observed (**Fig. 4F and Fig. 1C**). Together, these results indicate that ROS suppression delays oncogenic β-catenin–induced cellular attrition but does not restore the positive fitness required for clonal expansion in the normal liver.

4. The induction of a SREBP-dependent lipogenic program in mutant hepatocytes within chronically injured liver is intriguing, through a potential AKT-mediated SREBP1 activation. However, it is not demonstrated whether this transcriptional program results in lipid accumulation; neutral lipid staining would help address this point.

Response to comment #4: We thank the referee for this insight. As suggested, we performed Oil Red O staining on 6.5-month CMR^{tdT} liver sections (**Fig. S11A**). The results show that tdT^{+} tumors do not accumulate neutral lipids and instead display reduced Oil Red O staining compared to the surrounding non-tumor tissue. Thus, although *Ctnnb1*^{ex3} tumor cells activate an SREBP-associated lipogenic transcriptional program, this is not translated in increased neutral lipid storage. Together with the sustained expression of lipogenic markers described in the response to comment #5, these findings suggest that lipid metabolism is rewired in β-catenin tumors in CMR^{tdT} mice to support the anabolic demands of tumor growth rather than lipid accumulation.

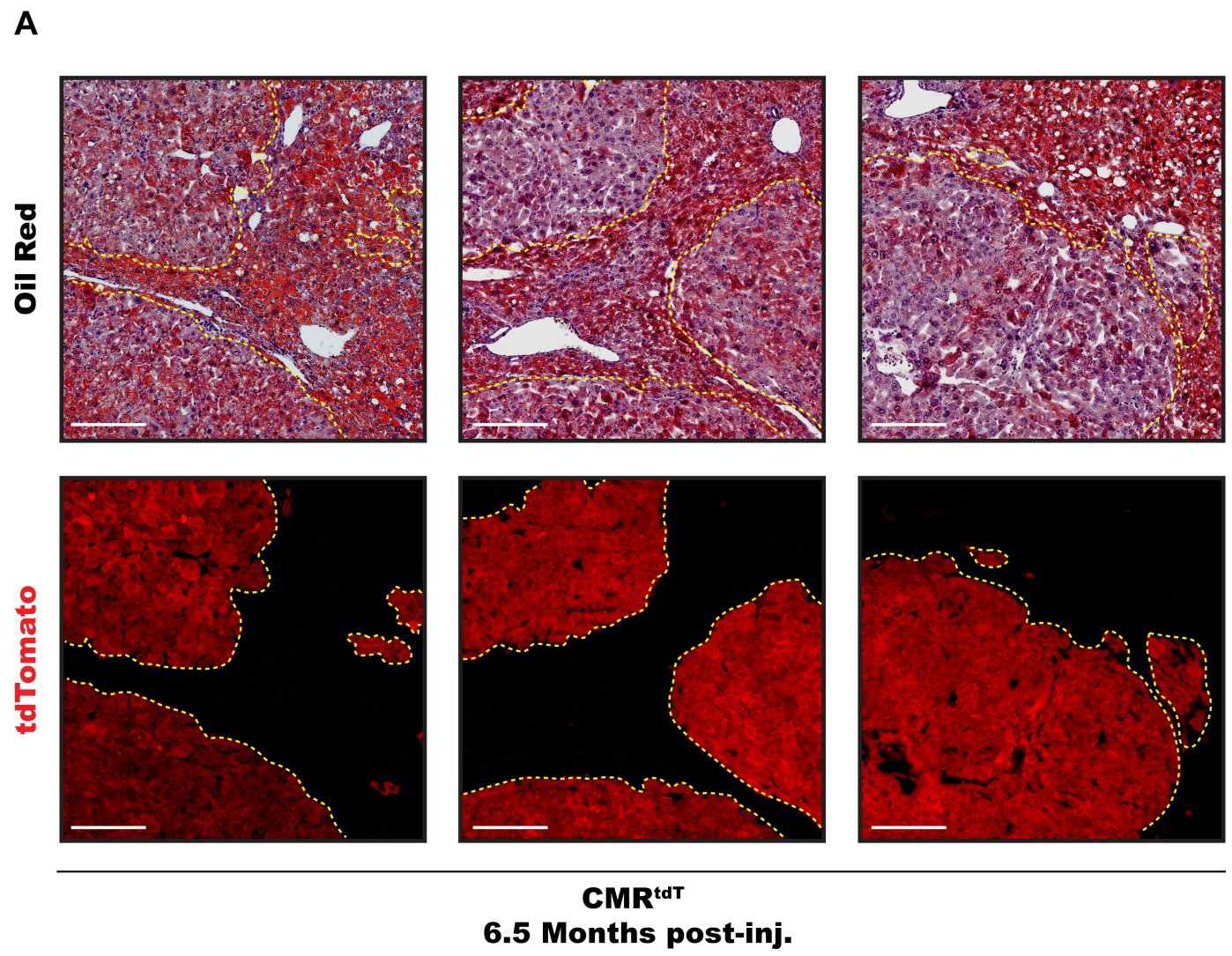

Fig. S11A. Representative Oil Red O staining and endogenous tdTomato fluorescence in three independent CMR^{tdT} liver tissue areas 6.5 months post-AAV injection. Yellow-dashed lines indicate tdT^{+} tumors. Scale = 200 µm.

5. In addition, human CTNNB1-mutated HCCs are rarely steatotic and typically display oxidative lipid metabolism, whereas AKT activation is more common in G1–G4 subclasses. G5-G6 classes display a lower proliferative rate than the other classes. It would therefore be informative to determine whether AKT signaling, lipogenesis, and NRF2 activation are maintained in tumors arising in the CMR model, or whether these early changes are transient.

Response to comment #5: We agree with the Referee that this is an important point. To determine whether AKT signaling, lipogenic pathway activation, and NRF2 activation are maintained in established tumors, we stained 6.5 month CMR^{tdT} samples for markers of AKT signaling (p-AKT (S473), p-AKT (T308), p-PRAS40), fatty acid synthesis (FASN, SCD1), and cholesterol synthesis (HMGCS1) (**Fig. S11B**). These markers were elevated in tdT^{+};GS^{+} tumors compared to surrounding non-tumor tissue, indicating that AKT signaling and lipogenic pathway upregulation are maintained in established tumors rather than being

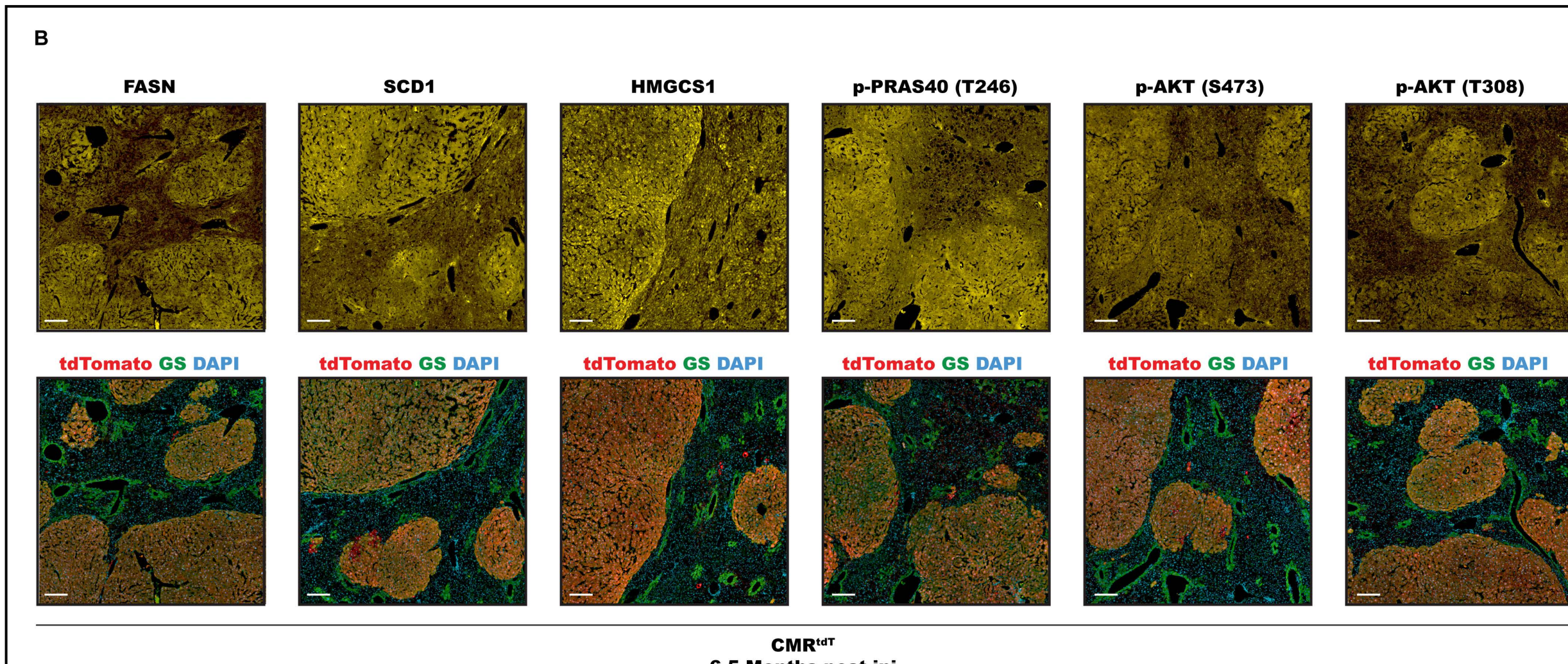

Fig. S11B. Representative immunofluorescence images of CMR^{tdT} liver 6.5 months post-AAV injection stained with tdT, GS, and either FASN, SCD1, HMGCS1, p-PRAS40 (T246), p-AKT (S473), or p-AKT (T308). Scale = 200 µm.

restricted to early tumorigenesis. Alongside the NQO1 positivity of these tumors (**Fig. S10G**) and the reduced Oil Red O staining described above (**Fig. S11A**), these findings suggest that β-catenin tumors in CMR^{tdT} mice maintain AKT-NRF2-programs while remaining non-steatotic. Thus, although these tumors do not accumulate neutral lipids, sustained lipogenic pathway upregulation may support anabolic demands of tumor growth rather than lipid storage, consistent with the non-steatotic histological presentation of human β-catenin mutated HCC noted by the Referee.

6. To further resolve the part played by AKT activation in maintaining the mutated hepatocytes, the authors used a constitutively active AKT in their model, showing that it is sufficient to maintain CTNNB1 mutated hepatocytes in a sane liver allowing their rapid clonal expansion but a clonal expansion is also observed in the wild type CTNNB1 hepatocytes in those livers. How is it possible under these condition to separate the pro-proliferative aspect of AKT hyperactivation in their model to a specific function in the mutated hepatocytes?

Response to comment #6: We appreciate the opportunity to clarify this point. We agree that, as observed in R^{tdT} mice, myr-AKT alone has a mild pro-proliferative effect in hepatocytes, and therefore the comparison with hepatocytes expressing both myr-AKT and oncogenic β-Catenin is important. In the original manuscript, this comparison showed qualitatively that myr-AKT plus oncogenic β-Catenin produced substantially greater clonal expansion than myr-AKT alone, while oncogenic β-Catenin alone undergoes negative selection in the normal liver. In the revised manuscript, we now include Ki67 staining, which quantitatively confirms that hepatocytes expressing both myr-AKT and oncogenic β-Catenin have a significantly higher proliferative rate than hepatocytes expressing myr-AKT alone (**Fig. 7E,H**).

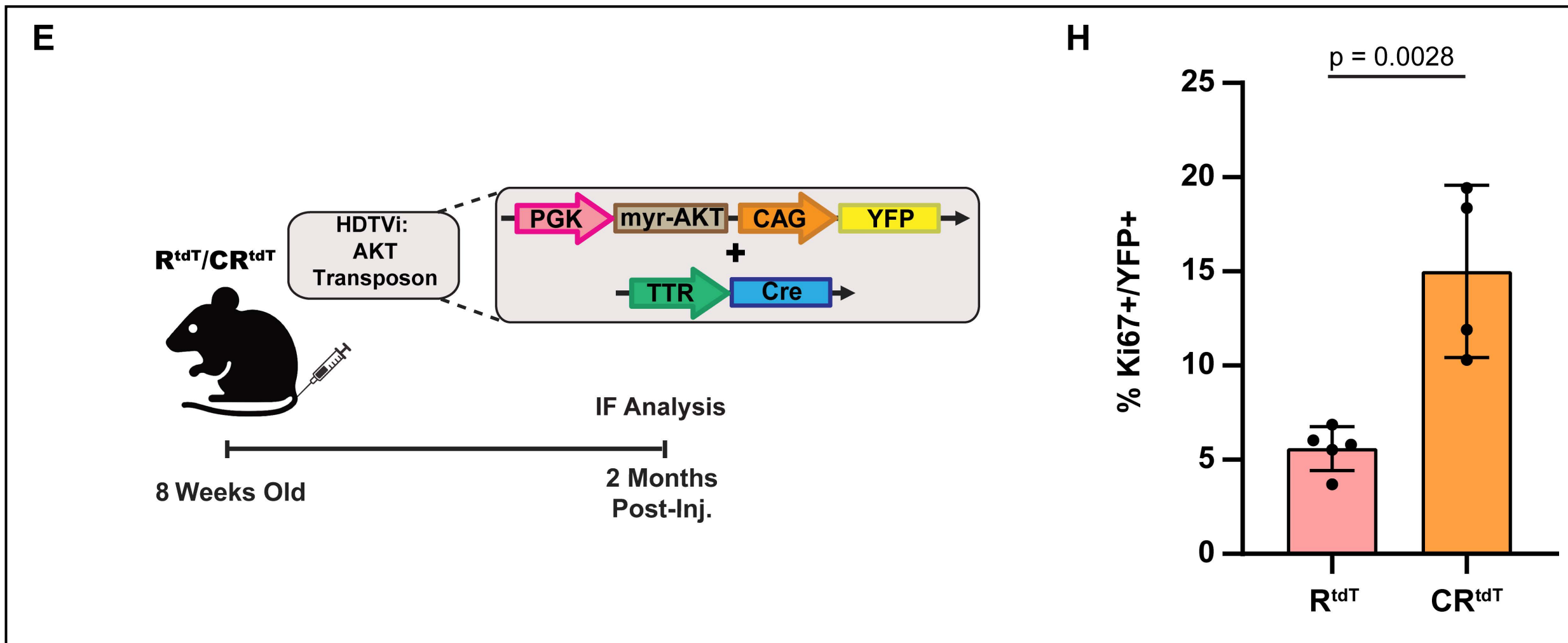

Fig. 7. E) Diagram depicting the experimental strategy to express myristoylated AKT in Ctnnb1ex3 hepatocytes in the context of the normal liver. **H)** Quantification of $\%YFP^{+}$ cells that are also $Ki67^{+}$ from samples described in (F). Each point represents the average $\%Ki67^{+}$ from a mouse. Each bar represents the average of the mice ±SD. Statistical significance was determined between R^{tdT} and CR^{tdT} groups by performing an unpaired, two-tailed t-test. n=5, 4 for R^{tdT} and CR^{tdT}, respectively.

Collectively, these data support that AKT activation does not simply provide a generic or additive proliferative stimulus, but instead reverses the attrition and elimination of *Ctnnb1*^{ex3} hepatocytes, converting them into a population that is even more proliferative than myr-AKT expressing hepatocytes.

[Editorial note: panel e created in BioRender. Hewett, C. (2026) https://BioRender.com/t3qttns.]

Several minor points should also be addressed. (i) The nutritional status of mice at sacrifice (fed, refed, or fasted) is not specified, as metabolic pathways are highly nutrition-sensitive.

Response to minor comment #1: We have now included the nutritional status of the mice at sacrifice in the methods section as follows: "**Unless otherwise specified, animals were kept on standard laboratory chow and drinking water ad libitum in a temperature and humidity-controlled, 12-hour light cycle facility. Food was removed for 3-4 hours before sacrifice.**"

(ii) Liver zonation should be clearly defined (zone 1 periportal; zone 3 pericentral/perivenous), particularly given its regulation by β-catenin.

Response to minor comment #2: We have edited the text to define the different zones found in the liver as follows:

"…**we used a GS specific antibody to identify endogenous pericentral zone 3 hepatocytes**"

"…**along with typical periportal zone 1 markers**"

(iii) The statement that CTNNB1 mutations frequently co-occur with KEAP1 or NFE2L2 mutations should be reconsidered, as such associations are not statistically true (Savall et al., Hepatology Communications 2021).

Response to minor comment #3: We appreciate the comment, and we agree that the original wording could be interpreted as implying a consistent statistical co-occurrence between *CTNNB1* mutations and *NFE2L2/KEAP1* alterations across HCC patient cohorts. As noted by the Referee, this association is not consistently observed. Therefore, to avoid overstating this point, we edited the text to focus on the potential functional advantage of NRF2 pathway activation in CTNNB1-mutant hepatocytes:

"**This switch provides a mechanistic explanation for why co-mutations of *NFE2L2* or *KEAP1* with *CTNNB1* in human HCC can be advantageous**…"

(iv) The authors suggest that lipogenesis may confer a selective advantage to transformed cells; further clarification of this point would be helpful.

Response to minor comment #4: We apologize for the confusion. We have edited the text to clarify that increased lipogenesis alone does not confer a selective advantage but is part of the broader AKT-associated metabolic rewiring acquired by expanding *Ctnnb1*^{ex3} clones in chronic liver disease. The revised text now reads:

"**In chronic liver disease, this regulatory switch flips and activates AKT in a subset of *Ctnnb1*^{ex3} hepatocytes. These hepatocytes acquire elevated lipogenic and antioxidant programs, directly inhibiting GSK3β while promoting SREBF-mediated lipogenesis. Overall, this AKT activation mitigates cellular stress and increases survival.**"

Referee #3:

This study by Illendula et al. features many elegant reporter and lineage-tracing studies and introduces a potential new paradigm of tumor development dependent on mutational fitness. However, there were significant concerns with experimental design and data interpretation.

We thank the Referee for the positive assessment of our study and for recognizing the potential significance of the conceptual advance. We also appreciate the constructive comments, which have helped us clarify key aspects of the experimental design, data interpretation, and conclusions.

1. The authors show that a subset of hepatocytes adopt an AKT-NRF2 permissive signaling state to unleash beta-catenin oncogenicity, but only allude to disease etiology, local tissue degeneration, and

specific signaling pathways being engaged as possible reasons why only some hepatocytes develop this phenotype. Further studies are needed to describe the mechanisms of this phenomenon. For example, is glutathione metabolism different in these cells?

Response to comment #1: To further characterize the AKT-NRF2 permissive state, and specifically address the Referee's suggestion regarding glutathione metabolism, we analyzed glutathione-related gene modules in our CMR^{tdT} scRNAseq dataset. We compared gene sets related to glutathione synthesis, recycling, and conjugation across cell clusters (**Fig. S10E**). This analysis showed that the Antioxidant (NRF2) cluster displays strong upregulation of all three glutathione metabolism modules compared to normal hepatocytes, as well as Bridge, UPR, and Late clusters. These results further support that this subset of *Ctnnb1^{ex3}* hepatocytes activates a robust NRF2-associated antioxidant program.

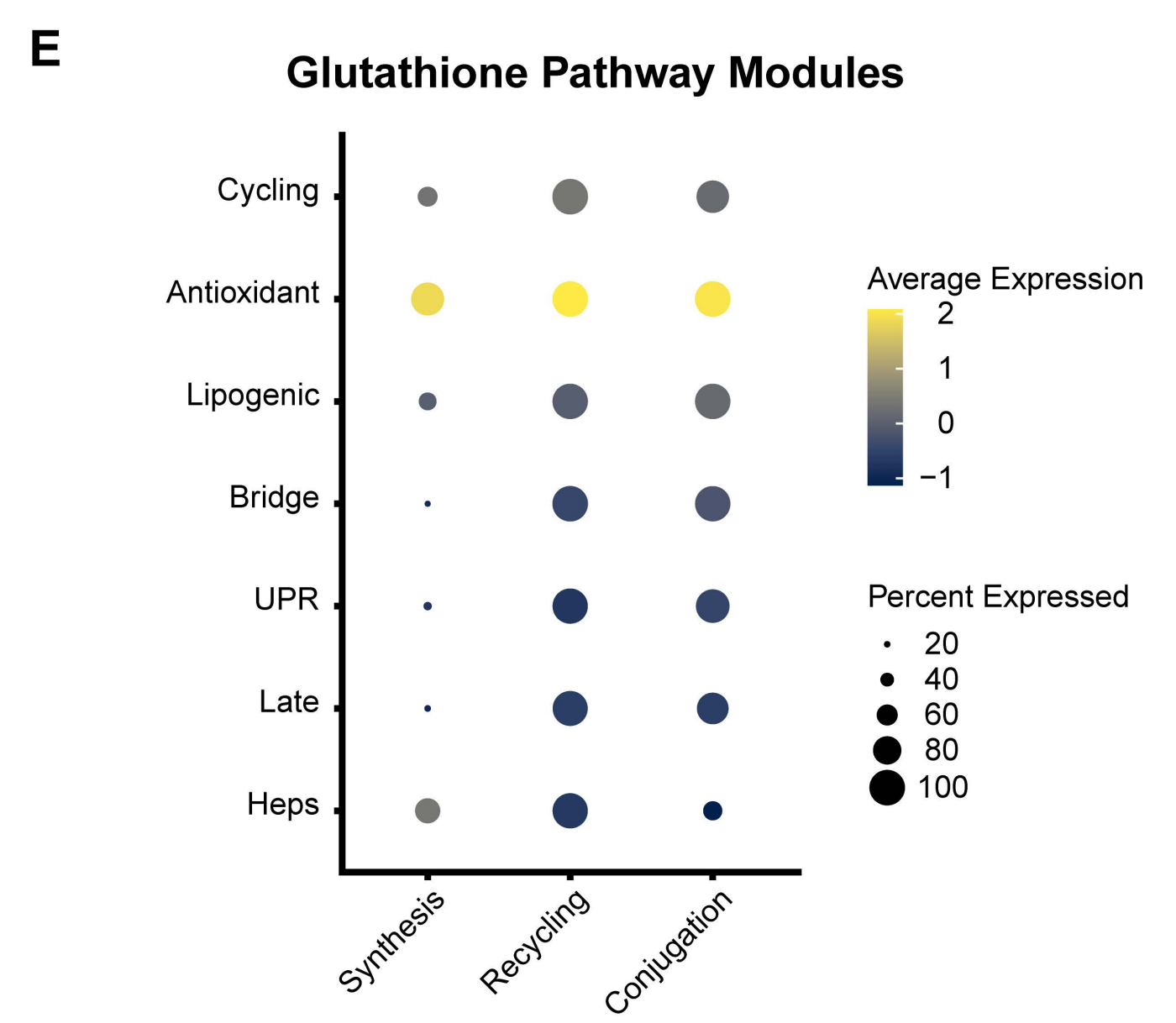

Fig. S10E. Analysis of glutathione metabolism in *Ctnnb1^{ex3}* hepatocytes.

2. The beta-catenin mutated tumors do not replicate patient subtypes, since beta-catenin mutations occur concomitantly with other aberrations. Authors should demonstrate similarity to patient HCC subsets.

Response to comment #2: We thank the Referee for this suggestion and agree that this is an important consideration. To address this point, we performed RNA-seq on CMR^{tdT} tumors and integrated this new dataset with human HCC transcriptomic data from TCGA using our previously published comparative transcriptomic analysis pipeline[9] (**Fig. S5**). For clarity in the reviewer response, CMR^{tdT} tumors are highlighted with a pink box, and the corresponding human *CTNNB1*-mutated HCC subsets are highlighted with black boxes. This analysis showed that CMR^{tdT} tumors most closely resemble a subset of human HCCs harboring *CTNNB1* mutations. Notably, these *CTNNB1*-mutated human HCCs also showed high similarity to STAM mouse model tumors that were independently found to harbor *Ctnnb1* mutations. Overall, this integrative analysis indicates that *Ctnnb1*-mutated tumors from CMR^{tdT} mice do not align randomly across human HCCs, but instead transcriptionally resemble a patient subset defined by *CTNNB1* mutations. Thus, despite the additional genetic complexity present in human HCC, tumors from CMR^{tdT} mice share core transcriptional features of human *CTNNB1*-mutated HCC.

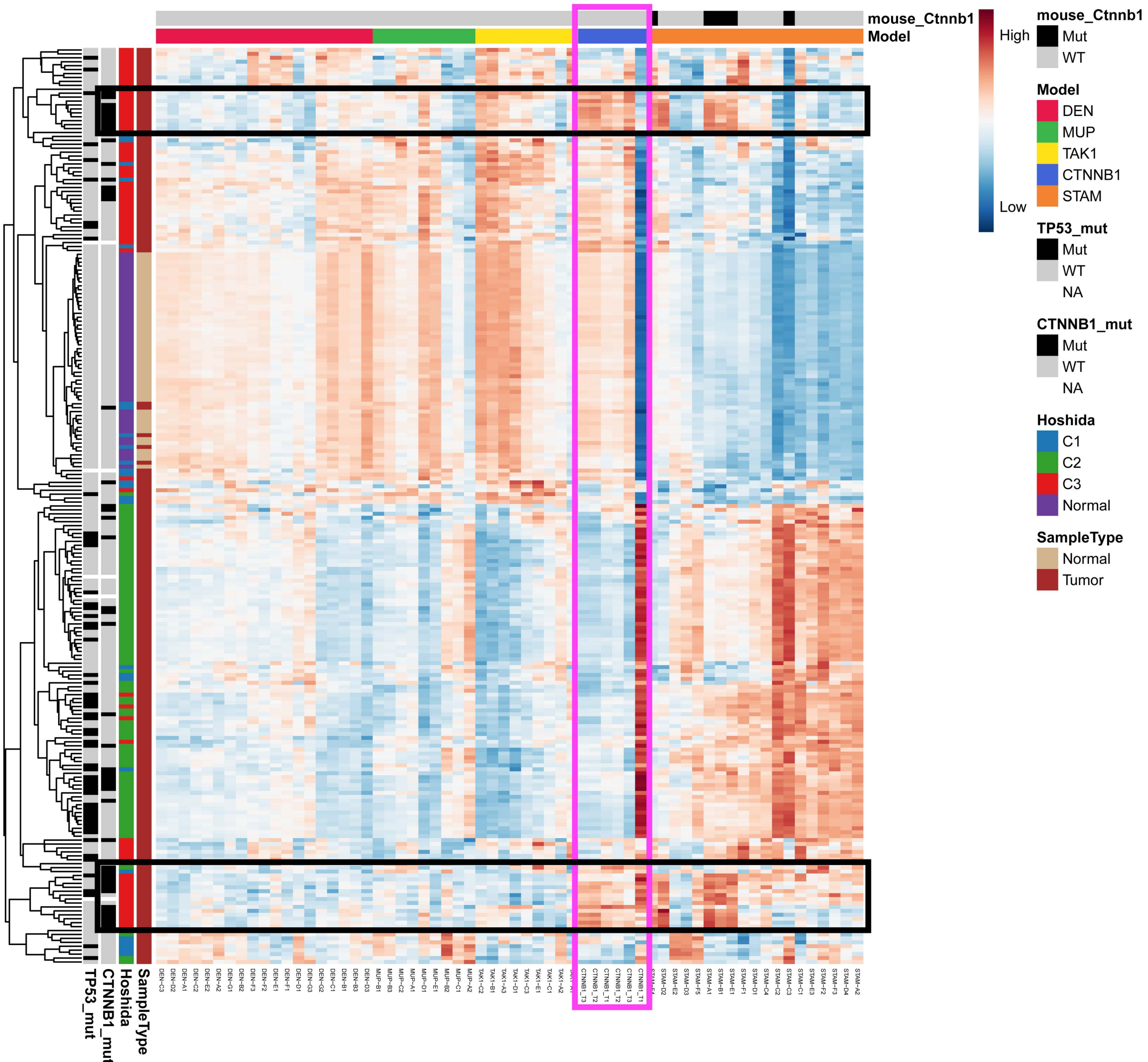

Fig. S5. Gene-expression profile correlations between mouse tumors and human HCCs. Pairwise comparisons of mouse tumors grouped by model (columns) and human HCC samples (rows). *Ctnnb1* mutational status is listed for all mouse tumors on top. *CTNNB1* and *TP53* mutational status is listed for all human HCC tumors on the left. Human sample type and Hoshida clustering assignments of human HCCs are listed on the left. Human HCCs were clustered based on pairwise similarities with mouse tumors using 800 HCC-related gene-expression levels. Red shading indicates high gene-expression correlation between mouse and human tumors, whereas blue shading indicates low correlation.

3. The coactivation of mutated beta-catenin and NRF2 has been shown previously (PMID: 33529367); thus, this finding is not particularly novel.

Response to comment #3: We agree with the Referee that previous studies have shown cooperation between oncogenic β-Catenin and NRF2 activation in HCC. However, our study provides several new insights into how this interaction is regulated by tissue context and pathway strength.

First, we show that in the normal liver, *Ctnnb1*^{ex3} hepatocytes induce endogenous NRF2 activity, but this response is insufficient to overcome the maladaptive consequences of oncogenic β-catenin activation. Second, we show that simple overexpression of NRF2 is also insufficient to drive robust clonal expansion

or tumorigenesis, whereas constitutively active NRF2 is sufficient to promote expansion in the normal liver. Third, we show that that although constitutively active AKT induces strong NRF2 pathway activation (high NQO1 expression), NRF2 is not required for AKT-mediated reversal of Ctnnb1-mutant hepatocyte attrition and clonal expansion in the normal liver. Finally, in chronic liver disease, NRF2 contributes to tumorigenesis, but NRF2 loss does not fully prevent clonal expansions, although they are strongly delayed. Together, these findings extend previous work by defining how endogenous NRF2 activity, oncogenic NRF2 activation, AKT signaling, and chronic liver injury differentially regulate the fitness of Ctnnb1-mutant hepatocytes.

4. The authors demonstrate that AKT-NRF2 activation mediates beta-catenin-driven tumorigenesis. In addition to NRF2, beta-catenin interacts with Lkb1, Kras, and C-met, and these interactions can drive oncogenesis. Did the authors evaluate whether these were altered during hepatocyte expansion as tissue integrity deteriorated? The authors should also discuss whether these may also allow a permissive state for beta-catenin oncogenicity.

Response to comment #4: We agree with the Referee that Lkb1, Kras, and c-Met are important cooperating players to consider based on previously published studies. Our study does not exclude the possibility that these or other oncogenic alterations can create permissive states for β-Catenin oncogenicity. In the present study, our analyses focused on AKT-NRF2 because this axis emerged from our single-cell and validation analyses as a dominant feature of expanding *Ctnnb1*-mutant hepatocytes in chronically injured liver.

To determine whether established tumors in CMR^{tdT} mice contained additional cooperating oncogenic or tumor suppressor alterations, we performed whole-exome sequencing on these tumors (**Fig. S4).** We did not detect mutations in Lkb1, Kras, or c-Met, nor did we identify recurrent alterations in other known oncogenes or tumor suppressor genes. These results indicate that, in CMR^{tdT} mice, $Ctnnb1^{ex3}$ hepatocytes can expand and form tumors without genetic alterations in Lkb1, Kras, or c-Met.

We have revised the discussion to include: "**While additional pathways induced from chronic injury may also cooperate with oncogenic β-catenin, these findings identify the AKT-NRF2 axis as a dominant molecular gate that determines whether β-catenin mutated hepatocytes are positively or negatively selected.**"

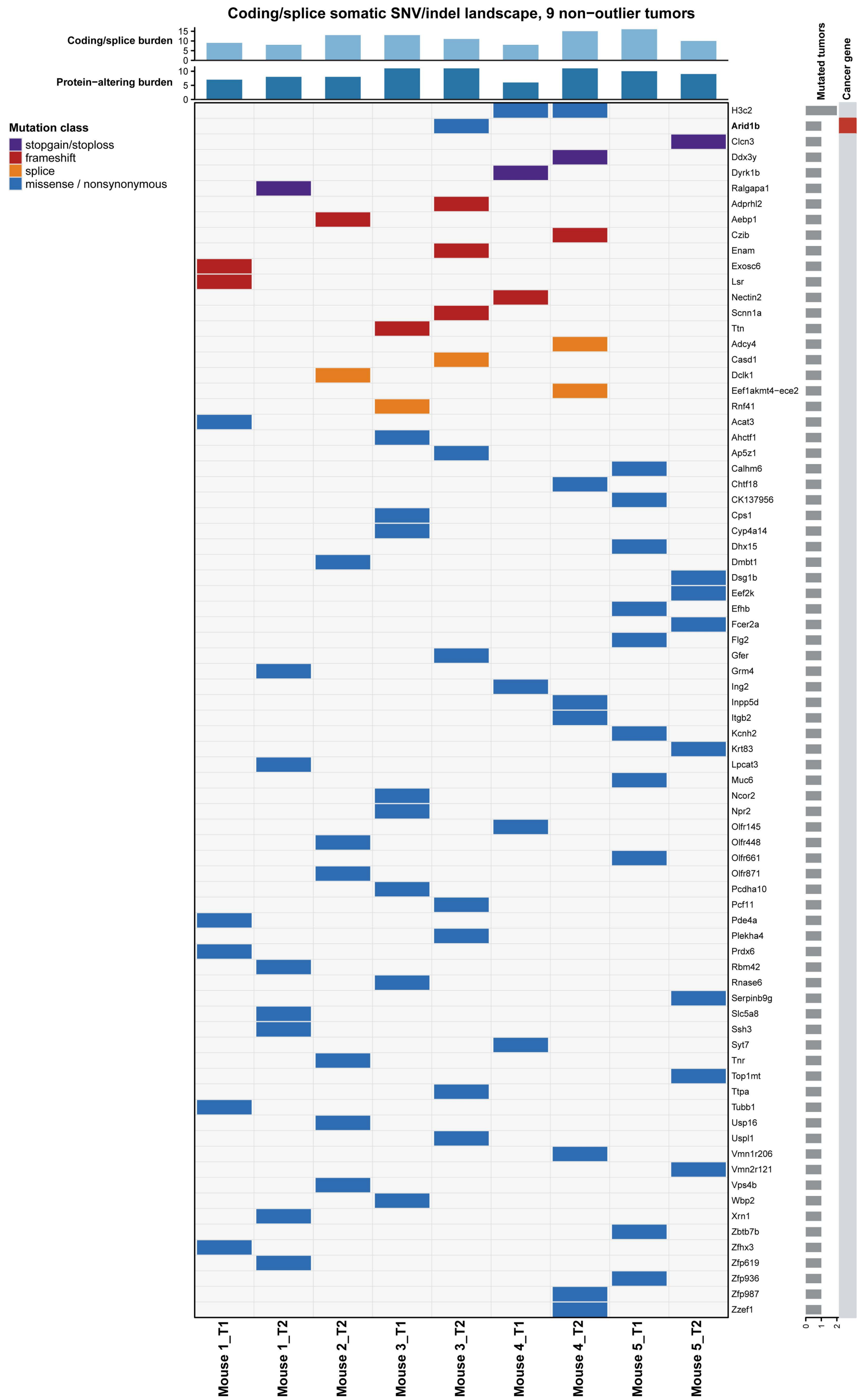

Fig. S4. No recurrent cooperation with additional oncogenic mutations on CMR^{tdT} tumors. Whole-exome sequencing was performed on tumors isolated from 6.5-month CMR^{tdT} mice. Two tumors (T1 and T2) were sequenced in five independent mice, and variants were identified by comparing with matched surrounding non-tumor liver tissue from the same mouse.

5a. Proliferation is a hallmark of beta-catenin mutated hepatocytes. Can the authors explain why proliferating Ctnnb1ex3 mutated hepatocytes were not present in the normal livers?

Response to comment #5a: We appreciate the opportunity to clarify this point since it is central to the manuscript. Our data indicate that proliferation is not an intrinsic or universal consequence of oncogenic β-Catenin activation in hepatocytes. Rather, the fate of *Ctnnb1*^{ex3} hepatocytes is strongly context dependent. In the normal liver, *Ctnnb1*-mutated hepatocytes do not clonally expand or show increased proliferation. Instead, oncogenic β-catenin activation induces an anabolic surge with cellular hypertrophy, ROS accumulation, ER-stress/UPR activation, leading to progressive cellular attrition and eventual elimination (**Fig. 1, 3, 4**). In contrast, during chronic liver disease or aging, *Ctnnb1*^{ex3} hepatocytes acquire positive fitness and proliferative capacity. Therefore, the absence of proliferating *Ctnnb1*^{ex3} hepatocytes in the normal liver is the result of the negative selection pressure imposed by the intact healthy liver microenvironment.

5b. Additionally, proliferation should be measured directly instead of through pathway analysis and size of tdTomato clusters.

Response to comment #5b: We now provide a direct measurement of proliferation by quantifying Ki67 positivity in tdT^{+};GS^{+} hepatocytes in CR^{tdT} and CMR^{tdT} mice (**Fig. S2**). In the normal liver, Ki67 positivity in *Ctnnb1*^{ex3} hepatocytes progressively declines towards zero over time. In contrast, in the context of chronic liver disease, Ki67 positivity increases over time. These data directly confirm that *Ctnnb1*^{ex3} hepatocytes do not proliferate in the normal liver but acquire proliferative capacity in the damaged liver, consistent with the clonal expansion observed by lineage tracing.

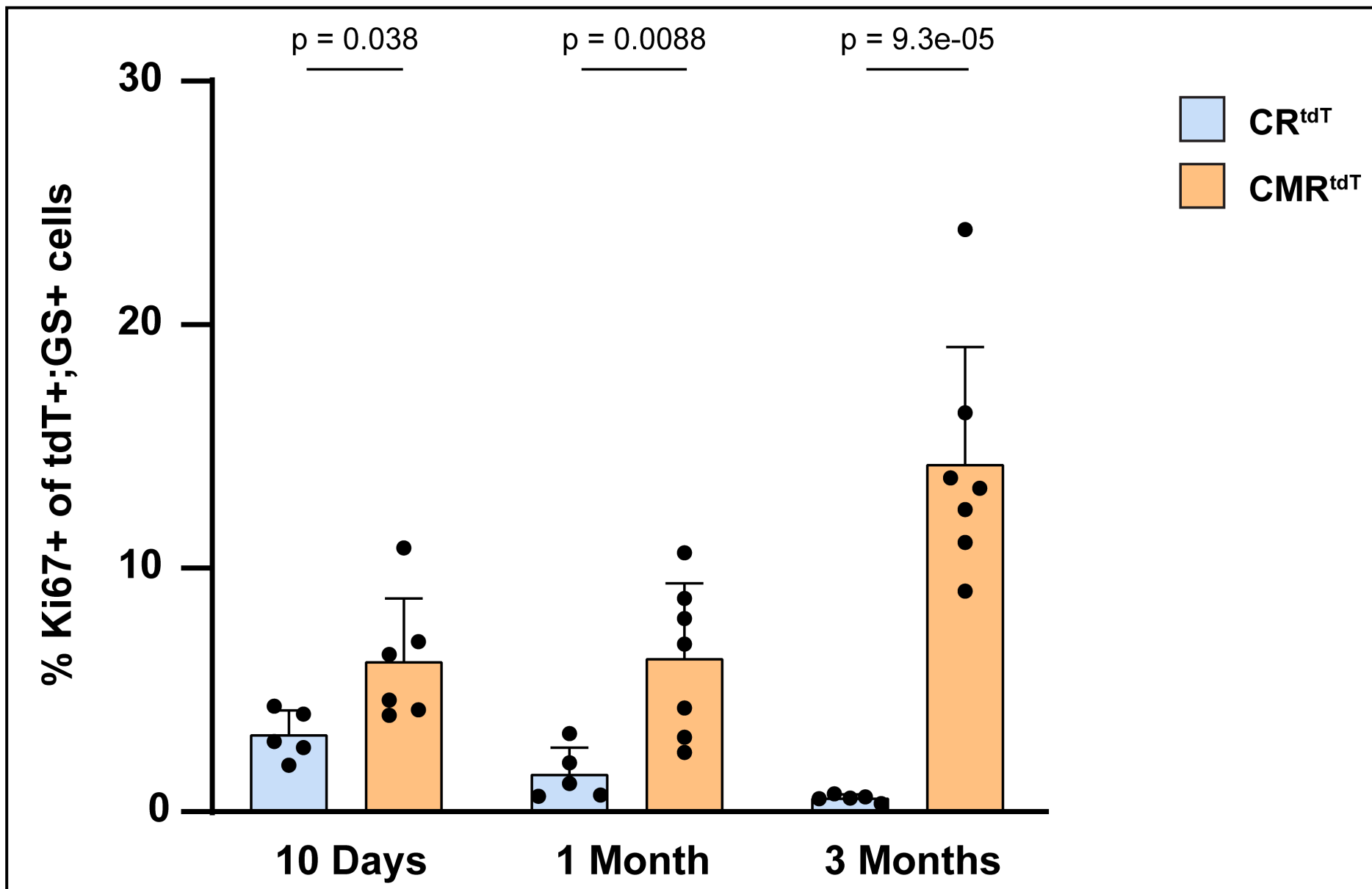

Fig. S2. *Ctnnb1*^{ex3} hepatocytes proliferate in the damaged liver but not in the normal liver. Quantification of % tdT^{+};GS^{+} cells that are also Ki67^{+} from the same samples described in (Fig. 1C and 2C) at the indicated timepoints. Each point represents the mean percentage from each mouse. Each bar represents the mean of per-mouse values ±SD. Statistical significance was determined between CR^{tdT} and CMR^{tdT} groups by performing an unpaired, two-tailed t-test using the mice means. n=5 for all CR^{tdT} conditions, n=6, 7, 7 for 10 days, 1 month, and 3 months CMR^{tdT}, respectively.

6. Constitutive activation of beta-catenin at early ages leads to oncogenesis as aging and chronic injury progress. However, would the authors expect similar changes in hepatocyte fitness and pathways if the beta-catenin mutation were introduced after the animals had aged and chronic injury had progressed?

Response to comment #6: We thank the Referee for highlighting this point of ambiguity. In both experimental settings, *Ctnnb1* mutations were induced after the relevant tissue context had already been established: in CMR^{tdT} mice, recombination was performed after mice had been undergoing liver injury for 5 weeks. In the aging experiment, recombination was performed after mice had reached 15 months of age. To clarify the experimental design, we have edited the schematic corresponding to the aged mice experiment (**Fig. 2E**). Importantly, because *Ctnnb1*-mutant hepatocytes are eliminated in the young normal liver (**Fig.1**), clonal expansion in aged mice would not be expected if recombination had been performed at young age.

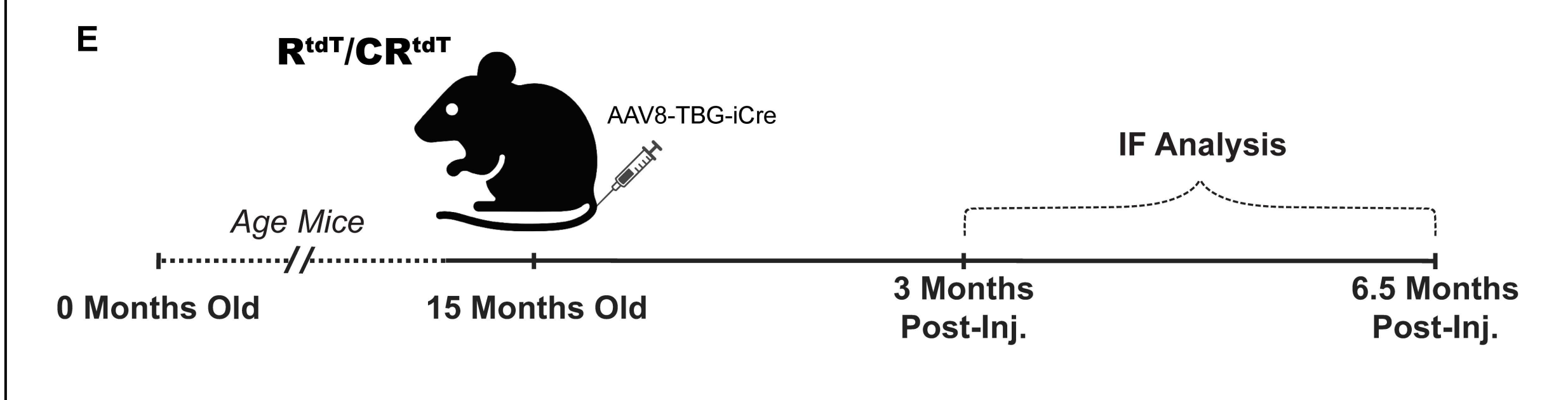

Fig. 2E. Schematic of the clonal lineage tracing system in the context of aged R^{tdT} or CR^{tdT} mice.

[Editorial note: panel created in BioRender. Hewett, C. (2026) https://BioRender.com/t3qttns.]

7. Would clonal expansion and RNA changes persist if animals were taken off HFD and continued to age (Figures 2 and 5)? Findings may provide evidence that treating chronic injury can prevent the expansion of mutant hepatocytes and attenuate tumorigenesis.

Response to comment #7: We agree with the Referee that this is a highly relevant question. Determining whether restoration of tissue integrity can reverse the permissive state for *Ctnnb1*-mutant hepatocyte expansion will require future studies using models with strict temporal control over liver injury. In the current CMR^{tdT} model, withdrawal of HFD would not provide a clean test of this question because liver damage driven by the *MUP-uPA* transgene would persist even after diet normalization.

8. Considering the authors link clonal expansion and tumorigenesis of beta-catenin to activation of the AKT-NRF2 axis, treating animals with a pharmacological agent that inhibits NRF2 to suppress mutant hepatocyte expansion and tumorigenesis would enhance the manuscript's translational relevance.

Response to comment #8: We appreciate the Referee's suggestion, which is highly relevant from a translational perspective. However, pharmacological NRF2 inhibition in this model would require long-term treatment and experimentation, which is beyond the scope of this manuscript. Importantly, the manuscript already addresses the functional requirement for NRF2 using genetic deletion experiments, which show that NRF2 loss significantly delays *Ctnnb1*^{ex3} hepatocyte expansion and tumorigenesis. We therefore believe that pharmacologically targeting NRF2 is an important future direction for studies specifically focused on the therapeutic translation of these findings.

9. Related to this, is NRF2 activation cell intrinsic (activated in hepatocytes with beta-catenin mutation because of UPR and oxidative stress) or extrinsic (activated in tissue microenvironment because of chronic injury)? This needs to be clarified, because it seems the authors are trying to make both arguments.

Response to comment #9: We thank the Referee for highlighting this ambiguity. Our data support a model in which NRF2 activation is detected cell-intrinsically within *Ctnnb1*-mutant hepatocytes, but the strength and functional consequence of this intrinsic response are modulated by the surrounding liver tissue context. In the normal liver, *Ctnnb1*^{ex3} hepatocytes induce a limited NRF2 response, but this response is insufficient to prevent cellular attrition and elimination. In contrast, in chronic liver disease, the altered tissue environment permits a concerted AKT-NRF2 activation in Ctnnb1-mutant hepatocytes, promoting survival and expansion. Thus, we propose that the extrinsic tissue context modulates an intrinsic NRF2 switch in mutant hepatocytes. We have edited the text to clarify this concept:

"**A key determinant of the context-dependent fitness conferred by *Ctnnb1* activating mutations is an intrinsic NRF2 switch that operates differently depending on the normal versus diseased liver.**"

10. In the discussion, the authors state that tissue-level changes modify the repertoire and frequency of cellular signaling states in which mutations interact, opening new phenotypic opportunities. The discussion should expand on this premise and discuss the potential role of the tumor microenvironment in mutant hepatocyte expansion.

Response to comment #10: We thank the Referee for suggesting improvements to the discussion. We have now edited the text to expand upon this point as follows:

"**This demonstrates that oncogenic potential is not determined solely by the interaction between mutation and cell identity, but by how tissue-level changes modify the repertoire and frequency of cellular signaling states in which mutations interact, opening new phenotypic opportunities. Thus, fundamental changes in the microenvironment alter intrinsic cellular signaling cascades that enable this adaptive response. The apparent need for oncogenic β-catenin to cooperate with additional alterations in the normal liver does not reflect the need for more oncogenic substrate, but rather the scarcity of permissive cellular states within the intact liver.**"

11. Some of the data seems to be overinterpreted or speculative. For example, in the clustering analysis in Figure 3, the two clusters (Heps and Ctnnb1ex3) are distinct, yet part of the Heps cluster expresses Glul, Axin2, and other beta-catenin target genes. The authors classify these as 'transition'; however, because they are part of the Heps cluster, it is likely that they are normal zone 3 hepatocytes, which are constitutively active for beta-catenin and should be present in every single-cell RNA sequencing prep from normal liver. Additionally, there is no comparison between Ctnnb1ex3 hepatocytes and zone 3 hepatocytes in the same liver in terms of marker expression and pathways. Since zone 3 hepatocytes also have constant activation of beta-catenin, why do they not also undergo UPR and elimination?

Response to comment #11: We appreciate the opportunity to clarify this point. The transition cluster was defined as a population of tdTomato$^+$ *Ctnnb1*^{ex3} hepatocytes from the 1 month timepoint that co-express zone 3 markers together with periportal zone 1 markers, indicating an intermediate transcriptional state (**Fig. 3B** and **Fig. S6A**). To clarify this distinction, we include the relevant panels and manuscript text below:

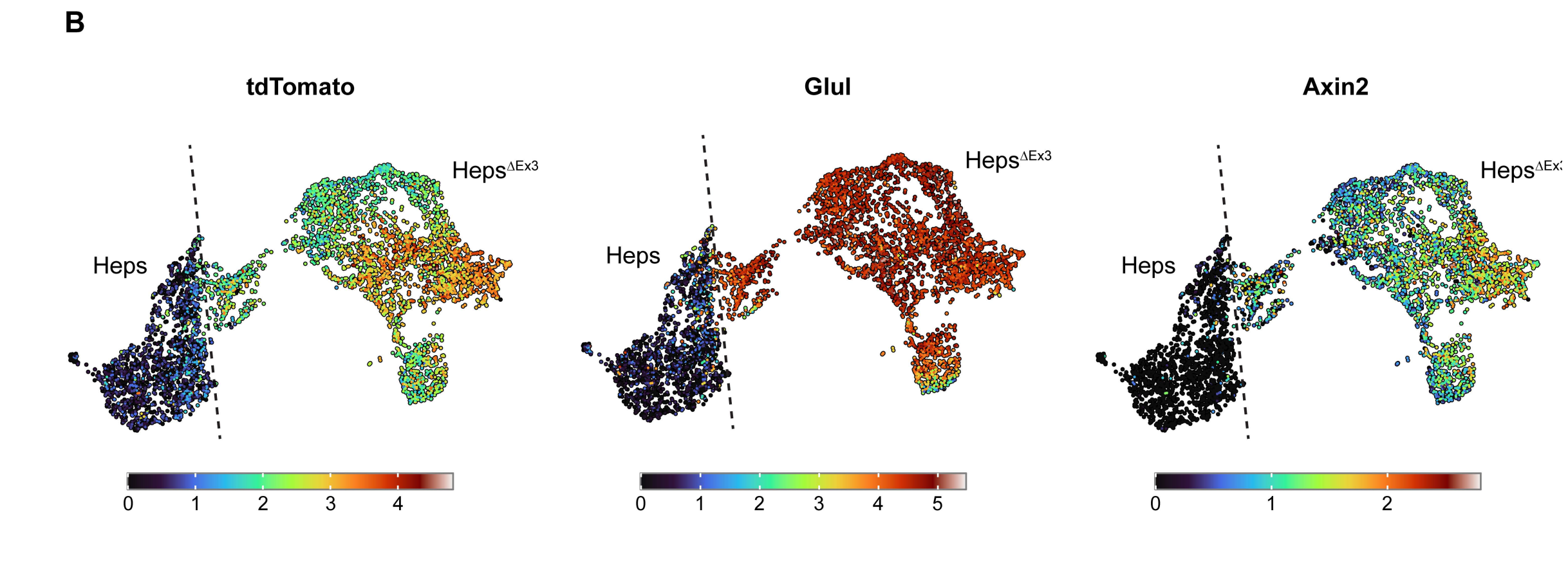

Fig. 3B. UMAP plots showing expression of tdTomato, *Glul* (GS), and *Axin2* from the dataset obtained as depicted in (A). The dotted line demarcates the boundary of WT hepatocytes and *Ctnnb1*^{ex3} hepatocytes.

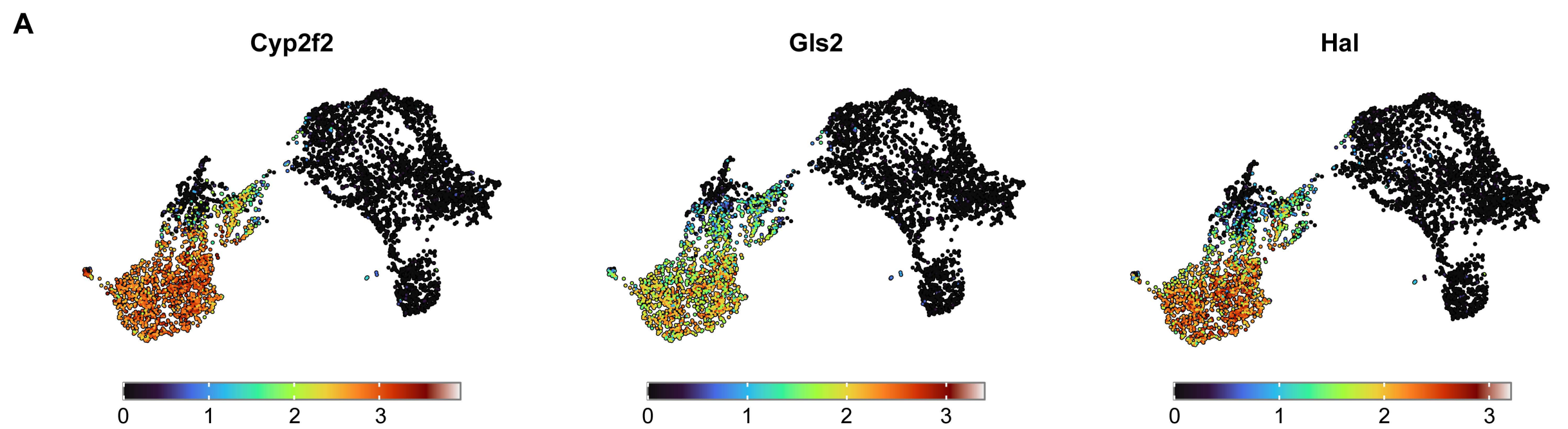

Fig. S6A. UMAP plots showing expression of *Cyp2f2*, *Gls2*, and *Hal* in the dataset in Fig. 3.

"The Transition cluster consists mainly of *Ctnnb1*^{ex3} hepatocytes (tdT^{+}) from the 1-month timepoint, which co-express markers of zone 3 hepatocytes (*Glul* and *Axin2*) along with typical periportal zone 1 markers (*Gls2* and *Hal*) (Fig. S6A). This co-expression pattern indicates that these *Ctnnb1*^{ex3} hepatocytes likely originated from zone 1 hepatocytes, which requires a longer transition toward the *Ctnnb1*^{ex3} hepatocyte state due to their greater initial transcriptomic difference from zone 2/3 hepatocytes. Their enrichment at the 1-month time point is consistent with a delayed adaptation process and the transient nature of this cluster."

In addition, endogenous zone 3 hepatocytes were present in the same dataset and are highlighted in **Fig. 3D** as tdTomato^{-} hepatocytes enriched for the zone 3 hepatocyte signature. This endogenous zone 3 population was used to compare physiologic β-Catenin activity with the transcriptional state induced by oncogenic β-Catenin activation in the normal liver, showing that oncogenic β-Catenin does not simply reproduce the physiologic zone 3 transcriptional program. The relevant manuscript text and figure panel is reproduced below:

"The Early cluster consists of *Ctnnb1*^{ex3} hepatocytes from the 1-month timepoint (Fig. 3C) and shows the strongest enrichment for canonical zone 3 hepatocyte markers. To confirm this, we applied a gene signature derived from normal mouse zone 3 hepatocytes and found that *Ctnnb1*^{ex3} hepatocytes in the Early cluster displayed hyperactivation of this signature, which progressively declined in the remaining *Ctnnb1*^{ex3} hepatocytes clusters (Fig. 3D). However, this hyperactivation was driven by just a subset of signature genes, suggesting that oncogenic β-catenin selectively

amplifies part of the zone 3 hepatocyte transcriptional program, while other subprograms typical of zone 3 hepatocyte remain unengaged (Fig. S6B)."

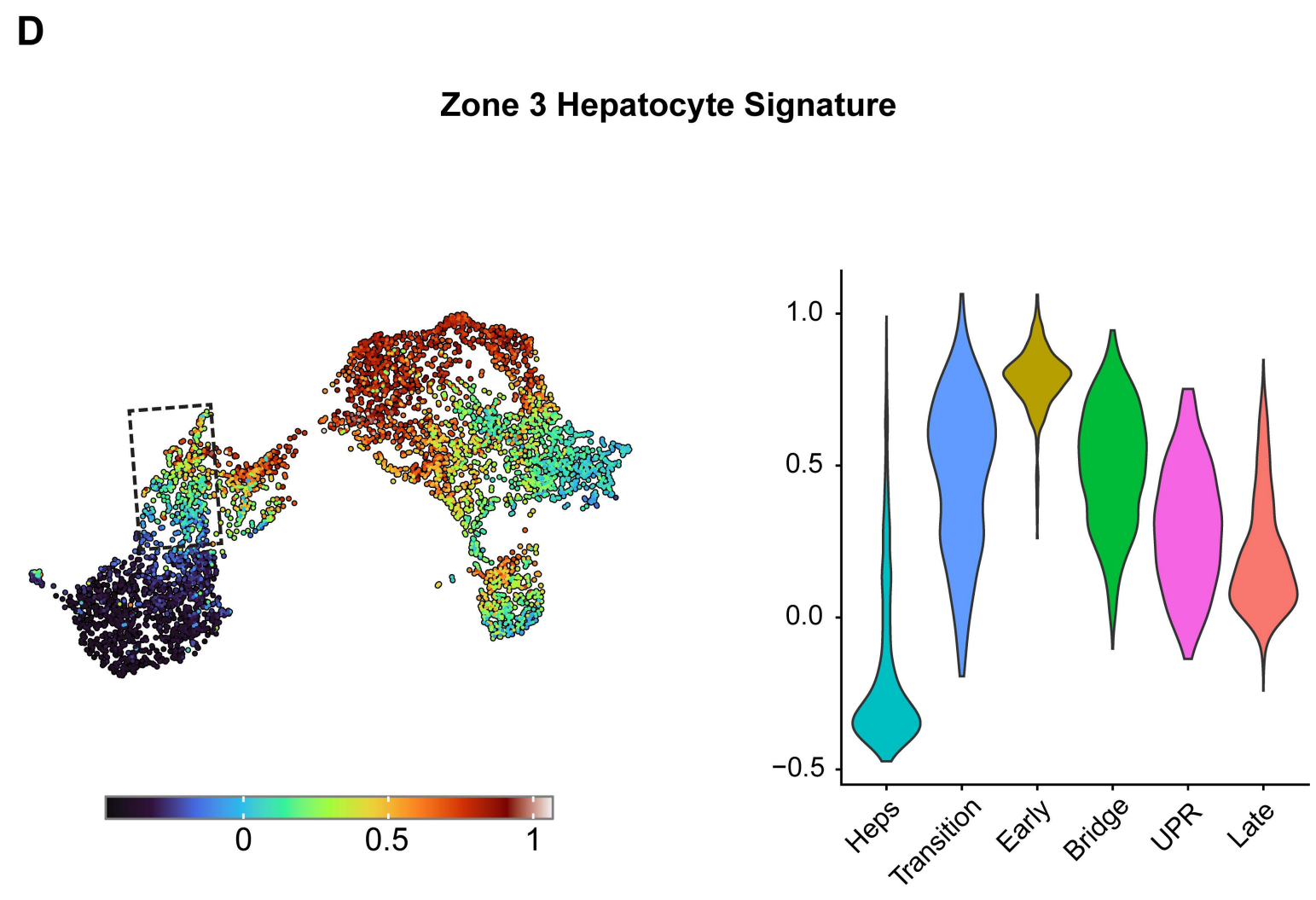

Fig. 3D. UMAP plot (left) showing enrichment of a zone 3 gene signature and relative enrichment levels for each cluster identified in Fig. 3C depicted in a violin plot (right). The dotted box on the UMAP plot highlights endogenous zone 3 hepatocytes.

12. The conclusion that the stress-resilient state with upregulation of multiple signaling pathways makes Ctnnb1ex3 hepatocytes more susceptible needs additional justification and confirmation. The stress-resilient state also needs to be better defined; if these cells are activating pathways that resist stress, why are they being eliminated?

Response to comment #12: We apologize for the confusion on this matter. The intent was not to imply that this state fully protects *Ctnnb1^{ex3}* hepatocytes from stress-induced elimination. We used this term to describe a prolonged process in which mutant hepatocytes activate compensatory stress-response pathways that are unable to resolve the elevated ROS, ER stress, and UPR activation induced by constitutive oncogenic β-catenin activity. These pathways delay but do not prevent progressive attrition and eventual elimination.

To avoid this potential confusion, we have changed "stress-resilient" to "stress-adapted" and have edited the text as follows:

"**Taken together, these observations suggest that oncogenic β-catenin induces an initial mis-reprogramming of hepatocytes into a hyperactive zone 3-like state. This state leads to ER stress, UPR activation, and the collapse of mature hepatocyte identity with the emergence of an ineffective, stress-adapted, "limbo" state before prolonged cellular attrition and eventual elimination.**"

13. Cell processes such as senescence and apoptosis should also be analyzed in the Late Ctnnb1ex3 hepatocytes to elucidate the mechanism by which they are eliminated.

Response to comment #13: We thank the Referee for this suggestion. To directly compare the Late cluster with other *Ctnnb1^{ex3}* clusters in the CMR^{tdT} scRNAseq dataset, we analyzed markers for cellular stress, growth arrest, senescence, and apoptotic priming (**Fig. S9A**). Similar to the UPR cluster, the late cluster showed elevated expression of cellular stress markers (*Fos*, *Jun*, *Atf3*, *Ddit3*) and growth arrest markers

(*Trp53*, *Cdkn1a*, *Gadd45a*) compared to normal hepatocytes and to the Lipogenic, Antioxidant, and Cycling clusters. However, this analysis did not reveal a dominant senescence or apoptotic signature that could clearly explain their elimination.

Because these cells shrink dramatically over time, we next performed ultrastructural analysis by electron microscopy. Hepatocytes from 3-month CR^{tdT} mice showed a striking accumulation of autolysosome-like structures specifically in small tdT^{+} cells, which was not observed in tdT^{-} hepatocytes or normal-sized tdT^{+} hepatocytes (**Fig. S9B-C**). These findings suggest that prolonged activation of autophagy/lysosomal pathways accompanies the late cellular attrition and may contribute to the progressive elimination of *Ctnnb1^{ex3}* hepatocytes.

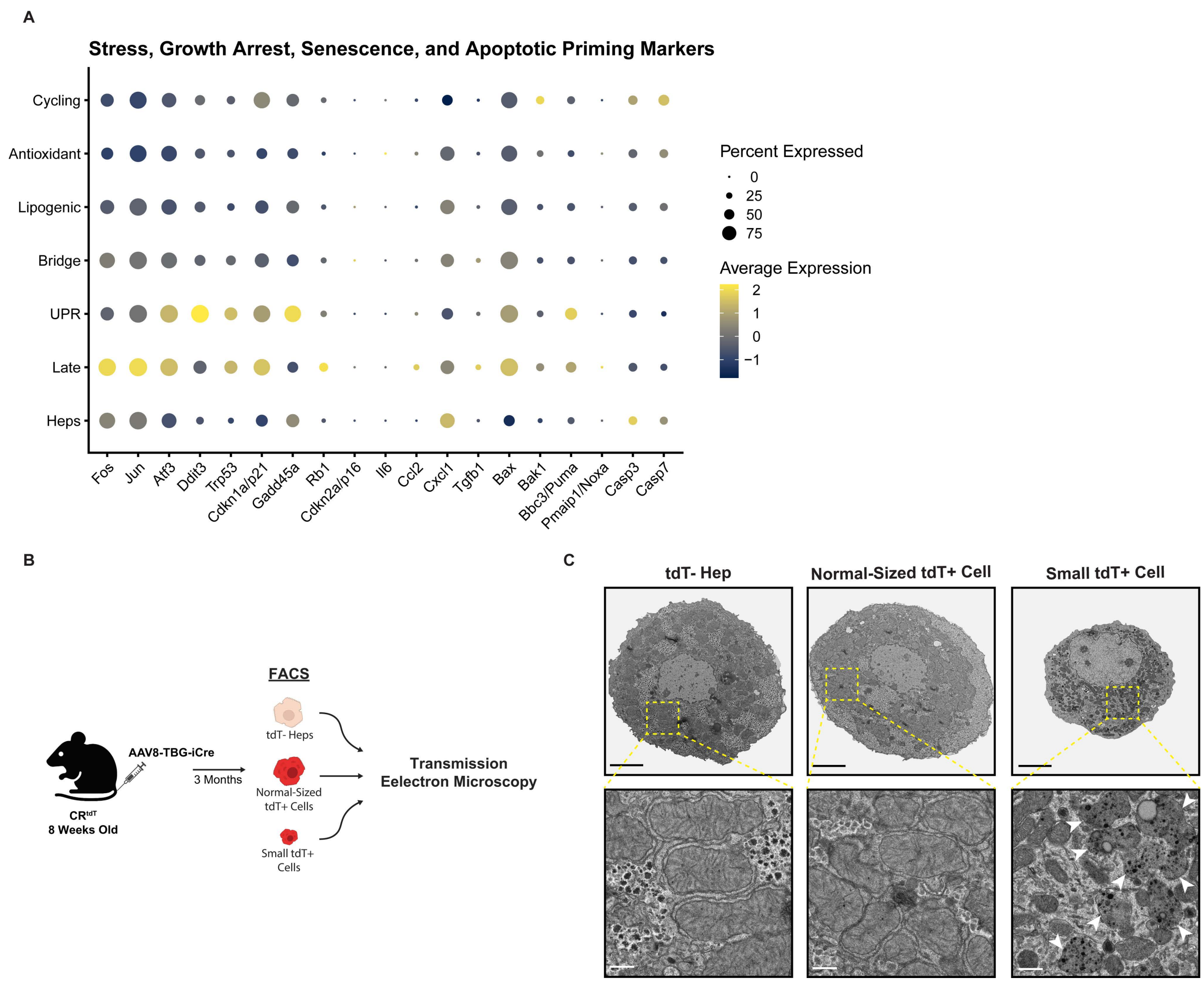

Fig. S9. Cellular stress pathways in *Ctnnb1^{ex3}* hepatocytes. A) Dot plot of markers for cellular stress, growth arrest, senescence, and apoptotic priming in Ctnnb*1^{ex3}* hepatocytes from CMR^{tdT} mice. **B)** Schematic of experimental approach to image hepatocytes by electron microscopy. 3 months post-AAV injection in a CR^{tdT} mouse, hepatocytes were isolated and FACS sorted to separate tdT^{-} hepatocytes, Normal-Sized tdT^{+}, and Small tdT^{+} cells. Each cell population was fixed and imaged by transmission electron microscopy. **C)** Electron microscopy images of representative cells from the three populations isolated. Yellow-dashed boxes indicate the area displayed in higher magnification. Arrowheads highlight autolysosome structures. Findings were confirmed by imaging two independent mouse samples. Black scale bars = 4 μm, White scale bars = 0.5 μm.

[Editorial note: panel b created in BioRender. Hewett, C. (2026) https://BioRender.com/5pwn7dp]

14. The mutational status of Nfe2l2 should be assessed in CMRtdT tumors to determine whether NRF2 activation occurs through mutation in the context of chronic injury or beta-catenin dependent changes in cellular fitness.

Response to comment #14: We agree with this excellent suggestion from the Referee. Our whole-exome sequencing data (**Fig. S4**) revealed no somatic SNVs or indels in the coding or splice-region sequence of *Nfe2l2* in any of the tumors analyzed. This result was confirmed by independent BAM-level pileup inspection of *Nfe2l2* coding/splice target regions, which found no convincing tumor-specific alternate allele. Together, these analyses support the conclusion that NRF2 activation in CMR^{tdT} tumors is not caused by activating mutations in the *Nfe2l2* gene.

Minor:

1. The figure legend for Figure 1C doesn't match the figure panel. Only average points appear in the bar graphs. No individual points are shown.

Response to minor comment #1: We thank the Referee for pointing out this mistake in the figure legend. The legend has now been updated to correctly explain the meaning of the points on the graph.

2. The inclusion of dot plots in Supplemental Figure 3 would help demonstrate the loss of hepatocyte identity and changes in signaling pathways in the Bridge, UPR, and Late clusters.

Response to minor comment #2: To address this comment, we added a violin plot (**Fig. S6D**) showing the expression level and distribution of the pan-hepatocyte signature used in **Fig. 3F**.

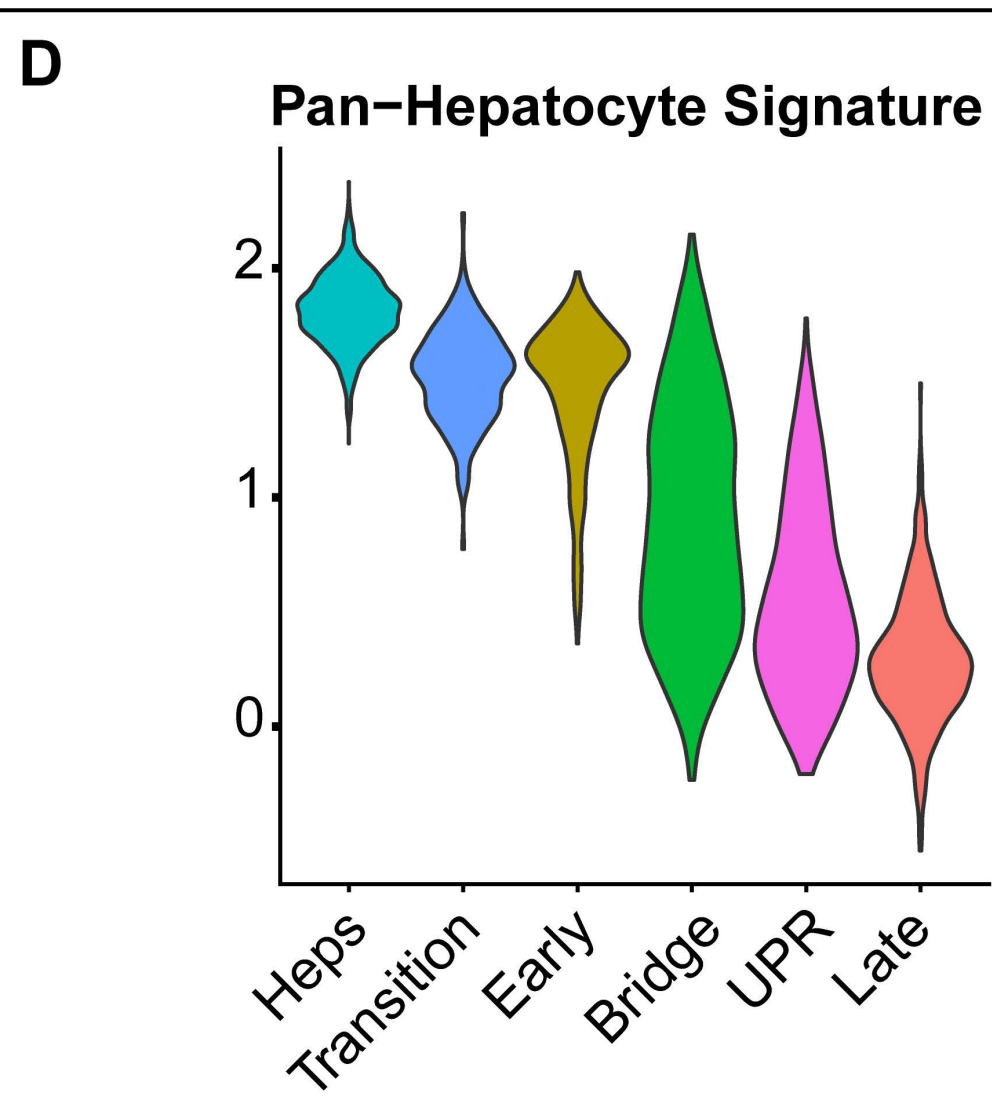

Fig. S6D. Violin plots of relative pan-hepatocyte gene signature expression for each cluster identified in Fig. 3C.

3a. The mouse models employed lack description. Mouse strain and sex need to be described in the methods and shown in figures.

Response to minor comment #3a: We apologize for this omission. We have updated the Methods section to include sex and genetic background of ten mice used in this study, as follows: "Male mice were used for all experiments, and each mouse strain was maintained on the C57BL/6 background. All mice were obtained from their cited sources: *ROSA26*$^{LSL\text{-}tdTomato/+}$ (Jackson Laboratory, #007909), *Ctnnb1*$^{ex3fl/+}$ [10], *MUP-uPA*[11], and *Nfe2l2*$^{fl/fl}$ (Jackson Laboratory, #025433)."

3b. Would hormonal changes over time have a role in beta-catenin's oncogenicity?

Response to minor comment #3b: This is an interesting possibility. Age-associated hormonal changes, including sex hormones, insulin or glucocorticoids, could influence β-catenin oncogenicity by altering liver tissue physiology, inflammatory responses and regenerative capacity. Thus, hormonal remodeling may be an important component of the shifting liver fitness landscape during aging and chronic liver disease.

Referee #4:

I co-reviewed this manuscript with one of the referees who provided the listed reports. This is part of the Nature Communications initiative to facilitate training in peer review and to provide appropriate recognition for Early Career Researchers who co-review manuscripts.

We thank the Referee for contributing to the evaluation of our manuscript and providing input.

Response to Referees References:

1 Lai, R. *et al*. Incidence rates of hepatocellular carcinoma based on risk stratification in steatotic liver disease for precision medicine: A real-world longitudinal nationwide study. *PLoS Med* **21**, e1004479 (2024). https://doi.org/10.1371/journal.pmed.1004479

2 Institute, N. C. *SEER Cancer Stat Facts: Liver and Intrahepatic Bile Duct Cancer*, <https://seer.cancer.gov/statfacts/html/livibd.html> (

3 Harada, N. *et al*. Lack of tumorigenesis in the mouse liver after adenovirus-mediated expression of a dominant stable mutant of beta-catenin. *Cancer Res* **62**, 1971-1977 (2002).

4 Lemberger, U. J. *et al*. Hepatocyte specific expression of an oncogenic variant of beta-catenin results in cholestatic liver disease. *Oncotarget* **7**, 86985-86998 (2016). https://doi.org/10.18632/oncotarget.13521

5 Liu, F. *et al*. Oncogenic beta-catenin stimulation of AKT2-CAD-mediated pyrimidine synthesis is targetable vulnerability in liver cancer. *Proc Natl Acad Sci U S A* **119**, e2202157119 (2022). https://doi.org/10.1073/pnas.2202157119

6 Loesch, R. *et al*. Deleting the beta-catenin degradation domain in mouse hepatocytes drives hepatocellular carcinoma or hepatoblastoma-like tumor growth. *J Hepatol* **77**, 424-435 (2022). https://doi.org/10.1016/j.jhep.2022.02.023

7 Brunner, S. F. *et al*. Somatic mutations and clonal dynamics in healthy and cirrhotic human liver. *Nature* **574**, 538-542 (2019). https://doi.org/10.1038/s41586-019-1670-9

8 Ng, S. W. K. *et al*. Convergent somatic mutations in metabolism genes in chronic liver disease. *Nature* **598**, 473-478 (2021). https://doi.org/10.1038/s41586-021-03974-6

9 Dow, M. *et al*. Integrative genomic analysis of mouse and human hepatocellular carcinoma. *Proc Natl Acad Sci U S A* **115**, E9879-E9888 (2018). https://doi.org/10.1073/pnas.1811029115

10 Harada, N. *et al*. Intestinal polyposis in mice with a dominant stable mutation of the beta-catenin gene. *EMBO J* **18**, 5931-5942 (1999). https://doi.org/10.1093/emboj/18.21.5931

11 Weglarz, T. C., Degen, J. L. & Sandgren, E. P. Hepatocyte transplantation into diseased mouse liver. Kinetics of parenchymal repopulation and identification of the proliferative capacity of tetraploid and octaploid hepatocytes. *Am J Pathol* **157**, 1963-1974 (2000). https://doi.org/10.1016/S0002-9440(10)64835-3

We thank the Referees for their continued consideration of our manuscript. Please find the response to the remaining comment below.

Reviewer #2 (Remarks to the Author):

The authors have satisfactorily addressed my concerns regarding the manuscript by providing additional data that strengthen the mechanistic insights and clarify several aspects of the study. One minor point remains regarding the immunostaining presented in Figure S11B. The inclusion of a negative control (e.g., incubation with the secondary antibody alone, without the primary antibody) would facilitate the interpretation of this panel, as the strong green autofluorescence known to be present in the liver appears to interfere with the specific signal. Overall, I believe that the authors have adequately addressed the issues raised during the review process.

We appreciate the Referee's positive assessment of the revised manuscript and for the helpful suggestion regarding the Supplementary figure 11B.

To address this point, we added the no-primary control to the panel (**Supplementary Figure 11B**).

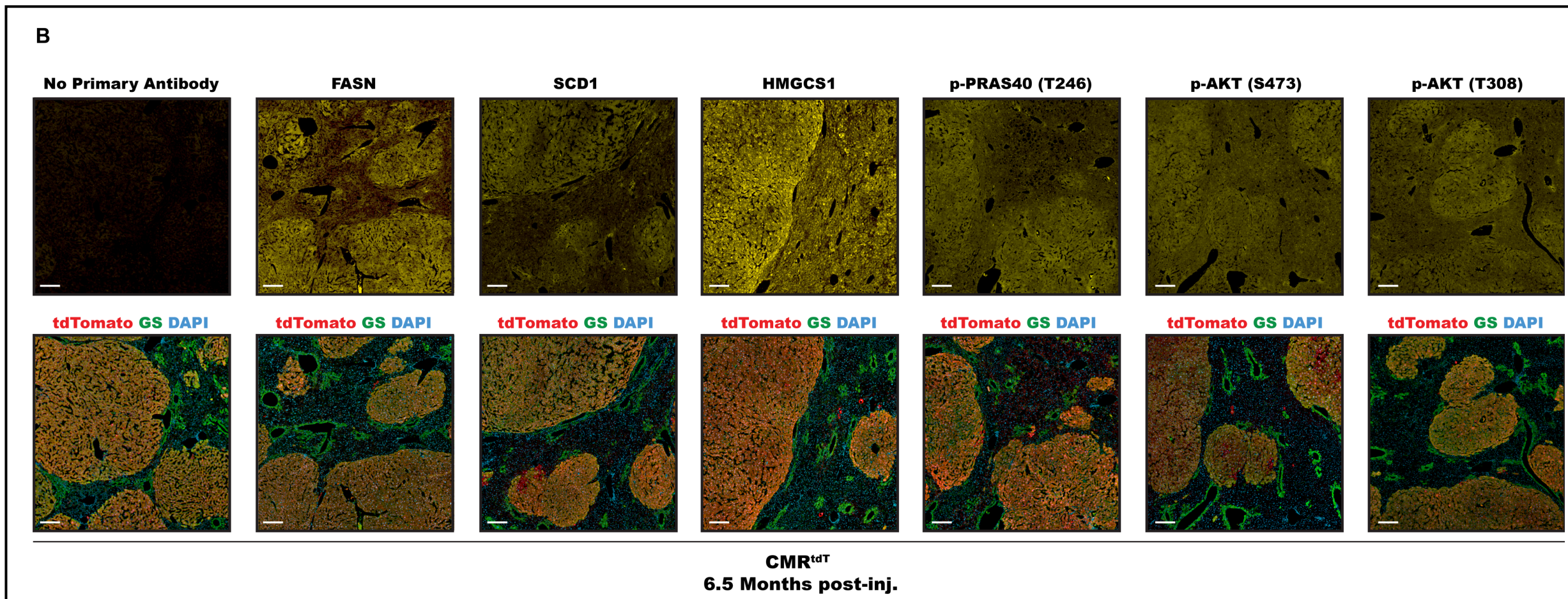

Fig. S11B. Representative immunofluorescence images of CMR^{tdT} liver 6.5 months post-AAV injection stained with tdT, GS, and either FASN, SCD1, HMGCS1, PRAS40, p-AKT (S473), or p-AKT (T308). Scale = 200 µm.
